# Unexpected Methyllanthionine Stereochemistry in the Morphogenetic Lanthipeptide SapT

**DOI:** 10.1021/jacs.2c00517

**Published:** 2022-03-30

**Authors:** Raymond Sarksian, Julian D. Hegemann, Max A. Simon, Jeella Z. Acedo, Wilfred A. van der Donk

**Affiliations:** † Department of Chemistry and Howard Hughes Medical Institute, University of Illinois at Urbana−Champaign, Urbana, Illinois 61822, United States; ‡ Helmholtz Institute for Pharmaceutical Research Saarland (HIPS), Helmholtz Centre for Infection Research (HZI), Saarland University Campus, 66123 Saarbrücken, Germany; § Department of Bioengineering and Carl R. Woese Institute for Genomic Biology, University of Illinois at Urbana−Champaign, Urbana, Illinois 61822, United States

## Abstract

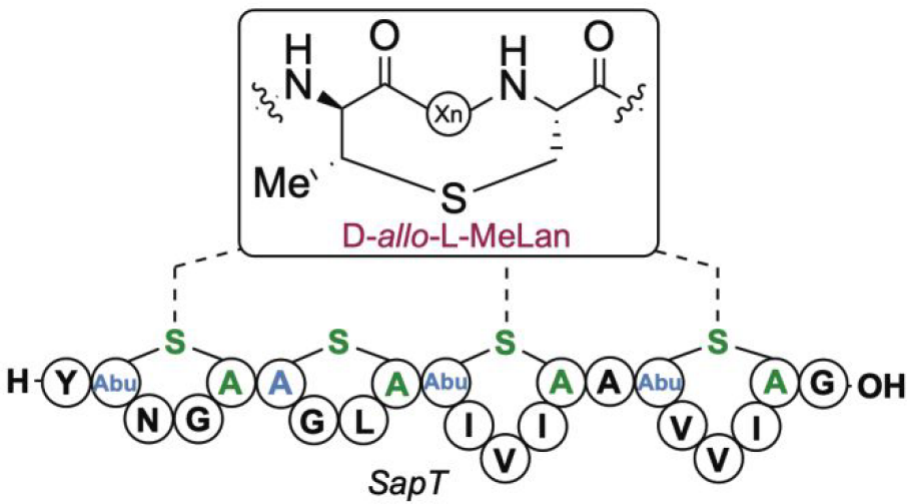

Lanthipeptides are polycyclic peptides characterized by the presence of lanthionine (Lan) and/or methyllanthionine (MeLan). They are members of the ribosomally synthesized and post-translationally modified peptides (RiPPs). The stereochemical configuration of (Me)Lan cross-links is important for the bioactivity of lanthipeptides. To date, MeLan residues in characterized lanthipeptides have either the 2*S*,3*S* or 2*R*,3*R* stereochemistry. Herein, we reconstituted in *Escherichia coli* the biosynthetic pathway toward SapT, a class I lanthipeptide that exhibits morphogenetic activity. Through the synthesis of standards, the heterologously produced peptide was shown to possess three MeLan residues with the 2*S*,3*R* stereochemistry (d-*allo*-l-MeLan), the first time such stereochemistry has been observed in a lanthipeptide. Bioinformatic analysis of the biosynthetic enzymes suggests this stereochemistry may also be present in other lanthipeptides. Analysis of another gene cluster in *Streptomyces coelicolor* that is widespread in actinobacteria confirmed another example of d-*allo*-l-MeLan and verified the bioinformatic prediction. We propose a mechanism for the origin of the unexpected stereochemistry and provide support using site-directed mutagenesis.

## Introduction

Ribosomally synthesized and post-translationally modified peptides (RiPPs) are biosynthesized using a common logic.^1,2^ Their biosynthesis starts with the ribosomal production of a precursor peptide that commonly consists of an N-terminal leader region and C-terminal core region. The leader peptide often functions as a handle for recruitment of biosynthetic enzymes, and the core peptide region is enzymatically modified by post-translational modifications. Leader peptide removal yields the mature RiPP.^1−3^

**Figure 1:**
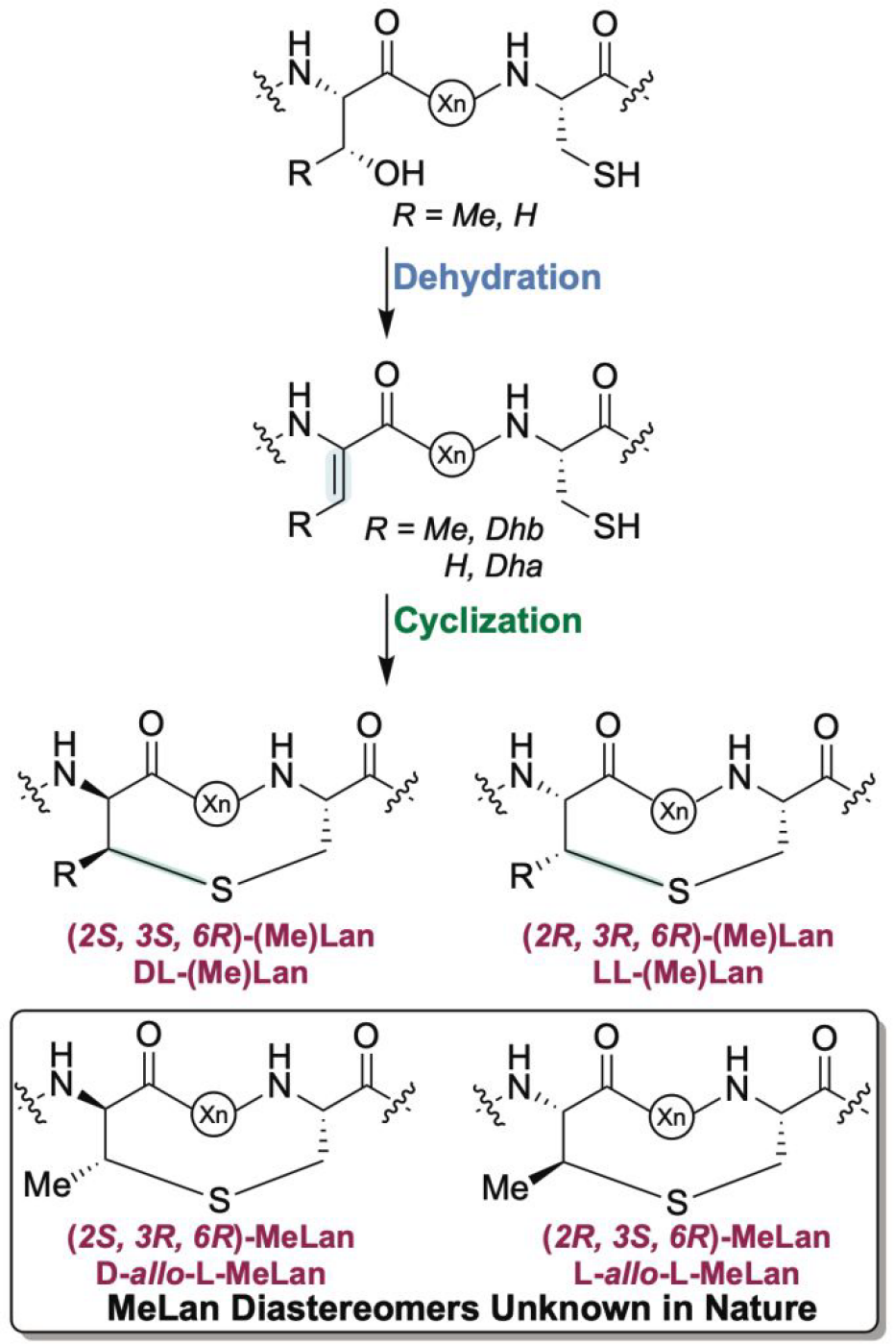
Maturation of lanthipeptides proceeds through the dehydration of Ser/Thr residues to generate dehydroalanine (Dha) or dehydrobutyrine (Dhb). Subsequent cyclization via Michael-type addition by Cys residues with net *anti*-stereochemistry across (*Z*)-Dhb yields the two MeLan diastereomers observed to date.

Lanthipeptides represent one of the largest families of RiPPs. They are defined by the presence of lanthionine (Lan) and/or methyllanthionine (MeLan) residues.^3,4^ Five classes of lanthipeptides are currently known that differ in the biosynthetic enzymes used to produce the (Me)Lan structures. The biosynthesis of lanthipeptides involves the dehydration of Ser/Thr residues followed by subsequent cyclization of Cys residues onto the dehydroalanines (Dha) or dehydrobutyrines (Dhb) to generate (Me)Lans (Figure 1). To date, the observed stereochemistry of Dhb and MeLan moieties requires the *anti*-elimination of activated Thr residues to generate a (*Z*)-Dhb intermediate followed by a subsequent *anti*-addition of l-Cys across the dehydroamino acid to yield the MeLan residue (Figure 1).^4^

The stereochemical configuration of (Me)Lan residues in lanthipeptides has been shown to be important for their antimicrobial activity.^5,6^ All characterized lanthipeptides containing MeLan moieties exhibit either (2*S*,3*S*,6*R*)- or (2*R*,3*R*,6*R*)- configurations, hereafter referred to as dl- and ll-MeLan (Figure 1).^4^ The dl-configuration has been traditionally observed for many lanthipeptides including nisin, epidermin, subtilin, and Pep5.^4^ Only recently have a select number of lanthipeptides been demonstrated to contain MeLan with the ll-configuration. They are formed predictably in certain circumstances such as substrate-controlled cyclizations.^7−13^ The (2*S*,3*R*,6*R*)- and (2*R*,3*S*,6*R*)-MeLan diastereomers, referred to herein as d-*allo*-l-MeLan and l-*allo*-l-MeLan, have yet to be observed in lanthipeptides (Figure 1).

**Figure 2:**
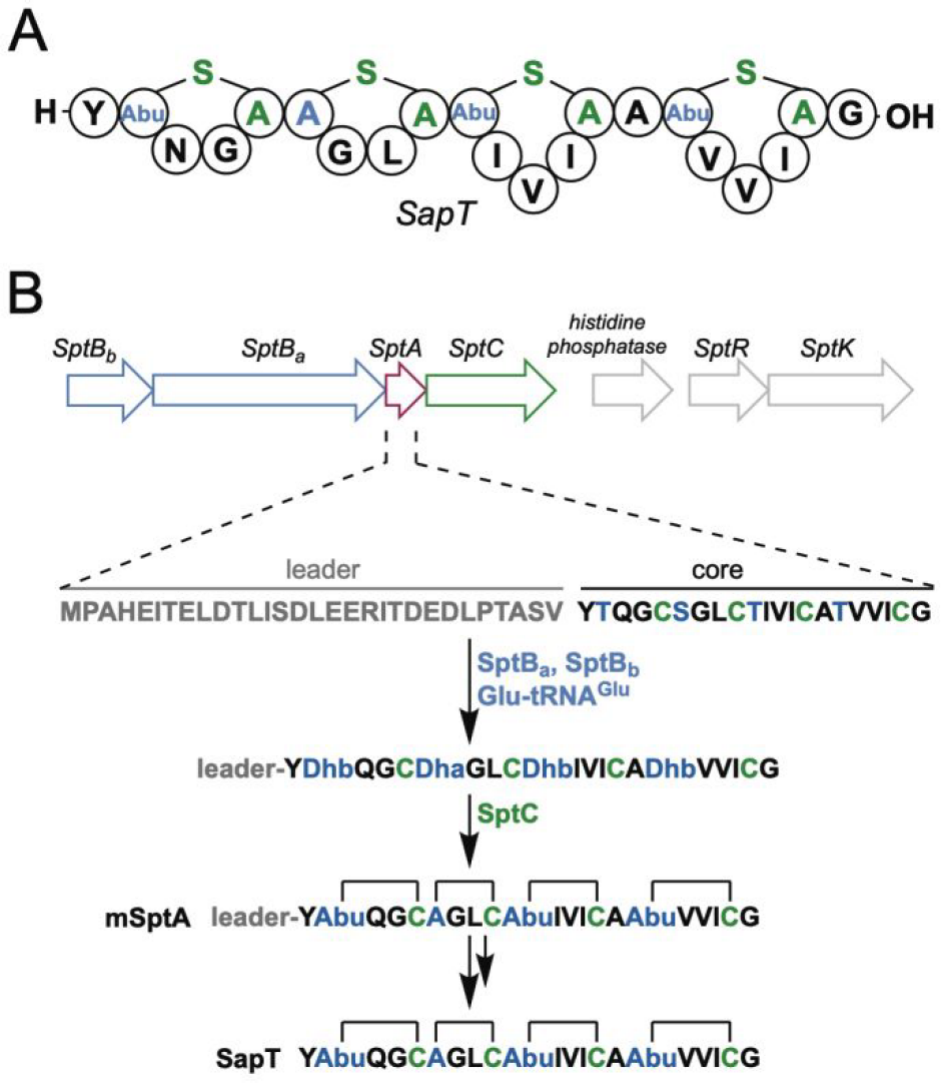
(A) Schematic structure of the morphogenetic lanthipeptide SapT featuring one Lan and three MeLan residues. (B) The *spt* BGC produces the class I lanthipeptide SapT, as discussed in this work. The SptA peptide consists of a leader peptide that is removed during maturation and a core peptide that is converted to SapT. A protease is not encoded in the SapT BGC, and the mature lanthipeptide may be released from the precursor by a protease outside the BGC.^3,54^ Abu, 2-aminobutyric acid.

In addition to antimicrobial activity, select lanthipeptides possess antifungal, antiviral, antiallodynic, and morphogenetic activities.^14−20^ SapT is a partially characterized peptide that was isolated from *Streptomyces lavenduligriseus* Tü901 (formerly known as *S. tendae* Tü901; see Supporting Information) (Figure 2).^16^ The ring pattern of SapT was deduced by nuclear magnetic resonance spectroscopy and tandem mass spectrometry (MS), but the stereochemistry of its (Me)Lan residues was not determined.^16^ SapT, like the class III lanthipeptide SapB, which is produced by *Streptomyces coelicolor*, possesses morphogenetic properties by functioning as a biosurfactant.^15,16,21^ The peptides were not initially classified as lanthipeptides, and Sap historically stands for s*pore*a*ssociated*p*rotein*.^22^ SapB is involved in the early developmental process of the filamentous bacterium *S. coelicolor*.^15,21,22^ The compound promotes the formation of aerial mycelia by reducing the surface tension between the colony/air interface, thereby initiating spore formation.^15,21,22^ SapT has been shown to restore the ability of SapB-deficient *S. coelicolor* to undergo morphogenesis, which established SapT as a biosurfactant.^16^

In the absence of a known biosynthetic gene cluster (BGC) and based on the functional similarities to SapB, SapT was initially proposed to also belong to the class III lanthipeptides, which are matured by LanKC enzymes.^23^ However, previous bioinformatic analysis identified several putative class I lanthipeptides with sequences similar to SapT.^4^ Confirmation of the SapT biosynthetic pathway would enable establishment of a heterologous production route, which would in turn facilitate structure–activity relationship studies and the elucidation of the stereochemistry of the (Me)Lan moieties.

Herein, we demonstrate that a class I lanthipeptide BGC from *S. lavenduligriseus* Tü901, referred to as the *spt* locus, is responsible for the production of SapT (Figure 2). We show that SptB_a_, SptB_b_, and SptC convert the precursor peptide SptA to a fully cyclized peptide during coexpression in *E. coli*. High resolution and tandem MS data of the heterologous product are consistent with the structure of SapT isolated from *S. lavenduligriseus* Tü901.^16^ Using synthetic standards, the dl-Lan configuration was assigned to the single Lan in SapT, whereas the three MeLan residues were demonstrated to have the d-*allo*-l-MeLan configuration. This observation represents the first time such a configuration has been reported for MeLan in a lanthipeptide. Mechanistic possibilities to account for the unusual product stereochemistry are discussed, and a bioinformatic prediction is made for other class I lanthipeptides encoded in the bacterial genomes that are likely to contain *allo* stereochemistry. This prediction is based on sequence and structure analysis of the dehydratase. For one representative example from a widespread group of BGCs, this prediction was experimentally verified. Thus, *allo*-l-MeLan occurrence is common and can be predicted based on the sequence of the dehydratase.

## Results

### Identification of the SapT Biosynthetic Gene Cluster

*S. lavenduligriseus* Tü901 was obtained from the American Type Culture Collection (ATCC, strain identifier ATCC 31160, listed as *Streptomyces tendae* Tü901). The strain was reactivated and cultivated, genomic DNA was isolated and sequenced, and the data were deposited in GenBank (genome accession number CP072000). The strain was reclassified in this study because analysis of its sequenced genome revealed that the strain is much more closely related to the type strain of *S. lavenduligriseus* than the type strain of *S. tendae*. As expected for a *Streptomyces* strain, *S. lavenduligriseus* Tü901 is a talented secondary metabolite producer. Analysis with AntiSmash 6.0^24^ yielded a total of 45 putative natural product BGCs, including several NRPS, PKS, terpenoid, and RiPP gene clusters. Besides three lanthipeptide BGCs, other clusters relating to characterized RiPP subfamilies include two lasso peptide BGCs. One of the three identified lanthipeptide BGCs encodes a precursor peptide (SptA) whose core region correlates exactly to the primary sequence of SapT (Figure 2B). Further inspection of this cluster confirms the notion that SapT is not a class III lanthipeptide. Instead, SapT is a class I lanthipeptide, belonging to a rare class I subtype featuring a split LanB protein. For this subtype, the N-terminal glutamylation and C-terminal elimination domains typically found in one polypeptide for LanB dehydratases are encoded as two distinct proteins. Such “split” LanB proteins are common in thiopeptide biosynthesis,^25^ but thus far only one lanthipeptide has been shown to be formed by a split LanB.^14^ The two predicted subunits of the split LanB enzyme were anticipated to function like full-length class I lanthipeptide dehydratases that activate Ser/Thr residues through a transesterification reaction utilizing glutamyl-tRNA^Glu^ as a cosubstrate.^26−29^ Here, SptB_a_ would catalyze the glutamylation reaction and SptB_b_ would catalyze the glutamate elimination to generate the corresponding Dha/Dhb moieties. The BGC also encodes a lanthipeptide cyclase, SptC (Figure 2).

### Heterologous Production of SapT

To connect the genetic information in the BGC to the natural product, we established the heterologous production of SapT in *E. coli*. To allow simultaneous expression of SptA, SptB_a_, SptB_b_, and SptC, the compatible vectors pRSF-Duet and pCDF-Duet were chosen (Figure S1). SptA was expressed with an N-terminal His_6_-tag whereas no tags were introduced to the processing enzymes to facilitate the isolation of the modified precursor. However, after performing Ni-affinity chromatography, His_6_-SptA could not be detected by mass spectrometry (MS) in the unmodified or fully modified form or in intermediate modification states.

**Figure 3:**
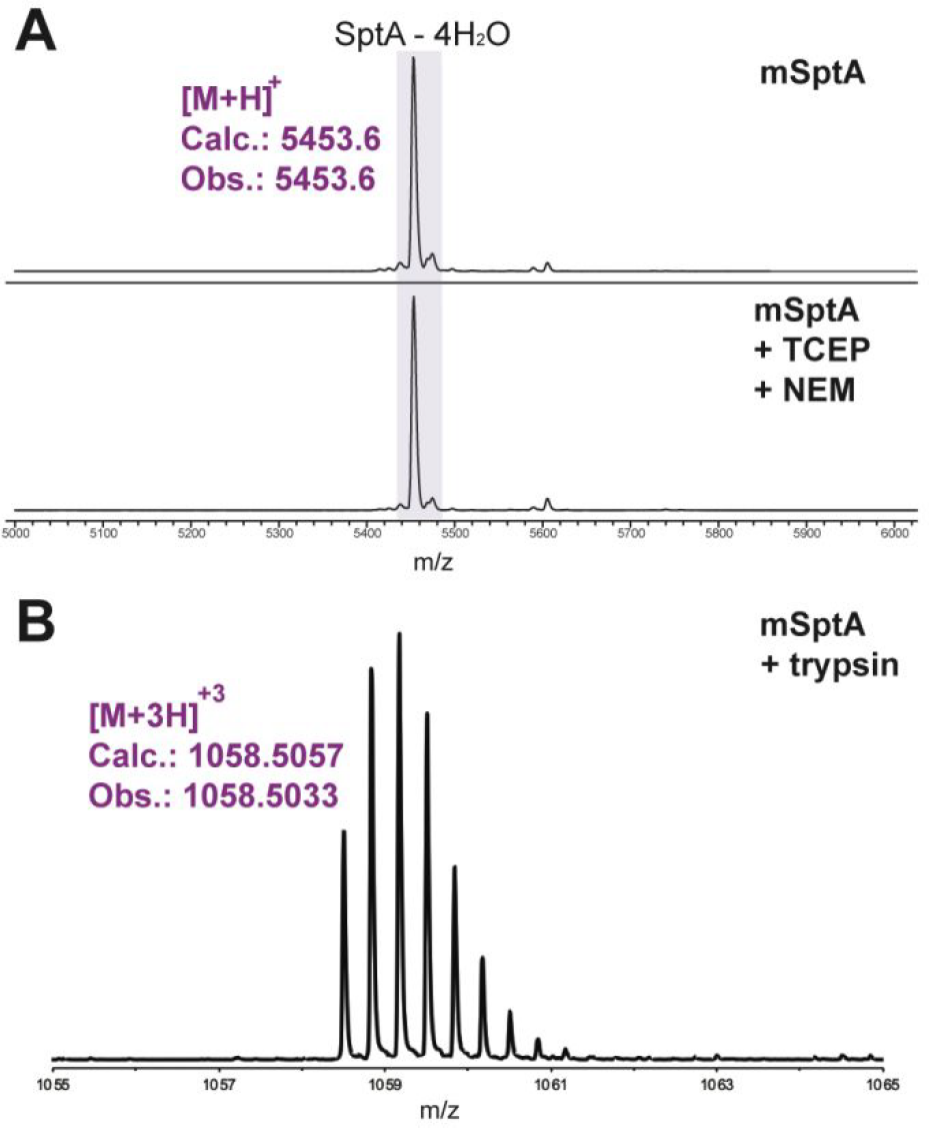
MS analysis of mSptA. (A) MALDI-TOF MS of mSptA (top) and the results of an NEM alkylation assay. Lack of NEM adducts suggests the peptide is fully cyclized. (B) High resolution ESI-MS analysis of the C-terminal fragment of mSptA after trypsin digestion.

Based on previous heterologous production of lanthipeptides in *E. coli*, the failure to obtain the modified precursor could have several reasons. The tRNA^Glu^ sequence in *S. lavenduligriseus* Tü901 is different from that in *E. coli* at positions that have previously been shown to be important for recognition by LanB enzymes (Table S2).^29^ The noncompatibility of the *E. coli* tRNA^Glu^ with LanB enzymes from actinobacteria was overcome previously by coexpression of the glutamyl-tRNA synthetase/tRNA^Glu^ pair from *Thermobispora bispora*.^25^ An additional reason for the unsuccessful production of modified SptA could involve degradation of the precursor peptide in *E. coli* as commonly observed for class IV lanthipeptides from actinobacteria.^30−33^ We therefore tested various iterations of the production system with and without coexpression of the *T. bispora* glutamyl-tRNA synthetase/tRNA^Glu^ pair and use of a His_6_-maltose binding protein (MBP)-SptA fusion to prevent degradation. In the latter case, treatment with tobacco etch virus (TEV) protease after Ni-affinity chromatography was used to release Ser-SptA for MS analysis. Only when using both the His_6_-MBP-tagged precursor peptide and *T. bispora* GluRS and tRNA^Glu^ was a mass of fourfold dehydrated SptA observed (Figures 3 and S1). A peptide that was truncated at the C-terminus was also detected (Figure S1) suggesting that proteolysis in *E. coli* competes with the installation of the rings, which appears to protect the modified peptide against proteolytic degradation.

To confirm that not only the four desired dehydrations but also the mass-neutral cyclizations had occurred, a modification assay with the thiol selective electrophile *N*-ethylmaleimide (NEM) was performed. The lack of NEM addition (Figure S1) is consistent with all Cys residues being involved in (Me)Lan formation. Following recommended nomenclature,^34^ the fully cyclized precursor isolated from *E. coli* will be referred to herein as modified SptA (mSptA).

To isolate full-length mSptA, heterologous coexpression in *E. coli* was carried out on a 6 L scale resulting in 0.5 mg of mSptA per liter of culture after isolation by Ni-affinity chromatography with an on-column TEV protease cleavage step, and subsequent HPLC purification. Taken together, these experiments provide the link between the *spt* locus and SapT. They further show that the glutamylated tRNA^Glu^ from *E. coli* is not a viable substrate for SptB_a_.

### Initial Stereochemical Analysis

Stereochemical analysis was initially performed by gas-chromatography mass spectrometry (GC-MS) with a chiral stationary phase.^8,9,13,35^ The fourfold dehydrated and cyclized product was hydrolyzed in 6 M DCl/D_2_O to release the (Me)Lan residues. The hydrolysate was then treated with acetyl chloride and methanol to convert the carboxylic acids to methyl esters. Finally, the primary amines were acylated with pentafluoropropionic anhydride in dichloromethane to yield volatile pentafluoropropionamides.

**Figure 4:**
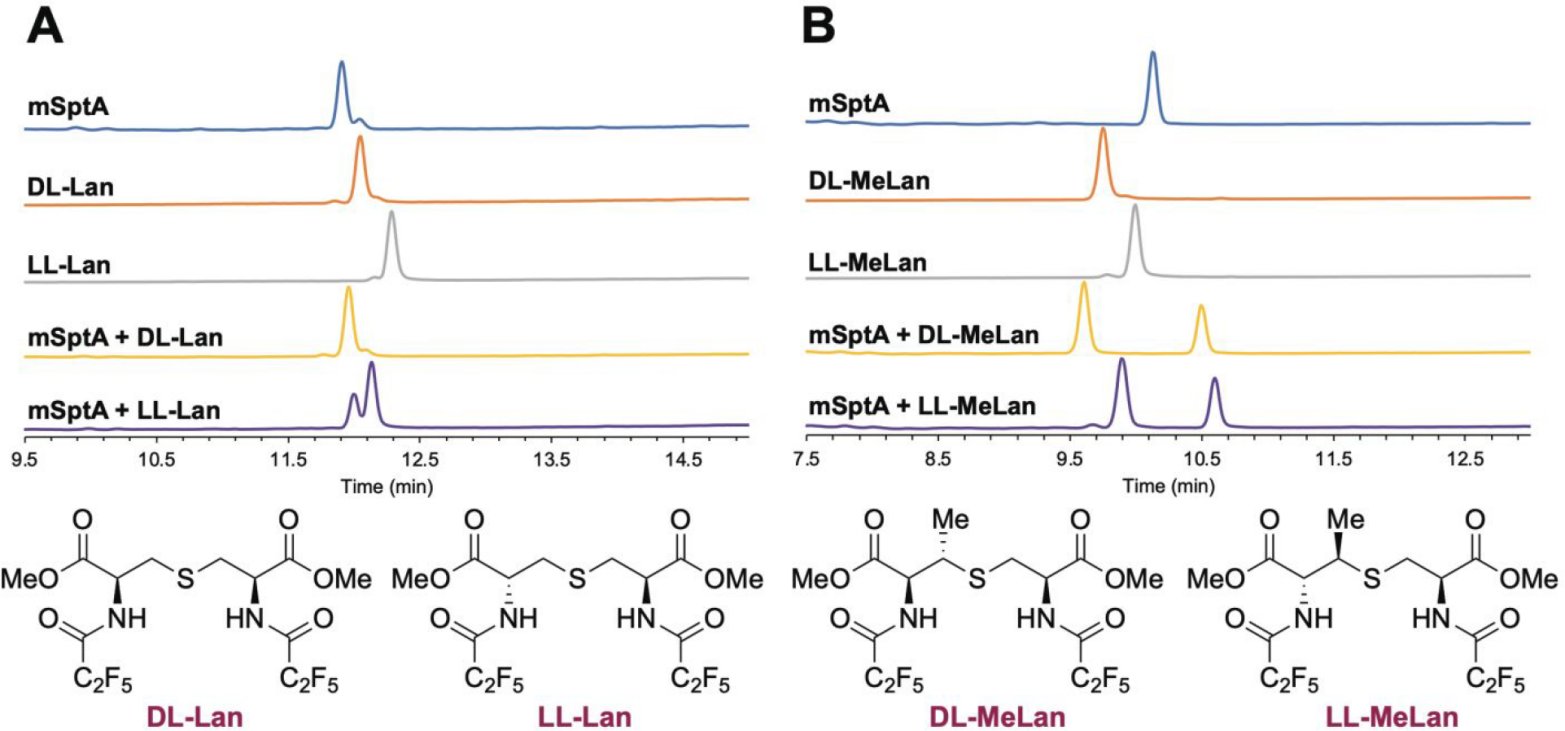
GC-MS analysis using a chiral stationary phase with selective-ion monitoring (SIM) used to detect Lan (*m*/*z* = 365) or MeLan (*m*/*z* = 379). (A) Analysis of the Lan residue in mSptA and comparison to dl- and ll-Lan authentic standards. (B) Analysis of the MeLan residues in mSptA and comparison to dl- and ll-MeLan authentic standards. Spiked samples are indicated in the GC-MS traces, and the structures of the authentic standards in derivatized form for GC-MS are shown at the bottom.

GC-MS analysis of the hydrolyzed and derivatized mSptA sample revealed roughly a 3:1 ratio of MeLan:Lan, consistent with SapT isolated from its native producer.^16^ Co-injections with stereochemically pure dl-Lan and ll-Lan prepared as reported previously^36^ allowed assignment of the Lan in mSptA to the dl-configuration (Figure 4A). A single peak was observed for derivatized MeLan; however, coinjections with stereochemically pure derivatized dl- and ll-MeLan standards surprisingly showed these isomers did not coelute (Figure 4B). Epimerization during the hydrolysis process could not account for this result because the hydrolysis was performed in DCl/D_2_O and a mass shift should be observed for epimerized (Me)Lan amino acids.

### Synthesis of Methyllanthionine Standards

The most likely explanation for the GC-MS data is that the observed MeLan isomers correspond to either (2*S*,3*R*,6*R*) or (2*R*,3*S*,6*R*)-MeLan (d-*allo*-l-MeLan and l-*allo*-l-MeLan, respectively). The stereoselective synthesis of MeLan stereoisomers has been outlined in various reports,^5,36−39^ but has almost exclusively focused on dl- and ll-MeLan. We envisioned that we would be able to determine the stereochemistry of the MeLan in mSptA using a different approach.

**Figure 5:**
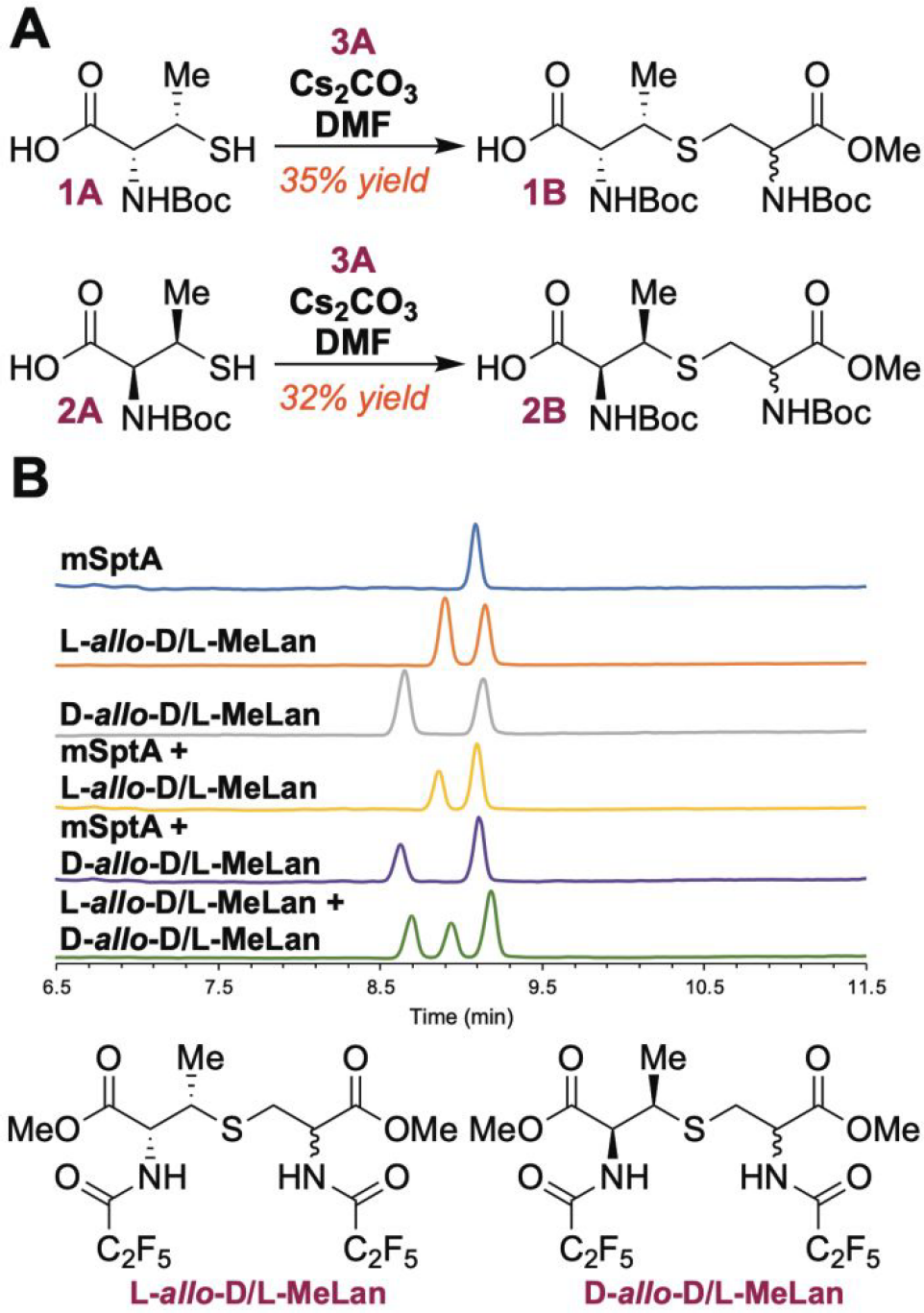
(A) Synthesis of l-*allo*-d/l-MeLan (**1B**) and d-*allo*-d/l-MeLan (**2B**). Diastereomeric ratios were determined by GC-MS. (B) MS analysis using a chiral stationary phase with selective-ion monitoring (SIM) to detect MeLan (*m*/*z* = 379) in mSptA and comparison to d- and l-*allo*-d/l-MeLan. Spiked samples are indicated in the GC-MS traces, and the structures of the authentic standards in derivatized form for GC-MS are shown at the bottom.

We first synthesized mixtures of d-*allo*-d/l-MeLan and l-*allo*-d/l-MeLan by Michael-type addition of l-*allo*-thiothreonine (**1A**) or d-*allo*-thiothreonine (**2A**) to protected dehydroalanine (**3A**) (Figure 5A). The stereoselective synthesis of *N*-Boc-l-*allo*-thiothreonine (**1A**) has been previously accomplished through a ring opening reaction of a Boc-l-Thr-OH derived lactone with potassium thioacetate followed by subsequent hydrolysis.^40^ Thus, both enantiomers of *N*-Boc-*allo*-thiothreonine were synthesized in three steps starting from commercially available Boc-l-Thr-OH and Boc-d-Thr-OH (Figures S4 and S5). Next, *N*-Boc-Dha-OMe (**3A**) was synthesized through the elimination of Boc-l-Ser-OMe (Figure S6).^41^ Addition of thiol **1A** or **2A** to dehydroalanine **3A** in the presence of Cs_2_CO_3_ in DMF generated l-*allo*-d/l-MeLan (**1B**) and d-*allo*-d/l-MeLan (**2B**) in which the amines were Boc protected and one of the carboxylic acids is protected with a methyl ester (Figure 5A).

### Elucidation of the MeLan Stereochemical Configuration in mSptA

l-*allo*-d/l-MeLan (**1B**) and d-*allo*-d/l-MeLan (**2B**) were derivatized for GC-MS as described above for mSptA except that the *N*-Boc groups were first removed with trifluoroacetic acid (Figure S9). We analyzed l-*allo*-d/l-MeLan and d-*allo*-d/l-MeLan by GC-MS with a chiral stationary phase to first determine whether the derivatized MeLan of mSptA coeluted with a peak in either l-*allo*-d/l-MeLan or d-*allo*-d/l-MeLan (Figure 5B). Comparison of retention times and coinjection experiments confirmed that mSptA indeed contains a novel MeLan diastereomer. Unfortunately, the derivatized MeLan from mSptA coeluted with a peak common to both synthetic samples under all experimental conditions tried (Figure 5B).

**Figure 6:**
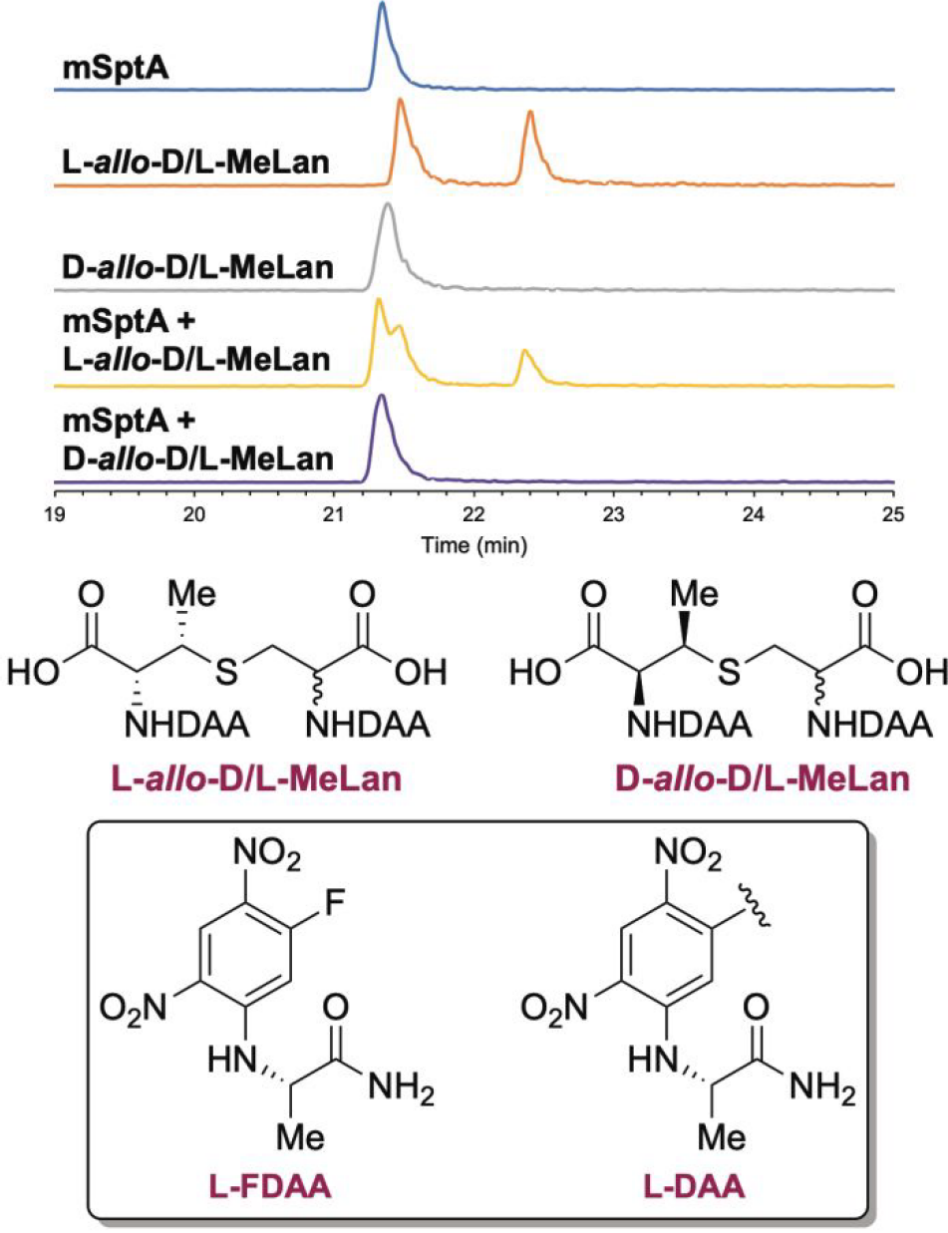
Derivatization of l-*allo*-d/l-MeLan, d-*allo*-d/l-MeLan, and the mSptA hydrolysate with l-FDAA followed by LC-MS analysis. EIC monitored for bisderivatized MeLan (*m*/*z* = 727.1742). The structures of the derivatized standards are shown at the bottom of the figure.

We next used a chiral derivatizing agent. Marfey’s reagent, *N*_*α*_-(2,4-dinitro-5-fluorophenyl)l-alaninamide) (l-FDAA), reacts with amines and has been used for differentiating between l- and d-amino acids,^42^ as well as for detection of (Me)Lan by liquid-chromatography mass spectrometry (LC-MS).^14,43^l-*allo*-d/l-MeLan and d-*allo*-d/l-MeLan were derivatized with l-FDAA (Figure S10), and advanced Marfey’s analysis^42^ was used. Extracted ion chromatograms (EICs) were monitored for the bisderivatized products, as MeLan contains two amines and both would be expected to be modified. Two peaks were detected for l-*allo*-d/l-MeLan and one broad peak was detected for d-*allo*-d/l-MeLan, suggesting that d-*allo*-d-MeLan and d-*allo*-l-MeLan were inseparable under the experimental conditions (Figure 6).

mSptA produced in *E. coli* was hydrolyzed, and the hydrolysate was derivatized with l-FDAA. Comparison of retention times and coinjection experiments ruled out both l-*allo*-d-MeLan and l-*allo*-l-MeLan as possible candidates for the MeLan in mSptA (Figure 6). Instead, the derivatized MeLan from mSptA coeluted with d-*allo*-d/l-MeLan confirming that mSptA contains either d-*allo*-d-MeLan and/or d-*allo*-l-MeLan. Since the precursor peptide SptA is ribosomally synthesized and thus contains l-Cys, it is most plausible that mSptA contains d-*allo*-l-MeLan (Figure 1). Nonetheless, we sought to unambiguously support this conclusion experimentally.

**Figure 7:**
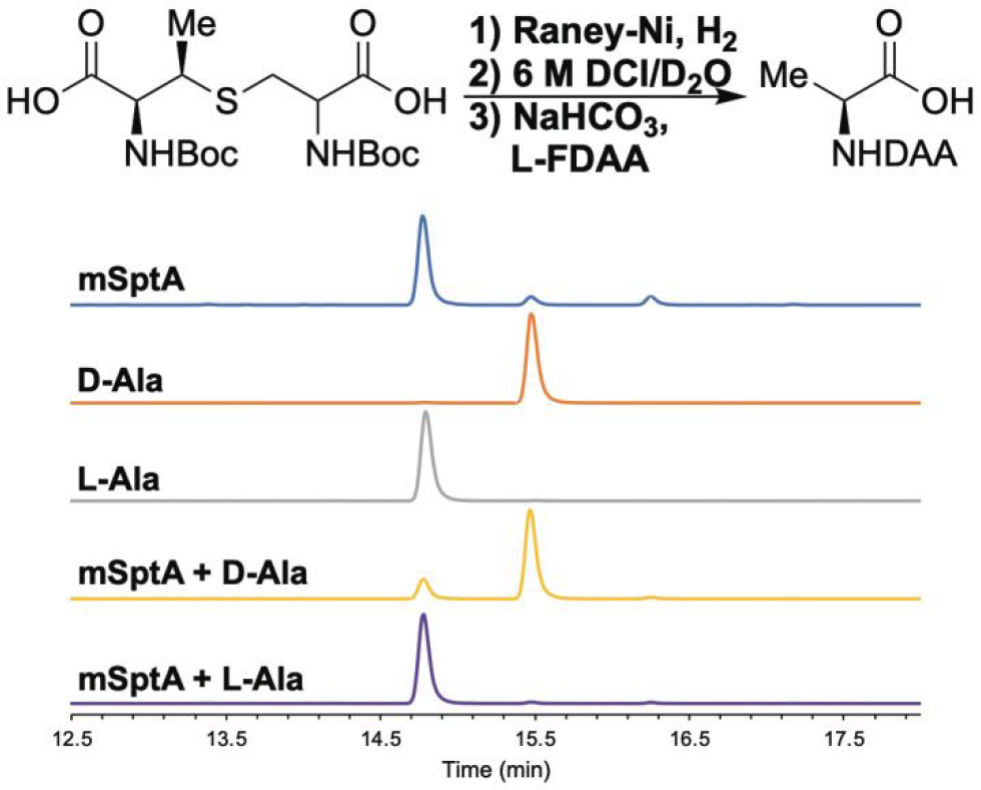
Reductive desulfurization of (Boc)_2_-MeLan from mSptA hydrolysate, followed by Boc deprotection, and l-FDAA derivatization. Top: reaction conditions for modification of (Boc)_2_-MeLan. Bottom: comparison of product after l-FDAA derivatization to authentic l-Ala-FDAA and d-Ala-FDAA. EIC monitored for derivatized Ala (*m*/*z* = 342.1044).

To confirm that mSptA contains d-*allo*-l-MeLan and not d-*allo*-d-MeLan would require demonstration that the compound has the (6*R*)-configuration. One way to achieve this would be to convert MeLan into Ala and aminobutyric acid through reductive desulfurization,^14,44,45^ and verification that the Ala has the l-configuration (Figure 7). Accordingly, the mSptA hydrolysate was reacted with Boc_2_O and (Boc)_2_-MeLan was isolated by LC.^14^ (Boc)_2_-MeLan was then treated with Raney nickel, followed by Boc removal, and derivatization with l-FDAA.^14^ Analysis of the derivatized Ala residues and comparison to authentic l-Ala-DAA and d-Ala-DAA confirmed the presence of l-Ala-DAA (Figure 7). The reaction product of reductive desulfurization therefore contains l-Ala, and thus mSptA must contain d-*allo*-l-MeLan residues.

### SptB_b_ is a Member of a Glutamyl Lyase Family that is Divergent from Other LanB Enzymes

The unexpected stereochemistry observed for the MeLan in SapT prompted the bioinformatic analysis of the SptB_b_ and SptC enzymes. The former catalyzes the elimination of glutamate from glutamylated Ser/Thr and the latter the Michael-type addition of Cys to Dha/Dhb. For Thr/Dhb these enzymes will set the stereochemistry of the MeLan product. Sequence alignment of SptC with other LanC enzymes and structure prediction using trRosetta^46^ did not show any particularly notable differences. SptC contains a His residue (His191) that has been shown to be the catalytic acid that protonates the enolate formed during the Michael-type addition in other LanC enzymes/LanC-domain containing enzymes (Figure S11).^47,48^ The protein also contains all ligands for Zn^2+^ binding,^49^ and the predicted structure positions the Zn^2+^ required to activate Cys and the acid that protonates the enolate in very similar juxtapositions. Hence, SptC likely catalyzes the same *anti*-addition from the *Si*-face of the dehydroamino acid, which is also supported by the stereochemistry of the Lan in Figure 4A.

**Figure 8:**
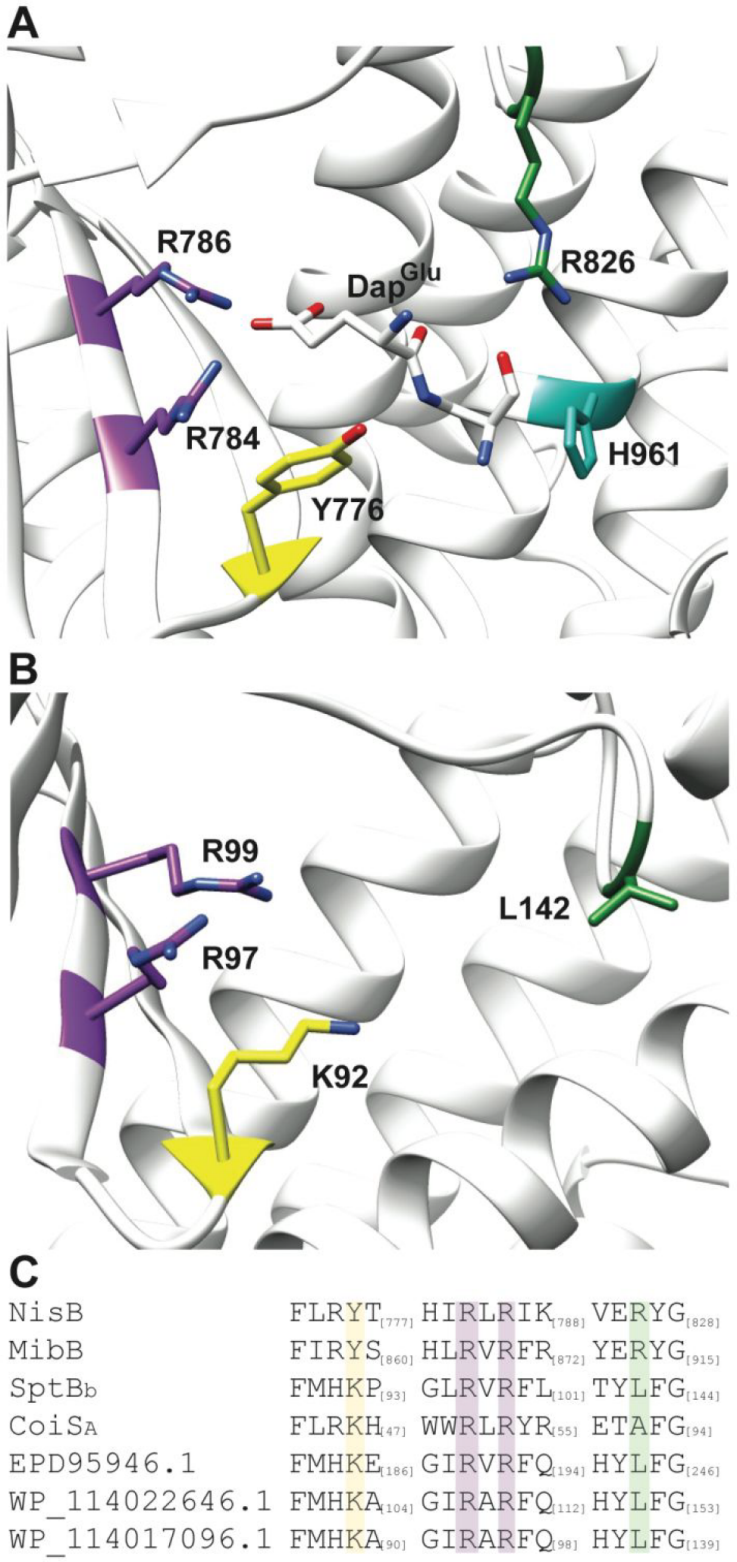
Comparison of the nisin dehydratase NisB with SptB_b_ and SptB_b_ homologues. (A) Co-crystal structure of NisB and a glutamylated peptide analog Dap^Glu^ (PDB: 6M7Y). (B) Structure of SptB_b_ calculated with trRosetta. (C) Sequence alignment of SptB_b,_ SptB_b_ homologues, CoiS_A_, NisB, and MibB. An expanded alignment is shown in Figure S12.

We then aligned the sequence and structure of SptB_b_ with other elimination enzymes or elimination domains in full length LanB proteins (Figures 8 and S12). A recent cocrystal structure of the nisin dehydratase NisB bound to a synthetic substrate analog in which the ester linkage between Ser and glutamate was replaced by an amide provided insight into the residues that are important for substrate recognition and catalysis (Figure 8A).^26^ Two Arg residues that interact with the carboxylate side chain of the glutamyl group are conserved in NisB and SptB_b_ and are situated deep into the pocket in both the crystal structure of NisB and a trRosetta model of SptB_b_ (Figure 8A and B). However, the catalytic His that deprotonates the α-proton of the glutamylated Ser/Thr in NisB is missing in the alignment for SptB_b_ and the predicted structure of SptB_b_, as the equivalent sequences are divergent and do not align. Furthermore, SptB_b_ contains a Leu at position 142 (Figure 8B) instead of the Arg residue present at the corresponding position in NisB that acidifies the α-proton of the glutamylated Ser/Thr by interaction with the backbone carbonyl of this residue. Prior experiments in glutamyl lyases have demonstrated that replacement of these His or Arg residues resulted in abolished or severely reduced lyase activity.^26,27^ Thus, the bioinformatic analysis suggests that SptB_b_ utilizes the same mechanism for recognition of the γ-carboxylate of the glutamyl adduct, but a different mechanism for the elimination reaction.

SptB_b_ and related homologues contain a fully conserved Lys residue at a position where NisB contains a Tyr residue (Figures 8 and S12). Since SptB_b_ lacks the active site His base, this Lys may be important for deprotonation of the glutamylated Ser/Thr during SptB_b_ catalysis. It is tempting to speculate that the elimination reaction in SptB_b_ occurs with *syn* stereochemistry to generate (*E*)-Dhb, which upon canonical *anti-*addition by SptC from the *Si-*face would furnish the observed d-*allo*-l-MeLan. Confirmation of this hypothesis will require *in vitro* reconstitution of the glutamyl-tRNA dependent dehydration, which at present has not been achieved because we have been unable to obtain the unmodified precursor peptide SptA; without the cyclization of the core peptide, the precursor appears to be sensitive to proteolytic degradation during expression in *E. coli*. Use of a K92A variant of SptB_b_ in the heterologous production system did not yield any peptide product either, implying that core peptide cyclization was not accomplished and, hence, that the unmodified SptA was again proteolytically degraded. These findings indirectly support the importance of the Lys for successful modification of SptA.

### The Presence of the Divergent Glutamyl Lyase is Predictive of d-*allo*-l-MeLan Formation

Next, we generated a sequence similarity network (SSN) using the tools of the Enzyme Function Initiative (EFI)^50,51^ with the elimination domain Pfam PF14028 (Lant_dehydr_C) as query. Inspection of the genomic context of the glutamate lyases revealed a large group of lanthipeptide synthetases that have the glutamylation and elimination domains in a single polypeptide (Figure S13, black). SptB_b_ is in a separate sizable group of elimination enzymes of split LanBs (Figure S13, blue) that all have the same constellation of active site residues suggesting they all may be involved in formation of d-*allo*-l-MeLan. Another relatively large group of 426 putative elimination domains/proteins also separate from the full length class I dehydratases (Figure S13, purple). The lanthipeptide BGCs containing these enzymes in almost all cases contain *two* annotated elimination domains and are all found in actinobacteria (e.g., Figure S14). We wondered whether these BGCs might utilize two separate elimination domains to generate (Me)Lan residues of different stereochemistry within the same product.

We tested this hypothesis with a representative member from this group from *S. coelicolor* A3(2) that we termed the *coi* BGC (Figure S14; NCBI accession WP_011031310.1). This BGC is related to the previously reported *olv* BGC from *Streptomyces olivaceus* NRRL B-3009 (NCBI accession WP_031034767.1), but contains an additional elimination domain that is fused to the methyltransferase CoiS_A_. The methyltransferase domain of CoiS_A_ in turn has sequence homology to OlvS_A_ that converts Asp to isoAsp (Figure S14).^7^ The CoiS_A_ elimination domain (CoiS_A(ED)_) and SptB_b_ feature similar putative active site residues based on sequence alignments (Figures 8C and S12). In addition, the *coi* BGC also encodes a full length LanB dehydratase CoiB that contains a canonical glutamyl lyase domain.

To investigate if the *coi* product contains d-*allo*-l-MeLan, the genes for one of the three encoded substrate peptides (CoiA1), CoiB, the cyclase CoiC, and CoiS_A(ED)_ were cloned into expression vectors, and CoiA1 was coexpressed with these enzymes in *E. coli*. Isolation and analysis of modified CoiA1 (mCoiA1) by MALDI-TOF MS demonstrated a threefold dehydrated product (Figure S15). Hydrolysis and derivatization of mCoiA1 followed by GC-MS analysis showed two peaks corresponding to dl- and ll-MeLan consistent with previous observations for the *olv* cluster,^7^ but a third peak eluted later that was not observed for the *olv* BGC (Figure S16). Hydrolysis of mCoiA1 and derivatization with Marfey’s reagent followed by LC-MS analysis as described above for mSptA confirmed the presence of d-*allo*-d/l-MeLan (Figure S17). These data strongly support the bioinformatic prediction that the SptB_b_-like elimination domain that is fused to the methyltransferase plays a critical role in the formation of d-*allo*-d/l-MeLan.

Finally, we returned to the question whether the highly conserved Lys residue found in SptB_b_, CoiS_A(ED)_, and their homologues is critical for enzymatic activity. The variant CoiS_A(ED)_-K46A was coexpressed with CoiA1, CoiB, and CoiC in *E. coli* and the resulting product peptide purified by Ni-affinity chromatography. Analysis by MALDI-TOF MS demonstrated the accumulation of glutamylated intermediates (Figure S15), suggesting that the highly conserved Lys found in these noncanonical glutamyl lyases is important for glutamate elimination.

### Genome Mining for Morphogenetic Lanthipeptides

**Figure 9:**
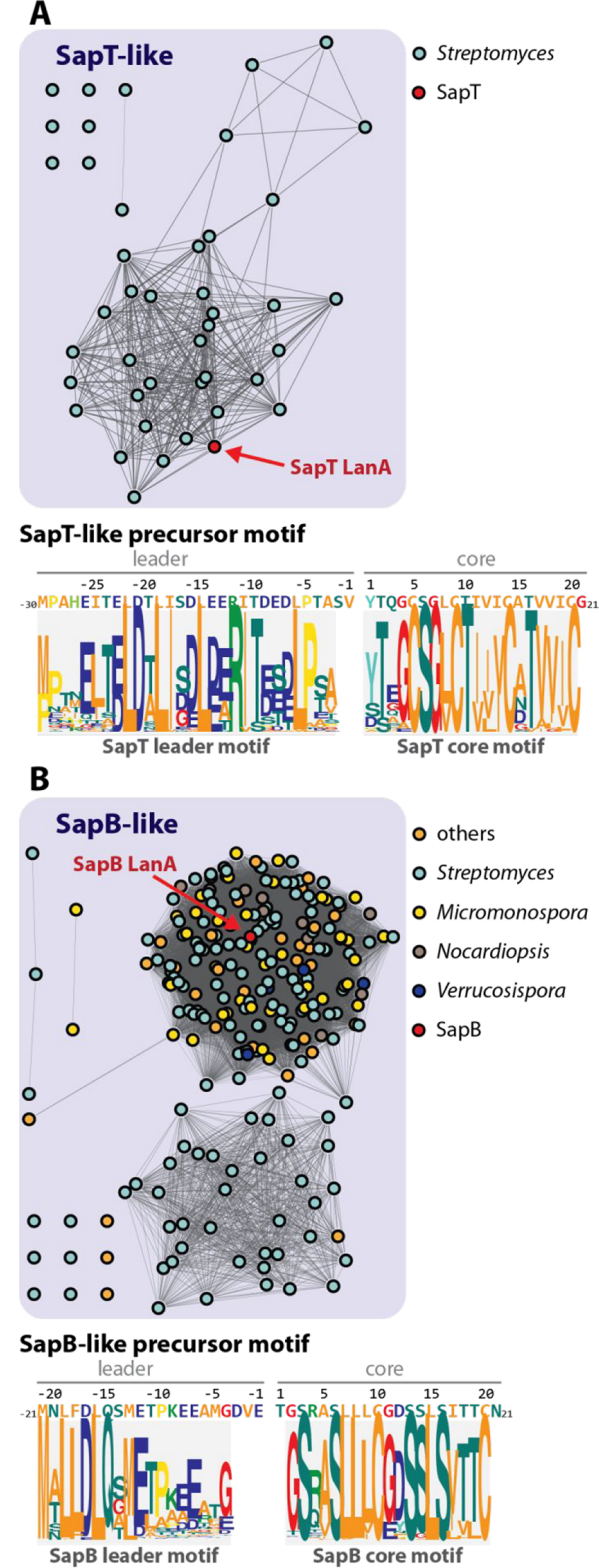
Genome mining for morphogenetic lanthipeptides. Shown are SSNs and conserved sequence motifs for homologues of (A) SapT and (B) SapB. The SSNs were generated based on the core peptide sequences with an alignment score threshold of 5. Every node (circles) in the SSNs corresponds to one unique precursor sequence and is colored according to the genus of the producing organism. Below the SSNs, the SapT/SapB precursor sequences are shown in comparison to conserved sequence motifs in the identified homologues.

We also used the identification of the SapT BGC in this study to search genomic databases for related BGCs in other organisms. Using the SptA sequence as an input, we identified a number of homologous BGCs (Table S8). Of the 41 unique precursor sequences identified, six contain an additional fifth S-G-V/I/L/F-V/I/G/F-C sequence at their N-terminus, which will likely result in another Lan moiety in the corresponding lanthipeptides. Interestingly, the homologous BGCs were exclusively found in *Streptomyces* strains, while a likewise search with the SapB precursor as query also yielded hits in other bacteria forming aerial hyphae like *Nocardiopsis*, *Verrucosispora*, *Kitasatospora*, or *Micromonospora* species (Figure 9; Table S8). To provide a better overview of the genome mining results, we generated an SSN based on the core peptide sequences of the identified precursor homologues (Figure 9). Furthermore, the MEME algorithm^52^ was employed to identify and visualize conserved sequence motifs in the leader and core regions present in SapT- and SapB-related precursors (Figure 9). As expected based on the morphogenetic functions of SapT and SapB, a high conservation of the hydrophobic residues in the core regions was observed. In addition, the leader motifs show highly conserved regions that are likely important for enzymatic recognition.

## Discussion

Heterologously produced SapT is the first lanthipeptide characterized to contain d-*allo*-l-MeLan. Lanthipeptides were at one time assumed to only contain MeLan with the dl-configuration,^4,9,10,13^ formed by *anti-*addition to the *Si-*face of Dhb. Studies in the past decade have demonstrated that class II lanthipeptides sometimes contain MeLan with the ll configuration,^4,9,10,13^ formed by *anti-*addition to the *Re* face of Dhb. The formation of ll-MeLan residues was initially shown to be guided by a conserved Dhb-Dhx-Xxx-Xxx-Cys motif (where Dhx represents Dha/Dhb and Xxx represents any amino acid except Ser, Thr, Cys) that results in a substrate-controlled stereoselective cyclization.^9^ More recent additional examples of the ll-(Me)Lan stereochemistry were identified in class I lanthipeptides where the conserved Dhb-Dhx-Xxx-Xxx-Cys motif was absent suggesting that *anti-*addition to the *Re*-face is more common than previously thought.^7,8^

**Figure 10:**
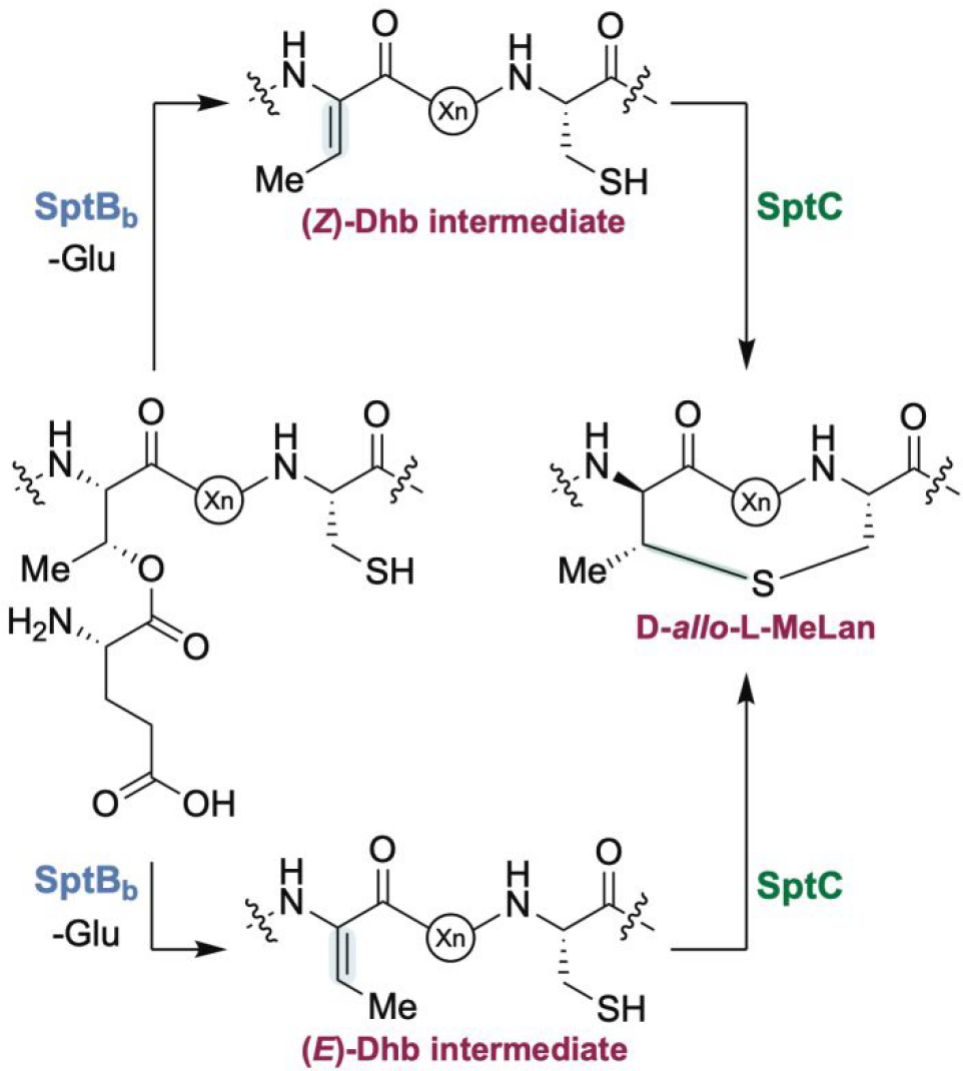
Two possible reaction pathways for the formation of d-*allo*-l-MeLan.

The unanticipated stereochemistry of the MeLan residues in SapT raises new mechanistic questions for stereochemical control during lanthipeptide maturation. Canonical methyllanthionine formation is proposed to occur through an *anti*-elimination of activated Ser/Thr residues followed by the *anti*-addition of l-Cys residues across the corresponding dehydroamino acid.^26,28,48,49^ This sequence of reactions cannot account for d-*allo*-l-MeLan formation from l-Thr. Two distinct mechanisms can be envisioned that may explain the experimental observations (Figure 10). One possibility is that the SptC cyclase catalyzes a *syn*-addition of l-Cys to the *Re* face of a (*Z*)-Dhb residue. However, the SptC cyclase appears to be very similar to characterized LanC cyclases that catalyze *anti-*additions. Hence, we favor an alternative model in which the stereochemistry is controlled at an earlier stage. We suggest that SptB_b_ and CoiS_A(ED)_ catalyze glutamate elimination with *syn*-stereochemistry to generate an (*E*)-Dhb intermediate (Figure 10). This model would then require an *anti*-addition of l-Cys from the *Re* face of the (*E*)-Dhb intermediate to form d-*allo*-l-MeLan. We note that *anti*-addition of l-Cys from the *Si* face of the putative (*E*)-Dhb intermediate could also form l-*allo*-l-MeLan, which to date has not been reported. Thus, depending on the LanC cyclase a fourth diastereomer of MeLan may be present in lanthipeptides. (*E*)-Dhb residues have been detected for nonribosomal peptides such as albopeptide,^53^ but not yet for any RiPP. The differences observed bioinformatically between SptB_b_ and CoiS_A(ED)_ (and other similar enzymes) and canonical class I lanthipeptide dehydratases appear to support a different elimination mechanism. Current investigations are underway to test this hypothesis.

## Conclusion

We report the classification and reconstitution of the biosynthetic pathway of the morphogenetic class I lanthipeptide SapT in *E. coli*. Heterologous production of mSptA resulted in the discovery of a novel d-*allo*-l-MeLan diastereomer in a lanthipeptide. The previously identified mechanism for lanthipeptide maturation is ruled out based on this product stereochemistry. Two mechanistic possibilities are proposed for the formation of d-*allo*-l-MeLan, but the observed divergence in the active site residues of the glutamate lyase SptB_b_ compared to other characterized homologues suggests that this stereoisomer is formed by *syn*-elimination from glutamylated Thr to form (*E*)-Dhb followed by canonical *anti-*addition of Cys catalyzed by SptC. The constellation of amino acids in the lyase active site is predictive of the stereochemistry of the MeLan and lanthipeptides containing the d/l-*allo*-l-MeLan diastereomer are expected to be widespread. This study therefore expands the stereochemical diversity of lanthipeptides and provides a validated bioinformatic model that can predict the formation of d/l-*allo*-l-MeLan diastereomers in lanthipeptides.

## Supporting Information Available

Experimental procedures, supporting figures and tables (PDF)
