# Supplementary material for: Unexpected Methyllanthionine Stereochemistry in the Morphogenetic Lanthipeptide SapT
Source: J Am Chem Soc. 2022 Mar 30;144(14):6373–82. doi: 10.1021/jacs.2c00517 (PMC9011353; doi:10.1021/jacs.2c00517)
Supplement: Supplementary file 1 — ja2c00517_si_001.pdf [file ja2c00517_si_001.pdf]

Supporting Information

Unexpected Methyllanthionine Stereochemistry in the Morphogenetic Lanthipeptide SapT

Raymond Sarksian,[1,§] Julian D. Hegemann,[2,§] Max A. Simon,[1] Jeella Z. Acedo,[1]
and Wilfred A. van der Donk[1*]

[1] Department of Chemistry and Howard Hughes Medical Institute, University of Illinois at Urbana-Champaign, Urbana, IL, 61822, USA
[2] Helmholtz Institute for Pharmaceutical Research Saarland (HIPS), Helmholtz Centre for Infection Research (HZI), Saarland University Campus, 66123 Saarbrücken, Germany.
[3] Department of Bioengineering and Carl R. Woese Institute for Genomic Biology, University of Illinois at Urbana-Champaign, Urbana, IL, 61822, USA
[*] vddonk@illinois.edu

[§] These authors contributed equally to this study

Table of Contents

General methods……S1
Genome sequencing, ORF annotation, and identification of the Spt BGC……S2
DNA sequences……S3
Cloning of the expression plasmids……S4
Comparison of tRNA^{Glu} sequences……S5
HPLC-HRMS conditions……S6
GC-MS conditions……S6
Large scale mSptA co-expression and purification……S6
mSptA trypsin digestion and tandem MS analysis……S9
Hydrolysis and derivatization of mSptA for GC-MS analysis……S10
Small molecule synthesis……S11
Derivatization procedures for GC-MS……S15
Derivatization procedures for LC-MS……S16
Bioinformatic analysis for SptB$_b$ and SptC……S17
Glutamyl lyase SSN……S20
Coi BGC……S20
Coi BGC DNA sequences and molecular cloning……S21
mCoiA1 heterologous production and analysis……S24
Genome Mining Results……S26
NMR spectroscopy data……S34
References……S42

General methods

E. coli DH10B cells were used for cloning, *E. coli* BL21(DE3) for expression. Freeze-dried *Streptomyces lavenduligriseus* Tü901 (ATCC# 31160, listed in the catalog as *S. tendae* Tü901; see

below for the reclassification) cells were purchased from the ATCC and reactivated as instructed. Spore stocks were prepared for long-term storage using standard protocols. The oligonucleotide primers used for cloning were ordered from Integrated DNA Technologies (IDT). Phusion DNA polymerase and Gibson assembly master mix were bought from New England Biolabs, KAPA HiFi DNA polymerase from Roche, and lysozyme, benzonase (25−29 U/μL), and Millipore C18 Ziptips from Thermo Fisher Scientific. The sequences of the cloned plasmids were validated through dideoxy sequencing (carried out by ACGT, Inc.). His_6-TEV protease was obtained by expression from the pRK793 plasmid in *E. coli* BL21(DE3) and purified via NiNTA affinity chromatography according to the protocol published by Waugh and co-workers.[1]

MALDI-TOF-MS measurements were performed on a Bruker UltrafleXtreme MALDI TOF/TOF mass spectrometer. High Performance Liquid Chromatography (HPLC) was performed on an Agilent Infinity 1260 HPLC system (Agilent Technologies, Santa Clara, CA, USA) equipped with a diode array detector and analytical fraction collector. LC columns were held at 55 °C with mobile phases A (H_2O with 0.1% formic acid) and B (acetonitrile with 0.1% formic acid) equilibrated with starting conditions 0-2% B (*vide infra*). High-resolution mass spectrometry (HRMS) was performed in positive detection mode on an Agilent 6545 QTOF MS equipped with an Agilent Dual Jet Stream electrospray ion source with a drying gas temperature of 320 °C and gas flow of 13 L/min, sheath gas temperature of 350 °C and gas flow of 11 L/min. Mass spectra were recorded at 30 eV for targeted MS/MS, and 30, 40, and 50 eV as collision energies for Auto MS/MS acquisitions as centroid data. Purine and HP-0921 (Agilent Technologies, Santa Clara, CA, USA) were used as lock masses during acquisition. Spectra were processed using the Agilent MassHunter Workstation Qualitative Analysis software (version 10.0). Additional information specific to each sample and method is provided in the respective sections.

All chemicals used in small molecule synthesis and derivatization procedures were purchased from either Millipore Sigma or Chem-Impex Intl. and used without further purification. Thin-layer chromatography (TLC) was conducted on Merck silica gel 60 F254 aluminum plates. Potassium permanganate solution or iodine was used for visualization of the TLC plates. Silica gel (60 Å pore size, 230-400 mesh particle size, 40-63 µm particle size) was used for purification of synthetic compounds via flash chromatography. Automated flash chromatography purification of compounds was performed on a Teledyne Isco CombiFlash RF Plus system.

^{1}H NMR spectra were recorded at 500 MHz and ^{13}C NMR spectra were recorded at 126 MHz on a Bruker 500 MHz spectrometer. NMR spectra were referenced to residual NMR solvent signals: chloroform (7.26 ppm, ^{1}H; 77.0 ppm, ^{13}C), acetonitrile (1.94 ppm, ^{1}H; 118.3 ppm, ^{13}C), dimethylsulfoxide (2.50 ppm, ^{1}H; 39.5 ppm, ^{13}C). Chemical shifts are reported in parts per million (ppm) and multiplicities are indicated for observed splitting. Coupling constants are indicated in Hertz. Mass spectrometry using electrospray ionization via TOF analyzer for synthesized small molecules was performed by the University of Illinois Mass Spectrometry Laboratory.

Genome sequencing, ORF annotation, and identification of the *spt* Operon

GYM medium (100 mL of 4 g/L glucose, 4 g/L yeast extract, 10 g/L malt extract, pH 7.2) in a 250-mL baffled flask was inoculated with 10 μL of an *S. lavenduligriseus* Tü901 spore stock suspension and the cells were shaken at 30 °C for 10 days. Then, 4 mL of cells were harvested by centrifugation and used for genomic DNA (gDNA) isolation employing an UltraClean Microbial DNA Isolation kit (MoBio Laboratories) following the manufacturers' protocol.

Genome sequencing was performed on an Illumina HiSeq 4000 in the DNA Services Lab of the Roy J. Carver Biotechnology Center at the University of Illinois at Urbana-Champaign, which carried out sample preparation, sequencing, and genome assembly using standard protocols. The assembled genome was deposited in GenBank (BioProject accession number: PRJNA713618 / BioSample accession number: SAMN18255054 / genome accession number: CP072000). Reclassification of *S. tendae* Tü901 to *S. lavenduligriseus* Tü901 was suggested during the deposition of the genome sequence by GenBank, as a routine average nucleotide identity (ANI) analysis[2] based on all trusted type genome assemblies in the GenBank database revealed that the sequenced genome is much more similar to the genome of the type strain of *S. lavenduligriseus* (99% identity with 89% coverage) than to the genome of the type strain of *S. tendae* (84% identity with 39% coverage). Hence, while ATCC 31160 is still listed as *S. tendae* Tü901 on the ATCC website, *S. lavenduligriseus* Tü901 is a more appropriate strain designation and is therefore used throughout the manuscript.

Genome annotation was carried out with the *Rapid Annotation and Subsystem Technology* (RAST) webtool[3-5] using the ClassicRAST annotation scheme and the following settings: preserve gene calls – no, automatically fix errors – yes, fix frameshifts – no, backfill gaps – yes. RAST annotation yielded 8148 annotated features. The antiSMASH 6.0 webtool[6] was used to analyze the genome for the presence of secondary metabolite BGCs using default settings. Thereby, 45 putative natural product BGCs were identified, of which three were predicted as lanthipeptide BGCs. Closer inspection of these three lanthipeptide BGCs allowed the identification of the SapT BGC by matching the C-terminal regions of small ORFs to the core peptide sequence derived from the SapT primary structure.

DNA sequences

DNA sequence of the *spt* operon. The color coding refers to: *sptB$_b$*, *sptB$_a$*, *sptA*, *sptC*, *histidine phosphatase gene*, *sptR*, *sptK*, and the overlap between *sptR*-*sptK* genes:

GTGACATACCTCCCGCGCACTCCTGTTGATCATATGACCAATTCGCTTCCGCGCTTCGGAACACATGCGACGTGGACATCACAGGAGTGGATTGCCCTTCCCGGTG
AGGAGGTCGCATGGTGCGGGTCGAGCGGTTACTGGCCGACTGCCTGGACGACGTACGTGCCGAGCCCCTGGGCACCGTCCCCCTCACGGCGGAGGACCCCGGATAC
GCGGCGGCCCGACGGACGTTTCTGACGGCCGGGCTCGAAGCCCTGCGCGCCCACGCCCCCGAGGCCGGCTGGATCCAGCTCAACCTGGCCGTCACACCGCCACCGT
ACGGCCGACTCGCCACGGCGGCCCGTGAGTTGCTCGGCACCGGACAGGCCCGCGACTTCTTCTTCATGCACAAACCCCCGGGGCTGCGCGTACGGTTCCTCGCGGC
GGAGCCGTCCCGGGCACCCGAACTGCGCGCCGACCTGCTGCGCCGGCTGACGCCGGAGCGGACCGGGGAGGCCGGGACGCCGCCCGTGGCCGGTGTGTACGAGCCG
GAGACCTATCTCTTCGGCGGACCGCGCTCCATGCCCTGGGTGCACGAGCTGTTCACCGCGGACTCCCGGGCCTGGCTGGACGTGCACACCGCCGTGGCCGGCGATC
CGGCTCCGGTCGGCTGGCGGGTTTCCCTGGCCTTGCTGCGCGCCGTGTTCGACGGGCTGGGCATCGTCGGCTGGGAGCACCGGGGGGTCTGGCAGGTGGTACGGGA
GGAGACCGGCCGACGGCTGCCGGGCGGCCTCGGCGAGCCGGACCTGCGCCGGGCCGCCGCCGGGATCCGCGCCTACTGGGAGCAGGGTCCCGAGGACCGGCTCCAG
GCCCTGCCGAAGGCCTGGCGGGACGTCCTCGGCGAGCATCTGGCGGCGGTGGGGCGGGCCGCGGAGCGCTGGCACGGCCACTACTTCGCCTCCGGTGAGGCCACGG
TCGGTCCGCGCCGGGCCGCCGCCCACCACGTGATCTTCCACTGGAACCGGGGCGCGCTCTCCACCGCACGCCAGTGCCTGCTCACCGAGGCACTCGCCGCCGACGG
CCGGGAGGAGGCGGACTGAGCATGGAACACGTACCGGAACAAGCGGCCCAGCCAGCCGACAGTCAGGACAGGCAGGACAGGCACGCGAGCCCCGACACGACACGCG
GAACCACGGCCGAGCCCACGCCCAGCACGGCACCCGGCACCACGGCCGACCCCACGCCCGTACCGGCGCAAGCCACCGAACCGGCCGACACGCGCGACACCCCGCT
CGGGGCCACGGCGCTGCTGCGGCTGGGCGGCATGCCCGGCGCCGCCTGGACGGCCGGCGCCGCCGAGCACCTCTTCGAGGGCGCCGCCCGGCACGCCGACACCGCC
GAGCGGCTCGCCGCGCAGGCCCGGGCGCTGGCCGACCGGCTGGGTGCCGAGGTCGTACCGCACCCTTCGCTGGCGTCCGCCGACCGGGGCGCCGTACTCGCGCTGC
GGCGGCGGCTGCACTCGGGTACGGCACCGGGGGCGCCGGACTGCCTGCTGCTGGAGCGCTCGTCCGCCGTGCCCGCCGACCTCGCCGGGCAGGCCCGCGCCCTGCT
GCGCGGCGCCGAGGCGGCGGAGGCGGCACTGACCCGGCTGCGGCAGGAGGTCACGGCCGAGCAGCAGCGGGTCGCCGAGCAGGCGTGGCGTACGGCGTGCTCCAGC
CCCGTGATGCGCGCGTTCCTGGACAGCGCCGCTCCCGCGCTCGCCGACGACATCGAACGGCGGCTCGCCGACGGCCAGTCGTGGTCCGGCAAGCAGTTGCGCAAAC
GTGCCGCCTATCTGTGGCGCATCGTGGGCCGGGCCGCGGTGAAGACGACACCGCGCGGCTGGGCCGGCCAGATCGCCGCGCTCCCGGTGACCGACGGCCCGGACGA
CGGCTGCCCGCCGCTGCTCGCGCCCGGTACCCCCCTCGGTGCCCTCGCCGCGATCGCCGTGGAGAACGTGCACCTCCTGCGGGCCGGTACCGCCCCCGCGGATCTG
CGCACCGCCGACCCCGCGACCCTGCTCGCGCCGACGCCCCTGCACTTCGCCGAACCGTCCGCCCCCGCTCCCGGCGACCCGCCCGGACAGGTGCGCTGTTACGTCG
TGGACCCGCGTGAACCCGGGCGGCTCCGCCAGATCGTGCTGCGCCGCACCCGCGTGCTGGAAAGCGTCCTGGCCCTGCTCGCCGACGGGCCGCGCACGCTCGGCGA
ACTGGACCGCGTGCTGCCGTCGCTGAGCGCCCCGCCCGGCCGGCCGGCCGTCGCCCCCGCGGTACTGCGCGGGTTCCTCCAGCACCTGCACGGGCTGGGCGTCGTA
CAGATCTGCCGGGCGCCCCGGCAGCACTGCTCCGGCTGGGTGCCGGCCGAGACGGTCGGCGCCACCGGCGCGCTGCCGCCGACGCCCGCGGGGGGCGGGGCGGACG
CCTGGTTCCTCGACTCCTACCGCAGGCTCGGCGCCGACGCCGCCGTGCCCGCGGCAGCCGCCGCACGCGTCCGGCGCGGGCTGCGGATCGCGGCCCGGCTCGCCGC
ACTACGGGAGGCCGACCGGCCGGCGGAGCCGCCGGAGCGACAGCCCTGGTTCGCCGGGCTCACCGAACAGGCGCGCCCCATCAGCGAGATCCTCGCCGACCGGCTG
AGCGCGGACGAACCGCCGCCCGTCCGCCGCTACACGGGCTGGCACCCCGCCCACGACCCCGCCGGCGGTTACGCCCGGCTGCTGGCCCACCTGGACGCCGCCGCCC

GCTCCGGCACGGCCTGCGTGGACGTGGACGACGCGCTGCTGGACGAGCTGGGCGCACCACCCGGCGGTGCCACGCTGCCGCCCTGGCCGGTCGACTGTCTGCTGCG
CCCGCTGCCCCCCTGCGCCGACGGCGGCCCGGTGGCCGTGCTGGAGACCGCCTCGTCCGCCGGGGTGCTCGACGCCCGGTTCGCCGACGGACTGCGCACCCTGCAC
GGCGGCTACGGCAACAGCGACGCCTACCGCGCGTTCCTCGCCGCCGTCGAGGAGCGCACCGGGGTCCGCTTCGTCGACCTGCTCGTGCCGCCGCTCGCCGAGCGGT
CCGCCAACGCCGTGCGCCGTCCGGTGACGACCCGCTGGTGGACCGGCGACCCGGACCCCACGCCCTACTACGGTTCGCCCGGCGGGCACGCGCGCTACCTCCCGCT
GGACCGCATCAGCCTGCGGCGCGCCCACGGCCGGATCGTCGCCGAGGCGGACGGGCGGCGCGTCGTCCCCGTGTACCAGGCCACCCGCTCCCCCGCCCCGCCCTAC
GACACCCTCGTACGGCTGCTGCTCGCGGCGAGCCATCCGGCGGCCTCCTATCTGGTGCGGCTGGACGCGCTGGACGCGGCGCTGCCCGACCGGGCCCGGCTGCCCC
GGCTCACCGCGGGCGGCGACCTCGTCCTCGCCCCGGCGACCTGGCGCGTCGACCGCGGCAAGCTGTGGGAGCCCGGTGACGACCTGCTCACCAAGGTCCGGTCCCT
CGCCCTGCTGCGCCGGTCCGCCGGGCTGCCCCGGCACGCGTTCGTGCGCACCGCCCCCGGCGCGAAGCCGGTCCCGGTGGACCTCGCCTCGCTGACGGCCCTCCAG
CTGATCGAGCGGCTGTGCGCCCGGGAGGCGGGCACCGCGCTGCTGGTGGAGGAGATGCTGCCGGCCCCCGAAGACCTGCTGTTGCACGACCCGCTGCACGGCGGCG
CGAGCGTCGCCGCGCAGCTCCTGCTCCGGCTCCCCCACGACCGATGCGCCGGCCGGCTCGCCGAGGCCGCCGCCGACGCCTTGGTCCACGGTGGCCGGCACGACGT
CCCCGGCCCGCCCGCCAGGCGGCCCGCCGGGGCGGCCAACACCCGATGAGTGAAAGGGGAAGTCCCATGCCCGCACACGAAATCACCGAACTCGACACCCTGATCA
GCGACCTCGAGGAGCGGATCACCGACGAGGACCTGCCGACGGCATCCGTGTACACCCAGGGATGCAGCGGCCTGTGCACGATCGTCATCTGTGCCACGGTCGTCAT
CTGTGGATGACGCTCCCAGGAGCCGCCCGCCCGTGCGGGCGGCTCCGCCCTGTCCGCGACGACGTTTGGAGGACGATGCCGCACGACACGGCCGTACCGGCCGCCC
CGCCGGACGAGGACGTCCACGGGCTCGCCGTCCGCTACCTGCTGGAGTGGGCCGAGGCGCCCGAGCGGGGCGGACACCGCTGCGACCCCGGTATCCCGGTGCTGGC
CCACCTGGTCGCGGACGACGAGGCGGCGCCCGCCGCCGTGGCCGTGTGGTCCCGCACGGCCGGCCGCGGGCCGTCCCACCCCGCCCTGTACGACGGGGGCCTGGCC
GGCACGCTCGTCGGCCTGCGCCTGGGCGCCCGGGTGCACCCCGGGCTGGGGGCCGCGGCGGACCGGCTCGGCGCCCACCTCGCCGGGCGCCCGCCCGGGTACCGCA
CCCGCGAGGTCGCGTTTCCCGACTACGACCTGATCTCCGGCCCCTCGGGCCTGCTGCTCGCGCTGTGCGCGACGGAACCGGAGCTGCCCGTGCCGCGACCGCTCGC
CGCGCATCTGGCGCTGCTGTGCGACGAGGAGGAGCTGGGGCGGCTGCGCGCGGGCCAGTACCCGGACCACCCGCTGCTGGGCTGGATGCAGGGCCGCGTCAACACC
GGTATGGGGCACGGTGTCGCCGGGCTCGTCACCGCGCTCACCGCGGCACTGCGCCGCACCGGGCCGGACACGGATCTGGCCGGCGACCTCGCCGGGGCGCTCGGCC
GGGCCACCCGCTGGCTGGTCCGCCAGGCGTACGACGACGAGCGGGGCATCCGCACCTGGCCCGGCGCCGGACTCGCCGAGCCGCCCTCCCCCGAGGCCAACCCCCG
CCAGGCGTGGTGTTACGGCACGCCGGGGGTCGCCTGGGCCCTGTGGGACGCCGCCGACGCCCTCGGGGACGGGGCCGCCGCCGACTGGGCCGCGACCGCCTTCACC
ACGCTCGCCGAGCACTACGACGAGACGTTCCACCTCTTCGGCGACGCCCCCGGCGACCGGCTCGGGCTGTGCCACGGGGCCGCGGGCGTGCTGGCGGTCGCCGACG
CCTTCGACCGCCACGCCCGGCTGCCGGCGGCGAGCGCGCTGAAGGACCGGCTCGTCGGCCATCTGACGGCCCGGCTGGCCGTCCGGCCGCTCGCCGAGTGGCCCGC
GGACCTCCTCACCGGCCTGCCGGGGGCGCTGGCCGCGCTGCTGACCGCCGCCCATGGGGCACCACGCGGCTGGCTGCCCTGCCTGGGGCTGCGCTGACGTGGGCGG
CACGGGGCCGACGCCCCGCGCTCCCCGGCCGGCGCTCCGTCTCTTCGCGGCGAGGTCCCGCCACCCGGCGGGGGTGGTCCGGCCACCTGCCGGATGGTTCCACCGG
TGCTGCTGCCTGGAGTGTGGTGACTGGCGAGCACCCCCTGCTCGACGGGCACCCCCTGCTCACCGGGCCGGTGAGGTGCCGCTCCGTGCGGAGAAGGAGGCGGATG
GCGGATGGTGACCGTGCGACTGACGATGCTGTGCGCGACCGCCACACCGGGCGGCCCGGACCGGGTCCTCGAGAACGAGAGCATGGTCAGCGCCGTGCGGGCGGCC
CTGCCGCCGTACGCGCGGGCCCTGCGGGCGCCCTCGGCCCGCTGCGCGCGGATCGCCGGGGCGCTGGCGGTCGAGGCCACGCCGGAGGAGACCCTGCGGGACCTTG
ACTACGGCGCGTGGGGCGGCCGTCCGCTGCGGGACGTCGCGGCCGAGGACCCGCACGGCCTGTCCGCGTGGCTGACGGACCCCGAGGCCGCACCGCACGGCGGCGA
GTCGGTCGCCCGGCTCTGCGACCGTACGGCGGCCTGGCTGACGGCGCTGCCCGGCGGCCCGGGCCTGCTTGCGATCACCGAGGCCGCGGTGATCCGGGCCGCGCTG
GTCCATGCCCTGACCGTGCCCGCCCGCGCGTTCTGGCAGCTGCGGGTGCCGCCGCTGTCGACGGTGACGCTGACGCTGCGCAACGGCCACTGGACGGCCCGGCTCA
CACCGCCCTCGGCACCGCGGCGGCTGCCGCCGACGGCCGGGGAGAGCGCCCCCGTCACCGTACTGACCCTGCCCGCCGGCCGGGATCGGCTCTGCCTCGCCGGACG
GCCGTGACCTGACCCACCGCGCGGCCACCGTG
CCAGGACACCGGGCGGCAGCCCCGTACCGGTACGGCACCAGGCCCGCGAAGACGACCGGGACGCCCCCCCGGCGGCGCGGTCGCGCCCGGCGTGGTACGAATCGGA
CACAGTACTCCCACACAGGCTGGAGCACACGTGATCCGGGTACTGGTGGTCGACGACGAAGCCCTGATCCGCACGGGCTTCCAGCGCATTCTGGACGCGGCCGACG
GCATCGAGGTCGTGGGGGCGGTCTCCGGCGGTCAGGCCCTGCGGGTGGTACGGGAGACGCGTCCGGATGTGGTCCTGCTGGACATCCGCATGCCGGACGTGGACGG
GCTGACCGTGCTGAGCGACATCCGGCGGCTGCCGGAGCCTCCGGTGGTGGCCATGCTGACCACGTTCGACATGGACGAGTACGTGGAGACGGCGCTGCGCTCGGGC
GCCGCCGGCTTCCTGCTGAAGGACACCGATCCCGAGGCGCTGCCGCCGGCGGTGCAGAGCCTGGCCCGCGGTGGCACGGTGCTGTCGCCCAAGGTCACGCGCACGG
TGGTGGACGGCTATCTGAACGCGGGAGCGCAGGAGCGCCCGCCGAAGGCTCTGGAGCGGCTGACCGAGCGTGAGCGCGAGGTGCTCGTGCTGATCGCCGAGGGCCT
GTCGAACACCGACATCGCGGCCCGGATGCACCTGAGCACCGGCACCGTGAAGGACCATGTCAGCGCGATCCTGACCAAGCTGGAGGTCAGCGGCCGGGTCCAGGCC
GCCCTCCTCGCCCAGCGGGCCGGGCTCCTCGCGGAGGGCACGGGATGACCCGCCGGCCGTCCCCGCTGCTCGTGGACGGCGTGGCCGTGGCCATGGCGCTCCTCGA
CGTGTGGGCGCACTGGGACATCGACGAGCCGGTGCGGATGGCCTGCGCGCTGGCCGCCGCGGTCGCCCTGCTGCTGCGCCGGCCGCAACCGCTGCTCACCTTCCTG
CTCACGCTGCCCGCGGTCCTGCTGTCCGACTCGGTGTTCGCCTGCCTGGCCGCGCTGTACACGCTCGCCTCGCTCAGCCGTCCCCGGGCGCTGCTGGTGGTGTGCG
CCCTGGCGTTCGCGGTCAGCGACACGACGTCGCTGCCGTCGCCGGAGCTGGATCTGACGCGTACCCCGACACTGATCGCACTCGGTTACAGCTCCGCCACGGCGGC
GGCGGCCGTCTTCCTGGGACAGCTGGTGCGGACCCGTCGCGATCTGTCGCTGAGGCTGGCGGAGATCTCGGAGGCGCGTGACCATCAGCAGTTGCTGACCGCCCAG
GCCGTCCTCGCCAAGGAGCGCGCGCAGCTGGCACGGGAGATGCACGATGTCGTCTCCCACCAGGTCAGTCTGATCGCCGTACGTGCCGGAGCGCTGCAAGTGGGTG
CGGGCGACCCGGACACCAGACAGGCCGCGGCGACGATCCGGCGGCTGAGCGTGCAGACCCTGGACGAACTGCGCCACATGGTCGGTGTCCTGCGGGCCTCCAGCGG
CCACCCCACGGAGCTCACCCCGCAGCCGGCGCTCAGCGACATCCGGCGGCTGGCCGACAACTGCGGCATCAGGACCGAGTTGCGCATGGCCCTGCCCGAAGGTCTT
CCGCCGACCGTCCAGCGCGCGGTCTACCGCACGGTCCAGGAGGCGCTGACCAACACCCGCAAACACGCTCCGGGGGCGACCGCGCACATCGATCTGAGCCATGCCG
ACGGAGCCGTCCGGGTCCGGGTCACCAACACGGCGCCGACCCAGCCGCCGCTGCCCCTGCCCGGGGCGCACCACGGTCTGCTGGGTCTGCGCCAGCGTGCCGAACT
CCTCGGCGGCACCATGACGTCGGGCCCCACCAGTGACGGCGGCTATGAGCTGTGTGTCGTGCTGCCGGCCCAGGACACGAGGTGAGCGCACGCGTCGG

Heterologous expression strategy and cloning of the expression plasmids

Initially, the two expression plasmids were cloned in a sequential manner. Given that the precursor peptide was not observed when using His$_6$-SptA, neither in the unmodified, fully modified, or in intermediate modification states, the precursor peptide could be sensitive to proteolysis when expressed in *E. coli*. In the absence of the *T. bispora* GluRS/tRNA^{Glu} pair and after fusing the precursor to MBP, we observed a mass corresponding to an N-terminal fragment of Ser-SptA (SMPAHEITELDTLISDLEERITDEDLPTA) after TEV treatment of the isolated MBP fusion. This finding demonstrates that the installation of the (Me)Lan moieties, which are crucial for proteolytic stability of the core peptide, only takes place in the presence of a suitable glutamyl-tRNA^{Glu} substrate. Notably, this truncated peptide was also detected as a side product in the

successful heterologous production experiment (Figure S1), which suggests that the modification of the core peptide competes with the proteolytic degradation in *E. coli*. Such behavior was observed previously also for precursors of class IV lanthipeptides from other *Streptomyces* strains.[7-10] Indeed, use of a fusion of the maltose binding protein (MBP) to the N-terminus of the precursor peptide and the *T. bispora* GluRS/tRNA^{Glu} pair resulted in detection of modified product. Thus, MBP appeared to protect the N-terminus of SptA against aminopeptidases, while the macrocycles installed in the core peptide by the SapT biosynthetic machinery may have provided protection of the C-terminus against degradation by *E. coli* proteases.

PCR reactions were carried out using the primers listed in Table S1. *S. tendae* Tü901 gDNA was used as template for the amplification of *sptA*, *sptB$_a$*, *sptB$_b$*, and *sptC*. Vector backbones were amplified from corresponding plasmid DNA and the *mbp* gene (including a sequence coding for a C-terminal TEV protease cleavage site) was amplified from a plasmid used in a previous study.[8] DNA amplification was performed with Phusion DNA polymerase for the plasmid backbones, *mbp*, *sptA*, *sptB$_b$*, and *sptC*. The 2784 bp long *sptB$_a$* gene however proved recalcitrant to direct amplification in one piece. Hence, it was amplified as two overlapping fragments. The first fragment (1387 bp, using the primers FP-Spt-B$_a$ + RP-Spt-B$_a$_internal) was amplified using Phusion DNA polymerase, whereas amplification of the second fragment (1418 bp, using the primers FP-Spt-B$_a$_internal + RP-Spt-B$_a$) could only be accomplished with KAPA HiFi DNA polymerase.

Table S1. Oligonucleotide primers used in this study. Overhangs for Gibson Assembly are underlined.

name	sequence
**cloning of *his6-SptA* (MCS1) : - (MCS2) pRSF Duet**	
FP-Spt-A	ATG CCC GCA CAC GAA ATC ACC GAA CTC GAC ACC CTG
RP-Spt-A	TCA TCC ACA GAT GAC GAC CGT GGC ACA GAT GAC GAT CG
FP-pRSF-MCS1-inclHis6	ACGGTCGTCATCTGTGGATGA GAT CCG AAT TCG AGC TCG GCG CGC CTG
RP-pRSF-MCS1-inclHis6	GGTGATTTCGTGTGCGGGCAT CTG GCT GTG GTG ATG ATG GTG ATG GCT GCT GCC
**cloning of *SptC* (MCS1) : - (MCS2) pCDF Duet**	
FP-Spt-C	ATG CCG CAC GAC ACG GCC GTA CCG GCC GCC CCG
RP-Spt-C	TCA GCG CAG CCC CAG GCA GGG CAG CCA GCC
FP-pCDF-MCS1-noHis6	CCCTGCCTGGGGCTGCGCTGA GAT CCG AAT TCG AGC TCG GCG CGC CTG
RP-pCDF-MCS1-noHis6	TACGGCCGTGTCGTGCGGCAT GGT ATA TCT CCT TAT TAA AGT TAA ACA AAA TTA TTT CTA CAG GGG AAT TGT TAT
**cloning of *his6-SptA* (MCS1) : *SptB$_b$* (MCS2) pRSF Duet**	
FP-Spt-B$_b$	ATG GTG CGG GTC GAG CGG TTA CTG GCC GAC TGC CTG GAC G
RP-Spt-B$_b$	TCA GTC CGC CTC CTC CCG GCC GTC GGC GGC GAG
FP-his6-A(MCS1)-pRSF-MCS2	GGCCGGGAGGAGGCGGACTGA TCG GTA CCC TCG AGT CTG GTA AAG AAA CCG C
RP-his6-A(MCS1)-pRSF-MCS2	TAACCGCTCGACCCGCACCAT ATG TAT ATC TCC TTC TTA TAC TTA ACT AAT ATA CTA AGA TGG GGA ATT GTT ATC C
**cloning of *SptC* (MCS1) : *SptB$_a$* (MCS2) pCDF Duet**	
FP-Spt-B$_a$	ATG GAA CAC GTA CCG GAA CAA GCG GCC CAG CCA GCC GAC AGT CAG
RP-Spt-B$_a$_internal	CGA GCC TGC GGT AGG AGT CGA GGA ACC AGG CGT CCG
FP-Spt-B$_a$_internal	TCG ACT CCT ACC GCA GGC TCG GCG CCG ACG CC
RP-Spt-B$_a$	TCA TCG GGT GTT GGC CGC CCC GGC GGG CCG CCT G
FP-C(MCS1)-pRSF-MCS2	GGGGCGGCCAACACCCGATGA TCG GTA CCC TCG AGT CTG GTA AAG AAA CCG C
RP-C(MCS1)-pRSF-MCS2	TTGTTCCGGTACGTGTTCCAT ATG TAT ATC TCC TTC TTA TAC TTA ACT AAT ATA CTA AGA TGG
**cloning of *his6-mbp-SptA* (MCS1) : *SptB$_b$* (MCS2) pRSF Duet**	
FP_MBP	ATG AAA ATC GAA GAA GGT AAA CTG GTA ATC TGG
RP_MBP	GGA TTG GAA GTA CAG GTT CTC AGA TCC ACG
FP-his6-A(MCS1)-B$_b$(MCS2)-pRSF	GAGAACCTGTACTTCCAATCC ATG CCC GCA CAC GAA ATC ACC GAA CTC GAC ACC CTG
RP-his6-A(MCS1)-B$_b$(MCS2)-pRSF	TTTACCTTCTTCGATTTTCAT CTG GCT GTG GTG ATG ATG GTG ATG GCT GCT GCC C

Comparison of tRNA^{Glu} sequences

Table S2. Comparison of the tRNA^{Glu} gene sequences of *S. lavenduligriseus* Tü901, *E. coli*, *S. olivaceus* NRRL B-3009, and *T. bispora*. The anticodons are underlined and the bases of the acceptor stem corresponding to the positions shown to be crucial for recognition by the class I dehydratase MibB[11] are shown in red.

***Streptomyces lavenduligriseus* Tü901**	
tRNA^{Glu} 1	GCCCCCGTTGTGTAGCGGCCTAGCACGCTGCCCTCTCAAGGCAGTAGCGCCGGTTCGAATCCGGTCGGGGGTA
tRNA^{Glu} 2	GCCCCCGTTGTGTAGCGGCCTAGCACGCCGCCCTCTCAAGGCGGTAGCGCCGGTTCGAATCCGGTCGGGGGTA
tRNA^{Glu} 3	GCCCCCGTTGTGTAGCGGCCTAGCACGCCGCCCTCTCAAGGCGGTAGCGCCGGTTCGAATCCGGTCGGGGGTA
tRNA^{Glu} 4	GCCCTCATCGTCTAGCGGCCTAGGACGCCGCCCTTTCAAGGCGGTAGCACGGGTTCGAATCCCGTTGGGGGCA
***Streptomyces olivaceus* NRRL B-3009**	
tRNA^{Glu}	GCCCCCGTTGTGTAGCGGCCTAGCACGCCGCCCTCTCAAGGCGGTAGCGCCGGTTCGAATCCGGTCGGGGGTA
Thermobispora bispora	
tRNA^{Glu}	GGTCCCGTCGTCTAGAGGCCTAGGACGCCGCCCTCTCAAGGCGGTAACGGCGGTTCGAATCCGCTCGGGACTA
E. coli	
tRNA^{Glu}	GTCCCCTTCGTCTAGAGGCCCAGGACACCGCCCTTTCACGGCGGTAACAGGGGTTCGAATCCCCTAGGGGACG

HPLC-HRMS conditions

SapT trypsin digestion

Trypsin digest of full-length mSptA (vide infra) was loaded onto an AdvanceBio Peptide Plus (2.7 µm particle size, 150 x 2.1 mm) column. Flow rate was set at 0.6 mL/min with an injection volume of 10 µL. The column was equilibrated with 2% solvent B for 5 min and eluted with a gradient of 2-100% solvent B over 35 min. Mass range for detection and MS/MS was set at m/z 100 – 3200 with ~4 m/z isolation width.

MeLan stereochemical analysis after derivatization with Marfey's reagent (L-FDAA)

Samples were loaded onto a Kinetex Biphenyl (1.7 µm particle size, 100 Å pore size, 50 x 2.1 mm) column. Flow rate was set to 0.5 mL/min with an injection volume of 8 µL. The column was equilibrated with solvent A for 5 min and eluted using a gradient of 0-100% solvent B over 60 min. Targeted m/z = 727.1742 was used for detection of MeLan derivatized with two molecules of L-FDAA.

Analysis and isolation of (Boc)$_2$-MeLan from mSptA hydrolysate

mSptA hydrolysate after derivatization with Boc_2O was loaded onto an AdvanceBio Peptide Plus (2.7 µm particle size, 150 x 2.1 mm) column. Flow rate was set to 0.6 mL/min with an injection volume of 20 µL. The column was equilibrated with 2% solvent B for 5 min and eluted using a gradient of 2-100% solvent B over 35 min. A total of ten runs were performed with a total injection volume of 200 µL. Analysis of the sample was performed using m/z = 423.1796 for detection.

L- and D-Ala stereochemical analysis after reductive desulfurization and derivatization with L-FDAA

Samples were loaded onto an AdvanceBio Peptide Plus (2.7 µm particle size, 150 x 2.1 mm) column. Flow rate was set to 0.6 mL/min with an injection volume of 5 µL. The column was

equilibrated with 2% solvent B for 5 min and eluted using a gradient of 2-100% B over 35 min. Analysis of the sample was performed using m/z = 342.1044 for detection.

GC-MS conditions

GC-MS analysis was performed on an Agilent HP 6890N instrument using a CP-Chirasil-L-Val (Agilent) column (25 m, 0.25 mm x 0.12 µm). Samples previously dissolved in methanol were applied to the column with a total volume of 5 µL in split mode. Temperature was initially kept at 160 ^{o}C for 5 min then increased to 190 ^{o}C over 10 min at a rate of 3 ^{o}C/min and then held at 190 ^{o}C for an additional 10 min. Selective-ion monitoring (SIM) was used to detect mass fragments characteristic for lanthionine (m/z = 365) and methyllanthionine (m/z = 379).[12-14]

Large scale mSptA production and purification

Experimental procedure for heterologous expression and purification of His$_6$-MBP-mSptA

Chemically competent *E. coli* BL21 (DE3) cells were co-transformed with pRSFDuet-1 His$_6$-MBP-SptA_SptB$_b$, pCDFDuet-1 SptC_SptB$_a$, and pTRC33 *T. bispora* GluRS_tRNA^{Glu},[15] and plated onto a LB agar plate containing 17 µg/mL kanamycin, 17 µg/mL spectinomycin, and 8 µg/mL chloramphenicol. The plate was incubated overnight at 37 ^{o}C. A single colony was picked and grown in a 50-mL Terrific Broth (TB) overnight starter culture at 37 ^{o}C containing the appropriate antibiotics and 0.4% glycerol. Next, 1 L of TB containing appropriate antibiotics was inoculated with 10 mL of the starter culture and grown at 37 ^{o}C with shaking at 160 rpm (five baffled flasks; 5 L total). The temperature of the shaker was reduced to 18 ^{o}C when OD_{600} reached 0.8 and 1 mL of 1 M IPTG (isopropyl ß-D-1-thiogalactopyranoside) was added to each flask. The cells were grown for an additional 16 h at 18 ^{o}C after induction with IPTG. The cells were harvested at 5000g for 20 min at 4 ^{o}C. The cell pellet was resuspended in 80 mL of start buffer (20 mM Tris, pH 7.5, 500 mM NaCl). The resuspended cell pellet was lysed using a high-pressure homogenizer (Avestin, Inc.) and centrifuged at 25,000g for 45 min at 4 ^{o}C to isolate the cell lysate. The cell lysate was loaded on a pre-equilibrated 5 mL HisTrap HP column and washed with 30 mL of wash buffer (20 mM Tris, pH 7.5, 500 mM NaCl, 30 mM imidazole). The column was next linked to an AKTA fast protein liquid chromatography (FPLC) system (GE healthcare). The column was washed using a linear gradient of wash buffer (20 mM Tris, pH 7.5, 500 mM NaCl, 30 mM imidazole) at a flow rate of 2.5 mL/min. Elution buffer (20 mM Tris, pH 7.5, 0.5 M NaCl, 500 mM imidazole) was set at 6% for 5 column volumes (CV), increased to 10% for 3 CV, and increased up to 25% for 0.5 CV. Protein elution was monitored at 280 nm and all elution fractions were analyzed by SDS-PAGE. Fractions that contained His$_6$-MBP-SapT were combined and concentrated using an Amicon Ultra-15 filter (3 kDa MWCO, Millipore) via centrifugation. Buffer was exchanged to storage buffer (20 mM Tris, 500 mM KCl, pH 7.5) by repeated centrifugation. Final yield for His$_6$-MBP-mSptA was 8 mg/L.

His$_6$-MBP removal and purification of MBP-mSptA

His$_6$-MBP-SptA contains a TEV recognition site between the MBP and SptA sequences. Thus, His$_6$-TEV protease (1.6 mg) was incubated with 16 mg of His$_6$-MBP-mSptA in 5 mL of storage buffer in a 15 mL conical tube, followed by the addition of 0.5 mL of HisPur^{TM} Ni-NTA resin. The sample was left to rock overnight at 37 ^{o}C. After overnight incubation, the sample was transferred to an Econo-Pac chromatography column. The flow through was collected and the column was

washed with an additional 20 mL of acetonitrile. The fractions were combined and lyophilized. After lyophilization, the sample was resuspended in 10 mL of 5:1 H_2O:MeCN and sonicated. After sonication, mSptA was purified using preparative reversed-phase high-performance liquid chromatography (HPLC) using a Macherey Nagel C18 HTec column (10 µm particle size, 100 Å pore size, 250 x 21 mm). HPLC buffers consisted of 0.1% trifluoroacetic acid (TFA) in H_2O (solvent C) and 0.1% TFA in MeCN (solvent D). A linear gradient starting from 98% buffer C to 100% buffer D over 24 min was used to purify mSptA. mSptA eluted at 30:70 solvent C: solvent D with a final yield of 1 mg.

N-ethylmaleimide (NEM) assay

An NEM assay was carried out to determine if mSptA was fully cyclized. mSptA (100 µM) was incubated with 10 mM TCEP, 10 mM NEM in 50 mM Tris buffer containing 500 mM NaCl at pH 7.5. Total reaction volume consisted of 100 µL in a 0.5 mL Eppendorf tube. A negative control was performed in which TCEP and NEM were not added. After 1 h of incubation at room temperature the samples were analyzed by MALDI-TOF MS. mSptA is likely fully cyclized given that NEM adducts were not detected (Figure S1).

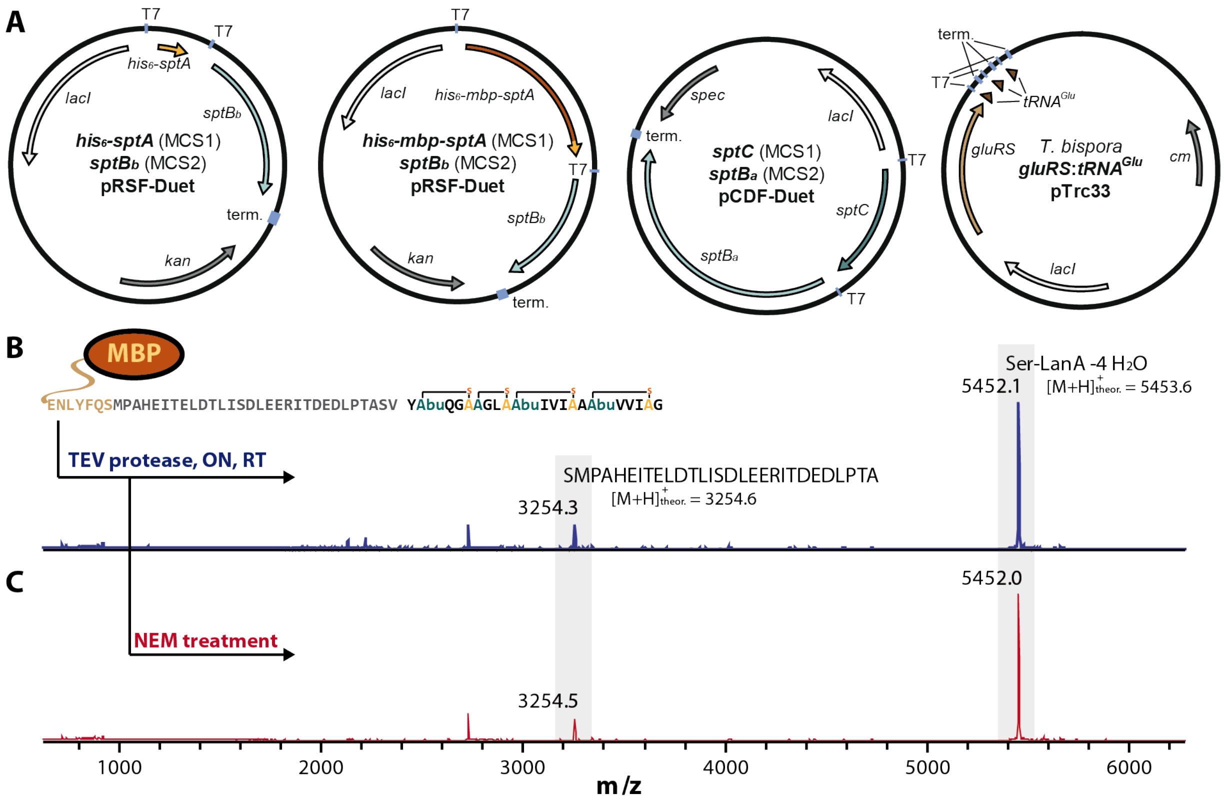

Figure S1. MALDI-TOF MS analysis of mSptA after removal of His$_6$-MBP tag. A) Plasmid maps of expression vectors. B) Four-fold dehydrated product was detected along with a truncated peptide with the sequence shown. C) NEM alkylation assay provides strong support that mSptA is cyclized as no NEM adducts are observed.

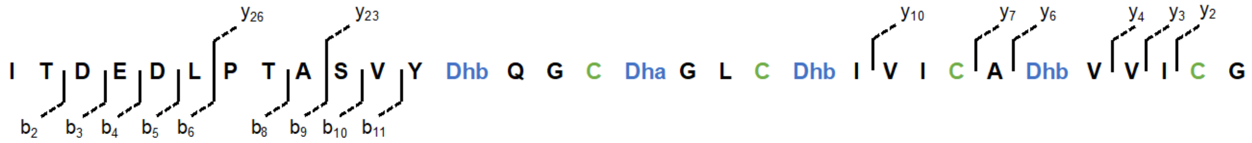

Figure S2. Observed b- and y-ions from tandem MS analysis of the C-terminal fragment of trypsin-digested mSptA. Observed ions are annotated in **Figure S3** and compared to calculated m/z ratios in **Table S3.** The observed fragment ions clearly localize the dehydrated residues. Ions arising from fragmentation within rings are minor and have been observed previously.[16]

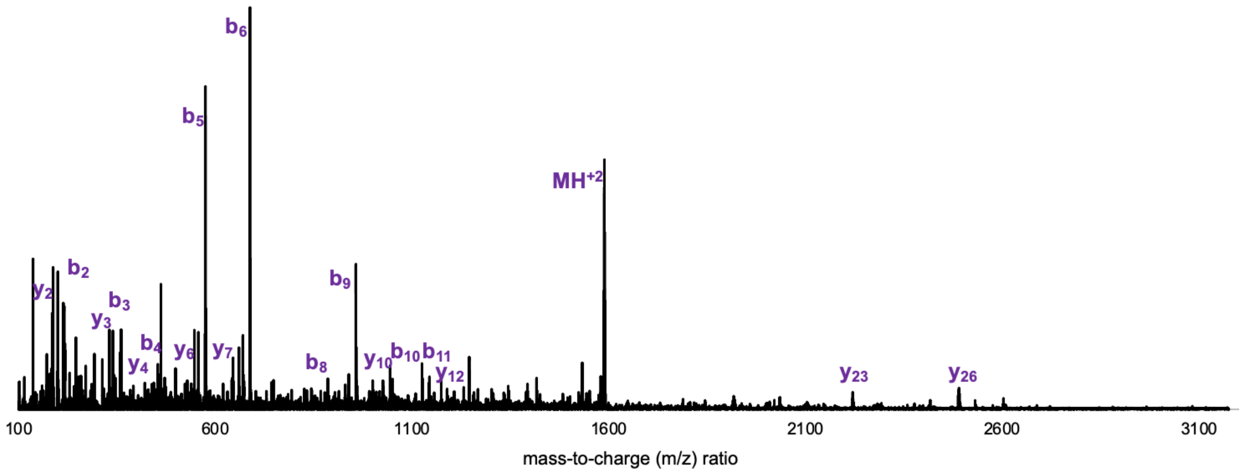

Figure S3. LC-MS/MS analysis of mSptA C-terminal region after trypsin digestion. Observed b- and y-ions are labeled (see Table S3).

mSptA trypsin digestion and tandem MS analysis

mSptA was digested with sequencing grade trypsin (50:1, peptide: protease) in 50 mM Tris, pH 7.5 buffer at 37 °C for 3 h with a total reaction volume of 50 µL. After 3 h, the sample was desalted using ZipTip C18 pipet tips and subjected to LC-MS analysis.

Table S3. Calculated and observed m/z for the C-terminal region of mSptA after trypsin digestion.

Ion	Calculated m/z	Observed m/z	Error (ppm)
y_2	179.0485	179.0473	6.702
b_2	215.1390	215.1390	0.000
y_3	292.1326	292.1327	0.342
b_3	330.166	330.1647	3.937
y_4	391.2010	391.1997	3.323
b_4	459.2086	459.2090	0.871
y_6	573.3065	573.3050	2.616
b_5	574.2355	574.2359	0.697
y_7	644.3436	644.344	0.621
b_6	687.3196	687.3185	1.600

**b_8**	885.4200	885.4183	1.920
**b_9**	956.4571	956.4557	1.464
**y_{10}**	959.5053	959.5030	2.397
**b_{10}**	1043.4891	1043.4838	5.079
**b_{11}**	1142.5576	1142.5602	2.276
**y_{12}**	1155.6264	1155.6221	3.721
**MH^{+2}**	1587.2549	1587.2532	1.071
**y_{23}**	2218.0527	2218.0586	2.660
**y_{26}**	2487.1903	2487.1750	6.152

Hydrolysis and derivatization of mSptA for GC-MS analysis

mSptA (1 mg) was dissolved in 3 mL of 6 M DCl in D_2O in a sealed pressure tube and heated to 110 ^oC for 24 h. The mixture was transferred to a 15 mL round-bottom flask (RBF) and the solvent was removed under reduced pressure. In a separate 15 mL RBF, 1.5 mL of acetyl chloride was added to 5 mL of methanol on an ice-water bath. Next, 3 mL of the resulting solution was transferred to the RBF containing the hydrolyzed peptide. The solution was heated to 110 ^oC for 1 h under reflux. The sample was then cooled to room temperature and solvent was removed under reduced pressure. Next, 1 mL of pentafluoropropionic anhydride in 3 mL of dichloromethane was added to the reaction flask and the mixture was heated to 90 ^oC for 30 min under reflux. The solution was cooled to room temperature and solvent was removed under a gentle stream of nitrogen. The final oil was dissolved in 200 µL of methanol and stored at -20 ^oC prior to GC-MS analysis (vide supra).[13]

Genome Mining for SapT and SapB Homologs

For the genome mining for homologs of the SapT and SapB BGCs, the corresponding precursor peptide sequences were used as input for the *Basic Local Alignment Search Tool* (BLAST) using the blastp suite.[17] Two rounds of a position-specific iterated BLAST (PSI-BLAST)[18] with default settings were performed for each input. The thus-obtained hits were curated manually to remove false-positive hits. The obtained precursor homologs are listed alongside their accession numbers and host strains in Table S8. Only one entry was included in the table per identical precursor peptide sequence listing a representative organism. The corresponding accession numbers are however the same for all identical peptide sequences found in the GenBank databases.

Sequence Similarity Network Analysis

To allow a better comparison of the putative lanthipeptides derived from the precursor peptides identified through genome mining, a sequence similarity network (SSN) was generated using the core peptide sequences as input for the *Enzyme Function Initiative - Enzyme Similarity Tool* (ESI-EST)[19] with a set alignment score threshold of 5. The separation of the precursors into the corresponding leader and core peptide regions was accomplished through manual curation of the precursor peptide sequences on the basis of the reported sequences of SapT and SapB. The manual separation was targeted at providing core regions with high similarity to the sequences of these lanthipeptides.

Conserved Motif Prediction

The *Multiple Expectation Maximizations for Motif Elicitation* (MEME) algorithm[20] and the corresponding MEME webtool were employed to identify conserved motifs representing the core and leader regions of the precursor peptides found through genome mining. Separate motif predictions were carried out for the SapT and SapB precursors using only their leader or core peptide sequences as inputs. The obtained motif logos were based on 42 out of the 42 SapT leader sequences, 30 out of the 42 SapT core sequences, 232 out of the 241 SapB leader sequences, and 231 out of the 241 SapB core sequences, respectively.

Small molecule synthesis

Synthesis of N-Boc-L-allo-thiothreonine (1A)

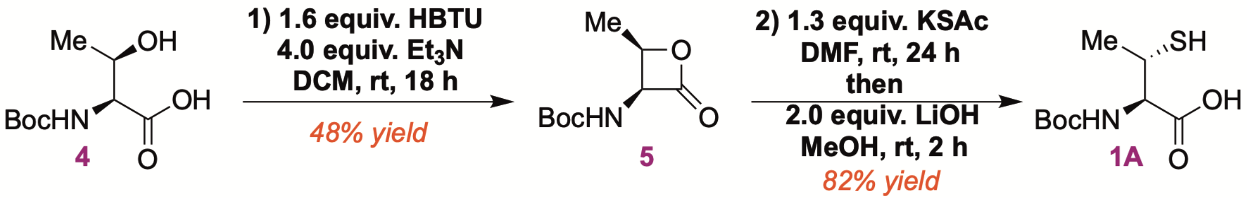

Figure S4. Synthesis of *N*-Boc-L-*allo*-thiothreonine (**1A**).

N-Boc-L-Threoninyl-β-lactone (5)

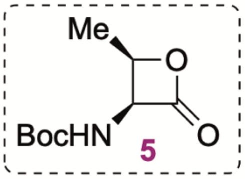

N-Boc-L-Thr-OH (**4**) (1.00 g, 4.56 mmol) and HBTU (2.8 g, 7.3 mmol, 1.6 equiv.) were added to a 100 mL RBF equipped with a magnetic stir bar. Next, 50 mL of anhydrous DCM was added, followed by the addition of triethylamine (2.5 mL, 18 mmol). The reaction mixture was left to stir overnight at room temperature for 16 h. The mixture was then concentrated under reduced pressure. The crude mixture was purified by flash chromatography (silica gel, hexane to 3:1 hexanes:EtOAc) to yield *N*-Boc-L-threoninyl-β-lactone (**5**) as a white solid (442 mg, 48% yield).

**R_f**	0.7 (SiO_2, hexanes:EtOAc = 1:1, $KMnO_4$)
**^{1}H NMR**	(500 MHz, DMSO-d_6) δ 7.95 (d, *J* = 9.4 Hz, 1H), 5.36 (dd, *J* = 9.5, 6.1 Hz, 1 H), 4.82 (app p, *J* = 6.3 Hz, 1 H), 1.40 (s, 9H), 1.34 (d, *J* = 6.3 Hz, 3H).
**^{13}C NMR**	(126 MHz, DMSO-d_6) δ 170.2, 154.8, 79.2, 74.7, 59.6, 28.0, 14.5.
**hrMS**	(ESI-TOF, positive mode). $[M+Na]^+$ calc.: 224.0899; found: 224.0899.

N-Boc-L-allo-thiothreonine (1A)

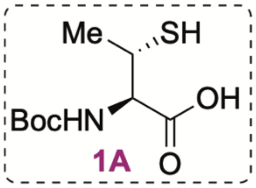

N-Boc-L-threoninyl-β-lactone (**5**) (400 mg, 2.0 mmol) and potassium thioacetate (300 mg, 2.6 mmol) were added to a 100 mL RBF equipped with a magnetic stir bar. Next, 30 mL of anhydrous DMF was added and the reaction mixture was left to stir for 24 h at room temperature. The reaction mixture was then diluted with 30 mL of EtOAc and transferred to a 250 mL-

separatory funnel. Next, 30 mL of 1 M HCl was added and the layers were separated. The aqueous layer was extracted again with an additional 30 mL of EtOAc. The aqueous layer was discarded and the organic layers were combined and washed twice with aqueous LiCl (5% solution, 30 mL). The organic layer was dried over magnesium sulfate, filtered, and concentrated under reduced pressure to yield *N*-Boc-*S*-acetyl-L-*allo*-thiothreonine as a yellow oil. Next, *N*-Boc-*S*-acetyl-L-*allo*-thiothreonine (450 mg, 1.6 mmol) was taken up in 32 mL of MeOH in a 100 mL RBF. To this solution was added 3.8 mL of 1 M LiOH. The reaction mixture was left to stir at room temperature for 2 h. Next, the pH of the reaction mixture was adjusted to 1 with a 1 M HCl solution. The mixture was transferred to a 250 mL-separatory funnel and the aqueous layer was extracted with 60 mL of EtOAc (repeated 2X). The organic layers were combined, dried over magnesium sulfate, filtered, and concentrated under reduced pressure. *N*-Boc-L-*allo*-thiothreonine (**1A**) was purified by flash chromatography (silica gel, 1:1 hexanes:EtOAc with 2% AcOH) to afford a yellow oil (350 mg, 82% yield).

**R_f**	0.4 (SiO_2, hexanes:EtOAc:AcOH = 3:1:0.03, $KMnO_4$)
**^{1}H NMR**	(500 MHz, DMSO-*d_6*) δ 12.57 (s, 1H), 7.12 (d, *J* = 9.0 Hz, 1H), 4.07 (d, *J* = 9.0, 6.0 Hz, 1H), 3.26 (m, 1H), 2.61 (d, *J* = 7.8 Hz, 1H), 1.39 (s, 9H), 1.24 (d, *J* = 7.0 Hz, 3H).
**^{13}C NMR**	(126 MHz, CD_3CN) δ 172.2, 156.7, 80.3, 60.7, 37.1, 28.5, 21.4.
**hrMS**	(ESI-TOF, negative mode). $[M-H]^-$ calc.: 234.0800; found: 234.0795.

Synthesis of N-Boc-D-allo-thiothreonine (2A)

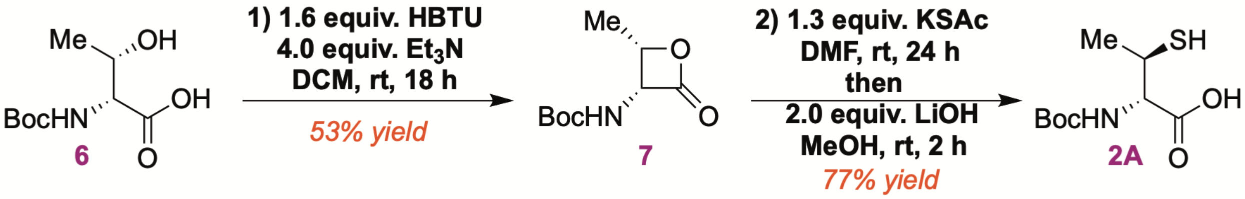

Figure S5. Synthesis of *N*-Boc-D-*allo*-thiothreonine (**2A**).

Identical synthetic procedures with reactions performed on similar scales were used for the synthesis of **7** and **2A** as highlighted in the previous section for **5** and **1A**. Characterization data is provided below for the products.

N-Boc-D-Threoninyl-β-lactone (7)

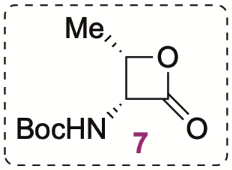

**R_f**	0.7 (SiO_2, hexanes:EtOAc = 1:1, $KMnO_4$)
**^{1}H NMR**	(500 MHz, DMSO-*d_6*) δ 7.95 (d, *J* = 9.4 Hz, 1H), 5.36 (dd, *J* = 9.5, 6.1 Hz, 1H), 4.82 (app p, *J* = 6.3Hz, 1H), 1.40 (s, 9H), 1.34 (d, *J* = 6.3 Hz, 3H).
**^{13}C NMR**	(126 MHz, DMSO-*d_6*) δ 170.2, 154.8, 79.2, 74.7, 59.6, 28.0, 14.5.
**hrMS**	(ESI-TOF, positive mode). $[M+Na]^+$ calc.: 224.0899; found: 224.0898.

N-Boc-D-allo-Thiothreonine (2A)

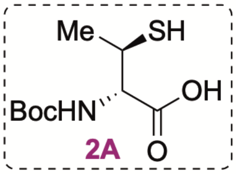

R$_f$ 0.4 (SiO_2, hexanes:EtOAc:AcOH = 3:1:0.03, $KMnO_4$)

^{1}H NMR (500 MHz, DMSO-d_6) δ 12.73 (s, 1H), 7.12 (d, J = 9.0 Hz, 1H), 4.07 (dd, J = 9.0, 6.0 Hz, 1H), 3.25 (m, 1H), 2.61 (bs, 1H), 1.39 (s, 9H), 1.24 (d, J = 7.0 Hz, 3H).

^{13}C NMR (126 MHz, CD_3CN) δ 172.3, 156.7, 80.3, 60.8, 37.1, 28.5, 21.4.

hrMS (ESI-TOF, negative mode). $[M-H]^-$ calc.: 234.0800; found: 234.0793.

Synthesis of N-Boc-Dha-OMe (3A)[21]

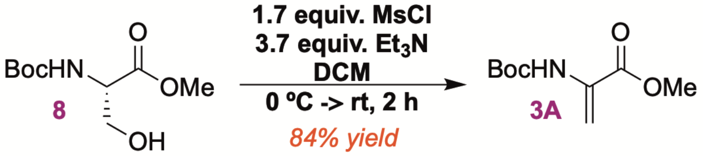

Figure S6. Synthesis of *N*-Boc-Dha-OMe (**3A**).

Boc-Dha-OMe (3A)

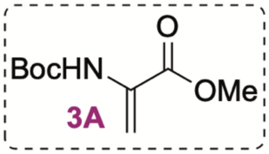

N-Boc-L-serine methyl ester (**8**) (1.00 g, 4.56 mmol) was added to a 100 mL RBF equipped with a magnetic stir bar. Next, 20 mL of anhydrous DCM was added to the RBF and the flask was cooled to 0 °C in an ice-water bath. Methanesulfonyl chloride (600 µL, 7.75 mmol, 1.7 equiv.) was added dropwise to the stirred solution. Next, triethylamine (2.30 mL, 17.0 mmol, 3.7 equiv.) was added to the reaction flask. The reaction mixture was stirred for 1 h at 0 °C. Then, the ice-water bath was removed and the mixture was stirred for an additional 2 h at room temperature. The reaction mixture was transferred to a 125 mL-separatory funnel. Potassium bisulfate (1% aqueous solution, 20 mL) was added and the layers were separated. The organic layer was dried over magnesium sulfate, filtered, and concentrated under reduced pressure. The crude mixture was subjected to flash chromatography (silica gel, 4:1 hexane:EtOAc) to yield *N*-Boc-Dha methyl ester (**3A**) as a colorless oil (770 mg, 84% yield).

Rf 0.8 (SiO_2, hexanes:EtOAc = 4:1, $KMnO_4$)

^{1}H NMR (500 MHz, $CDCl_3$) δ 7.00 (s, 1H), 6.15 (s, 1H), 5.72 (s, 1H), 3.82 (s, 3H), 1.47 (s, 9H).

^{13}C NMR (126 MHz, $CDCl_3$) δ 164.6, 152.7, 131.4, 105.3, 80.8, 53.0, 28.4.

hrMS (ESI-TOF, positive mode). $[M+Na]^+$ calc.: 224.0899; found: 224.0890.

Synthesis of L-allo-D/L-MeLan (1B) and D-allo-D/L-MeLan (2B)

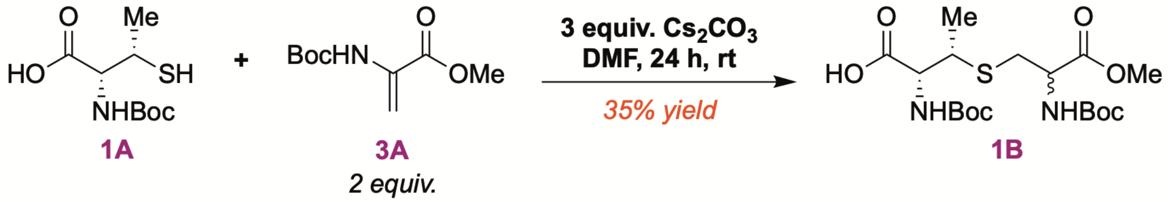

Figure S7. Synthesis of L-*allo*-D/L-MeLan (**1B**).

L-allo-D/L-MeLan (1B)

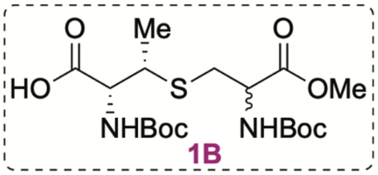

N-Boc-L-*allo*-thiothreonine (**1A**) (60 mg, 0.26 mmol) was placed in a 15 mL RBF equipped with a magnetic stir bar. Next, 251 mg (0.778 mmol, 3 equiv.) cesium carbonate and 1.4 mL DMF was added, followed by the addition of 76 mg (0.38 mmol, 2 equiv.) of *N*-Boc-Dha-OMe (**3A**). The reaction mixture was left to stir at room temperature for 24 h. The reaction mixture was diluted with 15 mL of 1 M HCl and transferred to a 125-mL separatory funnel and extracted with 20 mL of EtOAc (repeated 2X). The aqueous layer was discarded and the organic layers were combined and washed with aqueous LiCl (5% solution, 20 mL) (repeated 3X). The organic layer was dried over magnesium sulfate, concentrated under reduced pressure, and L-*allo*-D/L-MeLan (**1B**) was purified on a Teledyne Isco CombiFlash RF Plus system (RediSep Rf Gold 12g HP Silica column, gradient: initial equilibration with 9:1:0.2 hexanes:EtOAc:AcOH for 3 CV followed by gradient elution from 9:1:0.2 hexanes:EtOAc:AcOH to 3.5:6.5:0.2 hexanes:EtOAc:AcOH over 20 CV). Fractions containing L-*allo*-D/L-MeLan (**1B**) were combined, concentrated under reduced pressure, and lyophilized to yield a white powder (40 mg, 35% yield).

**Rf**	0.3 (SiO_2, hexanes:EtOAc:AcOH = 3:1:0.03, I_2)
**^{1}H NMR**	(500 MHz, CD_3CN) δ 5.94 (br, 1H), 5.85 (br, 1H), 5.77 (br, 1 H), 5.71 (br, 1H), 4.36 (m, 2H), 4.30 (m, 2H), 3.69 (s, 6H), 3.32 (m, 1H), 3.28 (m, 1H), 3.06 (m, 1H), 2.97 (m, 2H), 2.85 (m, 1H), 1.42 (s, 18H), 1.41 (s, 18H), 1.16 (m, 6H).
**^{13}C NMR**	(126 MHz, CD_3CN) δ 172.6, 172.4, 156.9, 156.5, 156.4, 80.3, 80.2, 57.6, 57.3, 54.3, 54.2, 53.0, 52.9, 43.3, 43.0, 33.9, 33.6, 28.6, 28.5, 17.2, 16.8.
**hrMS**	(ESI-TOF, positive mode). $[M+Na]^+$ calc.: 459.1777; found: 459.1773.

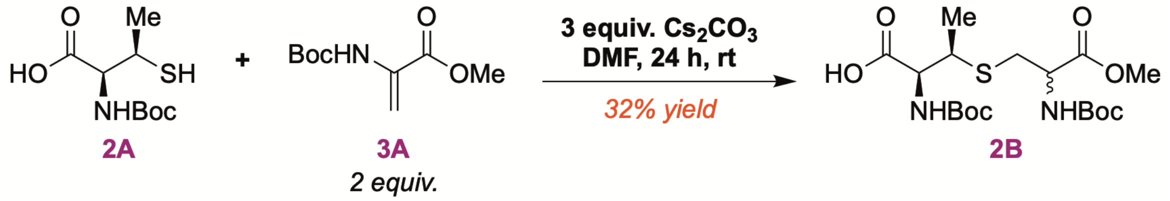

Figure S8. Synthesis of D-*allo*-D/L-MeLan (**2B**).

D-*allo*-D/L-MeLan (**2B**) was synthesized according to an identical procedure described above for **1B**. Characterization data is provided below for **2B**.

D-allo-D/L-MeLan (2B)

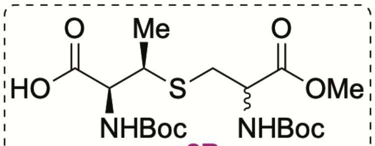

2B

**Rf**	0.3 (SiO_2, hexanes:EtOAc:AcOH = 3:1:0.03, I_2)
**^{1}H NMR**	(500 MHz, CD_3CN) δ 5.95 (br, 1H), 5.85 (br, 1H), 5.73 (br, 2H), 4.36 (m, 2H), 4.28 (m, 2H), 3.69 (s, 6H), 3.33 (m, 1H), 3.29 (m, 1H), 3.05 (m, 1H), 2.96 (m, 2H), 2.86 (m, 1H), 1.43 (s, 18H), 1.41 (s, 18H), 1.14 (m, 6H).
**^{13}C NMR**	(126 MHz, CD_3CN) δ 172.5, 172.3 (2), 172.2, 156.9, 156.4, 156.3, 80.2 (2), 57.5, 57.2, 54.3, 54.2, 53.0, 52.9, 43.1, 42.8, 33.9, 33.6, 28.5 (2), 17.2, 16.8.
**hrMS**	(ESI-TOF, positive mode). $[M+Na]^+$ calc.: 459.1777; found: 459.1760.

Derivatization procedures for GC-MS

Derivatization of D-allo-D/L-MeLan and L-allo-D/L-MeLan for GC-MS

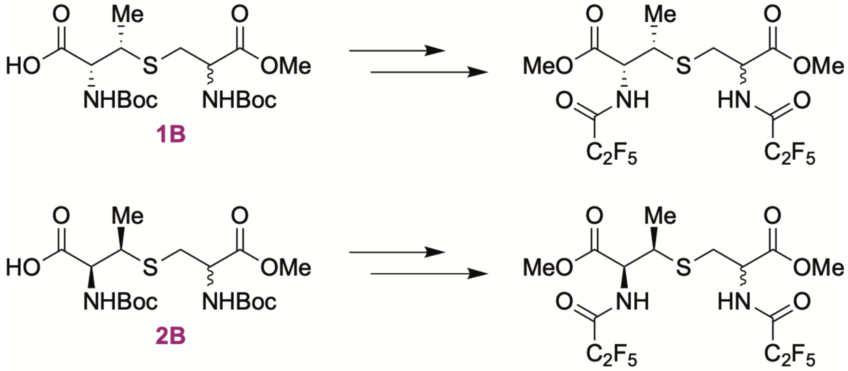

Figure S9. Derivatization of compounds **1B** and **2B** for GC-MS analysis.

D-*allo*-D/L-MeLan (**1B**) or L-*allo*-D/L-MeLan (**2B**) (1.0 mg, 2.29 µmol) was added to a 15 mL RBF containing a magnetic stir bar. Next, 1 mL of 1:1 TFA:DCM was added drop-wise to the RBF. The reaction mixture was left to stir for 1.5 h at room temperature. The reaction mixture was then concentrated under reduced pressure. Next, 5 mL of anhydrous MeOH was added to a separate 15 mL RBF and cooled to 0 °C in an ice-water bath followed by the dropwise addition of 2 mL of AcCl. The ice-water bath was removed after the addition was complete and 3 mL was transferred to the 15 mL RBF containing Boc-deprotected **1B** or **2B**. A reflux condenser was attached and the reaction mixture was refluxed for 1 h at 110 °C. The solution was cooled to room temperature and concentrated under reduced pressure. Next, 3 mL of anhydrous DCM was added to the flask followed by the drop-wise addition of 1 mL of pentafluoropropionic anhydride. A reflux condenser was again attached and the mixture was refluxed at 90 °C for 30 min. The solution was then cooled to room temperature and the solvent was removed using a gentle

stream of nitrogen. The final oil was resuspended in 1 mL of MeOH, diluted 100:1 with MeOH, and stored at -20 °C prior to GC-MS analysis (vide supra).

Derivatization procedures for LC-MS

Derivatization of D-allo-D/L-MeLan and L-allo-D/L-MeLan with Marfey's reagent for LC-MS

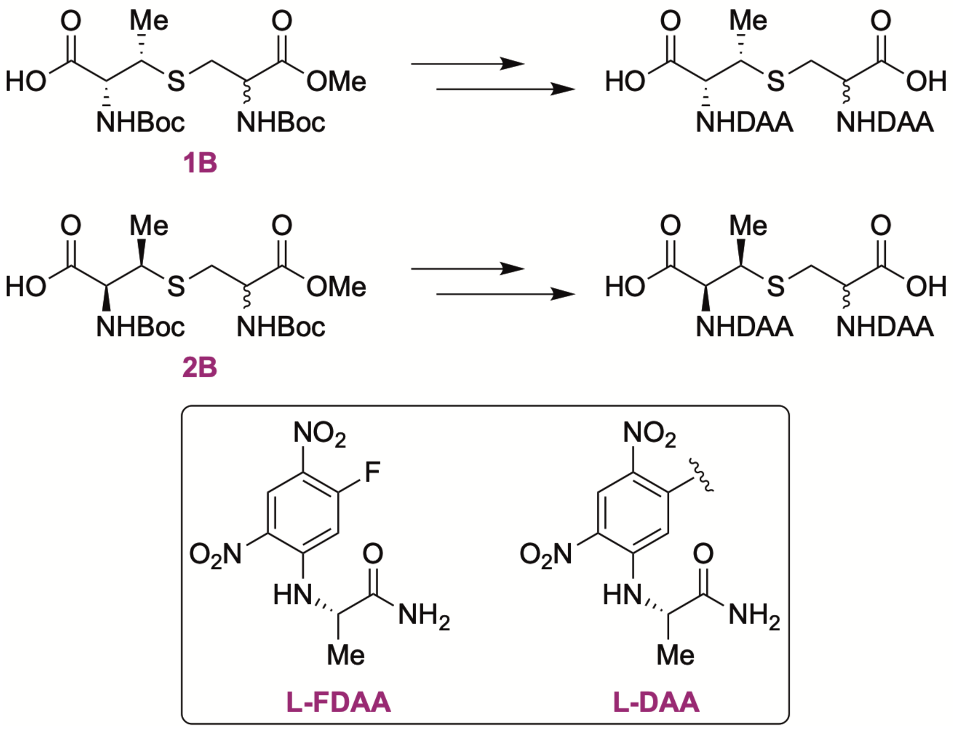

Figure S10. Derivatization of compounds **1B** and **2B** for LC-MS by treatment with Marfey's reagent (L-FDAA). Structure of Marfey's reagent and abbreviations are listed at the bottom of the figure.

D-*allo*-D/L-MeLan (**1B**) or L-*allo*-D/L-MeLan (**2B**) (100 µg, 0.229 µmol) was added to a 5 mL RBF containing a magnetic stir bar. Next, 100 µL of 1:1 TFA:DCM was added drop-wise to the RBF. The reaction mixture was left to stir for 1.5 h at room temperature. The reaction mixture was then concentrated under reduced pressure. Next, 250 µL of 6 M DCl/D_2O was added and the resulting solution was transferred to a sealed-pressure tube and heated to 110 °C for 18 h. After 18 h, the solution was cooled to room temperature and concentrated under reduced pressure. Next, 495 µL of 0.5 M $NaHCO_3$ was added and the mixture was transferred to a 2-dram vial equipped with a magnetic stir bar. Next, 180 µL of L-FDAA solution (1 mg/mL solution in MeCN) was added and the mixture was stirred on a heating block at 63 °C for 3 h. Then, 0.5 mL of 50 mM ammonium formate was added. The mixture was diluted 50:1 with H_2O and stored at -20 °C prior to analysis.

Hydrolysis and derivatization of mSptA for LC-MS with a chiral stationary phase

mSptA (200 µg, 0.03 µmol) was hydrolyzed in 3 mL of 6 M DCl/D_2O in a sealed pressure tube for 24 h at 110 °C. The sample was concentrated under reduced pressure and 220 µL of 0.5

M $NaHCO_3$ was added to the hydrolysate. The solution was transferred to a 2-dram vial equipped with a magnetic stir bar. Next, 80 µL of L-FDAA (1 mg/mL solution in MeCN) was added and the mixture was stirred on a heating block at 63 °C for 3 h. Then, 100 µL of 50 mM ammonium formate was added. The mixture was diluted 10:1 with H_2O and stored at -20 °C prior to analysis.

mSptA reductive desulfurization procedure for LC-MS (adopted protocol)[22]

mSptA (500 µg, 0.09 µmol) was hydrolyzed in 2 mL of DCl/D_2O in a pressure sealed tube for 18 h at 110 °C. Next, solvent was removed under reduced pressure and the resulting oil was resuspended in 100 µL of 1 M aqueous $NaHCO_3$. Next, 25 µL of Boc_2O (1.0 M solution in MeOH) was added and the mixture was heated in a 2-dram vial on a stirring block at 40 °C for 3 h with vigorous stirring. The solvent was removed under reduced pressure and the sample was resuspended in 200 µL of H_2O. The sample was subjected to LC-MS fractionation and fractions containing $(Boc)_2$-MeLan were isolated as described in the LC-MS conditions section. Next, the sample was lyophilized and resuspended in 50 µL of H_2O and transferred to a 2-dram vial equipped with a magnetic stir bar. Next, 20 µL of Raney-Nickel (50% slurry in H_2O) was added under nitrogen. A balloon filled with hydrogen was attached and the gas was bubbled through the solution for 10 min. The hydrogen balloon was removed and the mixture was heated to 50 °C for 2 h on a heating block with vigorous stirring. After 2 h, the solution was transferred to a 1.5 mL Eppendorf tube and centrifuged at 12,000 xg for 2 min. The solution was isolated and lyophilized. Afterwards, the sample was taken up in 2 mL of 6 M DCl/D_2O and heated for 30 min at 50 °C. The sample was then lyophilized. Next, the sample was dissolved in 110 µL of 0.5 M aqueous $NaHCO_3$ and transferred to a 2-dram vial equipped with a magnetic stir bar. Next, 40 µL of L-FDAA (1 mg/mL solution in MeCN) was added and the vial was heated at 63 °C for 3 h on a heating block. After 3 h, 50 µL of 50 mM ammonium formate was added. The mixture was stored at -20 °C prior to analysis.

Derivatization of L- and D-Ala with Marfey's reagent for LC-MS

L- or D-Ala (98 µg, 1.1 µmol) was dissolved in 500 µL of 1 M $NaHCO_3$ and transferred to a 2-dram vial equipped with a magnetic stir bar. Next, 200 µL of L-FDAA (0.6 mg/mL solution in MeCN) was added and the mixture was stirred on a heating block at 63 °C for 3 h. After 3 h, 500 µL of 50 mM ammonium formate was added. The mixture was diluted 50:1 with H_2O and stored at -20 °C prior to analysis.

Bioinformatics Analysis

Sequence alignment of biosynthetic enzymes was conducted by first performing a BLAST analysis[23] with $SptB_b$ as query followed by RODEO analysis[24] to assure the presence of genes encoding a precursor peptide, glutamylation domain of LanB, and LanC. Sequences were aligned with either NisB[25] and MibB[11] for elimination domains or NisC[26-27] and MibC[11] for cyclases.

	215	285	332
NisC	LAHGLA	RDAWCY	MICHG
MibC	LAHGAA	RTAWCY	TVCHG
SptC	MGHGVA	RQAWCY	GLCHG
WP_026207933.1	LAHGLP	RQAWCY	GFCHG
WP_063751904.1	MAHGAA	RQAWCY	GLCHG
MBA8925492.1	MAHGAA	RQAWCY	GLCHG
WP_179771689.1	LAHGVA	RQAWCY	SLCHG
WP_073487578.1	LAHGAA	RQAWCY	AVCHG
WP_189052780.1	LGHGVP	RQAWCY	AICHG
WP_078874016.1	MGHGAA	RQGWCY	GVCHG
WP_159785521.1	MGHGAA	RQGWCY	GVCHG
WP_016468356.1	MGHGAA	RQAWCY	GLCHG
EPD95943.1	MGHGAA	RQAWCY	GLCHG
WP_114022783.1	MGHGVA	RQAWCY	GLCHG
WP_114017317.1	MGHGVA	RQAWCY	GLCHG
WP_020930404.1	TGHGVA	RQAWCY	GLCHG
WP_066979278.1	MGHGVA	RQAWCY	GLCHG
WP_019546723.1	MAHGAA	RQAWCY	GLCHG
WP_146478879.1	TGHGVA	RQAWCY	ALCHG
WP_040916945.1	TGHGVA	RQAWCY	GICHG
WP_148835524.1	MGHGVA	RQAWCY	ALCHG
WP_093548551.1	MGHGVA	RQAWCY	ALCHG
WP_124717588.1	MGHGVA	RQAWCY	ALCHG
WP_078897616.1	MGHGVA	RQAWCY	ACCHG
WP_150250717.1	MGHGVA	RQAWCY	ACCHG
WP_180333154.1	LGHGVA	RQAWCY	GLCHG
WP_079135962.1	TGHGAA	RQAWCY	ALCHG
MBX7552014.1	MGHGAA	RQAWCY	ALCHG
WP_225100127.1	MGHGTA	RQAWCY	ALCHG
WP_158928461.1	MGHGVA	RQAWCY	ALCHG
WP_053656160.1	MGHGTA	RQAWCY	ALCHG
WP_078915291.1	MGHGAA	RQAWCY	GLCHG
WP_079139994.1	MGHGAA	RQAWCY	GLCHG
WP_023589888.1	MGHGAA	RQAWCY	GLCHG
WP_189537587.1	MGHGVA	RQAWCY	GLCHG
WP_126886902.1	MGHGVA	RHAWCY	GLCHG
WP_156695816.1	MGHGVA	RQAWCY	GLCHG
WP_084904933.1	MGHGAA	RQAWCY	GLCHG
WP_031080176.1	MGHGVA	RQAWCY	GLCHG
WP_037854955.1	MGHGVA	RQAWCY	GLCHG
WP_064536951.1	MGHGVA	RQAWCY	GLCHG
WP_037890515.1	MGHGVA	RQAWCY	GLCHG
WP_018569784.1	MGHGVA	RQAWCY	GLCHG
WP_094375420.1	MGHGVA	RQAWCY	GLCHG
WP_159674756.1	MGHGVA	RQAWCY	GLCHG
WP_189798847.1	MGHGVA	RQAWCY	GLCHG
GHF94085.1	MGHGVA	RQAWCY	GLCHG
WP_125497334.1	MGHGVA	RQAWCY	GLCHG
WP_053212511.1	MGHGVA	RQAWCY	GLCHG
WP_196942624.1	MGHGVA	RQAWCY	GLCHG
WP_030991584.1	MGHGVA	RQAWCY	GLCHG
WP_031097958.1	MGHGVA	RQAWCY	GLCHG
WP_189991832.1	MGHGVA	RQAWCY	GLCHG
WP_030602285.1	MGHGVA	RQAWCY	GLCHG
WP_189753200.1	MGHGVA	RQAWCY	GLCHG
WP_030747656.1	MGHGVA	RQAWCY	GLCHG
WP_189302515.1	MGHGVA	RQAWCY	GLCHG

Figure S11. Partial sequence alignment of SptC homologs identified by RODEO[24] with NisC and MibC. Critical active site residues such as the His active site acid that protonates the enolate (yellow), conserved Arg (blue), and zinc-binding residues (gray) are fully conserved in SptC homologs as well as canonical LanC enzymes.

	796	834
NisB	NLGGNLFFLRYTDP-KPHIRLRIKCSDLFLAY	DQEVERYGGFDTLE
MibB	G-ADRWFFIRYSDTAGQHLRVRFRGEREKLWA	DPEYERYGGDALAE
$SptB_b$	GQARDFFFMHK----PPGLRVRFLAADPSRAP	EPETYLFGGPRSMP
$CoiS_A$	SELAQWSFLRK----HPWWRLRYRPACPDSAK	EPEETAFGGPAAMK
WP_225842197.1	DRARNFFFMHK----EPGIRVRFQAVEPGTRQ	EPEDYLFGGPASMT
EPD95946.1	DRARNFFFMHK----EPGIRVRFQAVEPGTRQ	EPEDYLFGGPASMT
WP_114022646.1	DRARNFFFMHK----EPGIRVRFQAVEPGTRQ	EPEHYLFGGPASMT
WP_114017096.1	DRARNFFFMHK----EPGIRVRFQAAEPGEAA	EPEHYLFGGPASMA
WP_030890414.1	GTARNFFFMHK----APGLRVRFQAPGPC-SQ	EPEHYLFGGPGSMR
WP_051846089.1	GTARNFFFMHK----APGLRVRFQAPGPC-PQ	EPEHYLFGGPGSMR
WP_026328419.1	GRARSFYFMHK----EPGLRVRFEAGECS-AA	EPEAYLFGGPQSMP
WP_020930401.1	GRARNFFFMHK----EPGLRVRFEAPDG-ERE	EPEQYLFGGRASMP
WP_044372413.1	GRARNFFFMHK----EPGLRVRFEAPDG-ERE	EPEQYLFGGRASMP
WP_066979273.1	GLARNFFFMHK----EPGLRVRFEAPEEAARE	EAERYLFGGAASMP
WP_050514921.1	RAARNFFFMHK----PPGLRLRFQAPTRDARD	EPETYLFGGARAMP
WP_150247985.1	GAARNFFFMHK----PPGLRLRFQAPARDARD	EPETYLFGGARAMP
WP_180333157.1	GAVRSFFFMHK----PPGLRVRFEAPTPADAR	EPETYLFGGPASMR
WP_063349754.1	GAARSFFFMHK----PPGLRVRFEAGRSD-VG	EPETYLFGGPRSMR
WP_225100191.1	GMVKSFFFMHK----PPGLRARFHATEPSGAA	EPETYLFGGARSME
WP_158928457.1	DLVGSFFFMHK----PPGLRVRFEARGAG-HG	EPETYLFGGPLSMR
MBX7552017.1	GTVRSFLFMHK----PPGLRVRFEAREPE-HG	EPETYLFGGPPSMR
WP_053656156.1	GTVGSFFFMHK----SPGLRVRFEAREPG-HV	EPETYLFGGPRSMR
WP_146478880.1	GPACEFFFMHK----PPGLRIRFQAGRLGDVP	EPEAYLFGGHAAMP
WP_006350662.1	GPACEFFFMHK----PPGLRLRFQARRPGDVS	EPEAYLFGGPASMP
WP_148835526.1	GLAQAFFFMHK----PPGLRVRFQAPGAEGVV	EPESYLFGGPAAMP
WP_093548547.1	RTARAFFFMHK----PPGLRMRFQAYGADGTV	EPESYLFGGPGSMP
WP_124717586.1	HTARAFFFMHK----APGLRVRFQASRPAGTV	EPESYLFGGPGSMP
WP_031128117.1	GAAGDFFFVHK----PPGLRVRFHAPGPDGAD	EPESYLYGGARSMA
WP_069975440.1	GAAGDFFFVHK----PPGLRVRFHAPGPDGAD	EPESYLYGGARSMA
WP_023589891.1	GAADDFFFVHK----PPGLRVRFHAPGPDGAD	EPESYLYGGARSMA
WP_189537590.1	GAADDFFFLHK----PPGLRVRFHAPGPAGAA	EPETYLFGGARSME
WP_223284132.1	GQASNFFFMHK----PPGLRVRFQAADPDQTA	EPETYLFGGPASMP
WP_156695813.1	GWAGDFFFMHK----PPGLRVRFHATRPAGVA	EPETYLFGGVTAMP
WP_126886904.1	GWAHDFFFMHK----PPGLRIRFHAPRPAGVA	EPETYLFGGPRAMP
WP_084904936.1	GWADDFFFMHK----PPGLRIRFHAPRPAGVA	EPETYLFGGPRAMP
WP_063835479.1	GLARDFFFMHK----PPGLRVRFHASEPSRAG	EPETYLFGGPRSMP
WP_037854959.1	GAARGFFFMHK----PPGLRIRFHAAEPARAP	EPETYLFGGPRSMA
WP_064536949.1	GPARDFFFMHK----PPGLRVRFHASHPSRAG	EPETYLFGGPRSMP
WP_037890514.1	GPARDFFFMHK----PPGLRVRFRAAHPSRAG	EPETYLFGGPRSMP
WP_018569787.1	GPARDFFFMHK----PPGLRVRFRAAHPSRAG	EPETYLFGGPRSMP
WP_189302509.1	GQAREFFFMHK----PPGLRVRFLAARPSRAG	EPETYLFGGPPAMP
WP_189798844.1	GPAGDFFFMHK----PPGLRIRFRAADPSRAP	EPETYLFGGPRAMP
GHF94062.1	GPAGDFFFMHK----PPGLRIRFRAADPSRAP	EPETYLFGGPRAMP
WP_125497353.1	GRAGDFFFMHK----PPGLRIRFRAADRSRVP	EPETYLFGGPRAMS
WP_206346003.1	GRAGDFFFMHK----PPGLRIRFRAADRSRVP	EPETYLFGGPRAMS
WP_185948383.1	GQARDFFFMHK----PPGLRVRFRAADPARVQ	EPETYLFGGPRSMP
WP_031097963.1	GQAGDFFFMHK----PPGLRVRFRAAEPARAE	EPETYLFGGPRSMP
WP_053212510.1	GQARDFFFMHK----PPGLRVRFRAAEPARAE	EPETYLFGGPRSMP
WP_030991581.1	GQARDFFFMHK----SPGLRVRFRAAEPARAE	EPETYLFGGPRSMP
WP_196942623.1	GQARDFFFMHK----PPGLRVRFRAAEPARAE	EPETYLFGGPRSMP
WP_030747650.1	GQARDFFFMHK----PPGLRVRFRAAEPGRAP	EPETYLFGGPRSMP
WP_030602278.1	GQARDFFFMHK----PPGLRVRFLAAEPSRAP	EPETYLFGGPLSMP
WP_189991173.1	GQARDFFFMHK----PPGLRVRFLAAEPSRAP	EPETYLFGGPRSMP
WP_189753203.1	GQARDFFFMHK----PPGLRVRFLAAEPSRAP	EPETYLFGGPRSMP
WP_020518774.1	GAAANFFFMHK----PPGLRVRFETSSA-LAD	EPEEALFGGPSSMR
WP_073487574.1	GLAVNFFFMHK----PPGLRLRFQAAPG-RFT	EPETQLFGGPVSMR
WP_189052784.1	GLATNFFFMHK----PPGLRVRFQAAPD-RYE	EPEANLFGGPASMR
WP_025360171.1	GEVTDFFFMHK----PPGLRVRFAAAQG-RAA	EPEAHLFGGPASMR
MBA8925489.1	GEVTDFFFMHK----PPGLRVRFAAAQG-RAA	EPEAHLFGGPASMR
WP_179771686.1	QEIDDFFVMHK----PPGLRVRFAPAPG-HGG	EPEAHLFGGPASMA

Figure S12. Partial sequence alignment of SptB$_b$ homologs identified using RODEO[24] with NisB and MibB focusing on residues that are important for catalysis. A conserved Lys residue (yellow) is found in all homologs but is absent in NisB and MibB. Conversely, conserved Arg residues (blue) that bind to γ-carboxylate of glutamylated peptide are found in both groups of enzymes. Finally, a conserved Arg residue in NisB and MibB that is responsible for acidifying the alpha proton for elimination is replaced by a conserved Leu residue (gray) in SptB$_b$ and its homologs. CoiS$_{A(ED)}$ was added to the alignment separately and contains an Ala at this position.

Glutamyl Lyase SSN

SSN for glutamyl lyases was generated using EFI-EST analysis. Pfam PF14028 (Lant_dehydr_C) was used as query using UniRef90 and UniRef50 sequences. Alignment score threshold was set to 40. SSN was visualized in representative node networks set at 75% identity resulting in 3,831 nodes and 879,407 edges. Each cluster was selected and further subjected to EFI-GNT analysis of the BGCs.

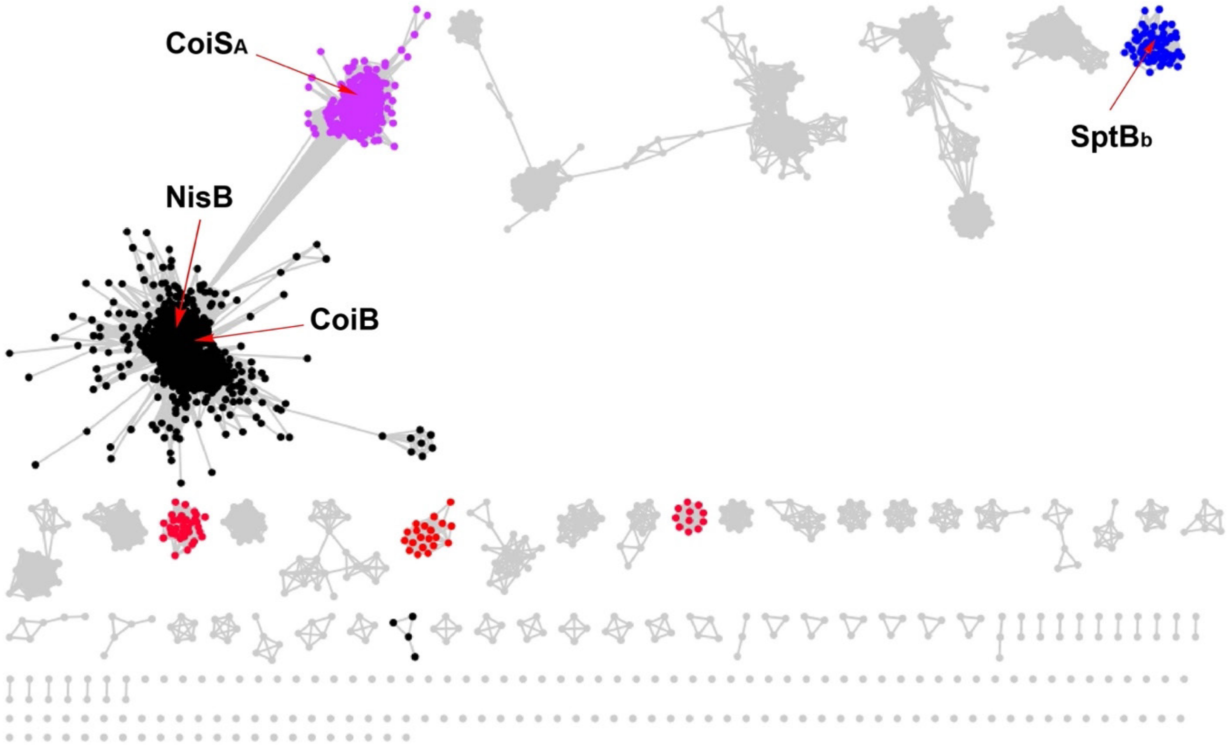

Figure S13. SSN of glutamyl lyases (GL). Sequences surrounding the genes for the glutamyl lyases were manually inspected by EFI-GNT analysis. Black = BGCs that contain full length class I lanthipeptide dehydratases, purple = BGCs encoding two GLs, red = BGCs encoding glutamyl lyases as separate domains from the glutamylation domain (split LanBs), blue = BGCs encoding GLs as separate domains from glutamylation domains and/or containing two GLs. Thiopeptide GLs are not annotated.

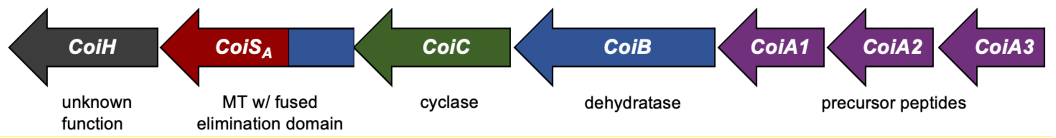

Figure S14. Coi BGC from *S. coelicolor* A3(2) featuring two elimination domains (blue).

Coi BGC DNA Sequences and Molecular Cloning

The synthetic genes for CoiA1, CoiB, CoiC, and CoiS_{A} were codon-optimized for *E. coli* expression (**Table S4**). They were amplified using the primers listed in **Table S5** to introduce 5′ overhangs and allow subsequent Gibson Assembly. For pRSFDuet-1-*coiA1-coiB*, *coiA1* was first cloned in MCS1 of pRSFDuet-1 that was digested with EcoRI and HindIII, producing pRSFDuet-1-*coiA1*. This procedure introduced an N-terminal His_6-tag in CoiA1. The *coiB* gene was then cloned in MCS2 after digestion of pRSFDuet-1-*coiA1* with NdeI and XhoI. A SUMO tag was then inserted between the His_6-tag and CoiA1 by utilizing primers with overhangs via Gibson Assembly (**Table S6**). For pETDuet-1-*coiC-coiS*$_A$, *coiC* was first cloned in MCS1 of pETDuet-1 that was digested with NcoI and HindIII, yielding pETDuet-1-*coiC*. The *coiS*$_A$ gene was then cloned in MCS2 of the NdeI- and XhoI-digested pETDuet-1-*coiC* plasmid. Site-directed mutagenesis using Q5 polymerase was performed to place a stop codon after the elimination domain of CoiS_{A} to generate pETDuet-1 CoiC_$\text{CoiS}_{\text{A(ED)}}$ (**Table S7**). Another round of SDM generated pETDuet-1 CoiC_$\text{CoiS}_{\text{A(ED)}}$ K46A (**Table S7**).

Table S4. Sequences of *E. coli* codon-optimized genes of the *coi* BGC used in this study.

Gene Name	Sequence (5′-3′)
coiA1	ATGAATGCTAACACCATTAAAGGTCAGGCACATTCGCCAGCAGCTACCGCCGGGGG TGATGCCTTTGACCTGGATATCTCAGTTTTAGAAAGTGACGACGGCTCTGCGACCCT GATTAACTTGACGGATGATGGATGTGGCTCCACTTGCAGCAGCCCGTGCGCGACAA ATGTGGCGTAA
coiB	ATGGGGAGCCGAAAAGATTGCCCCTTGTATCGGTGCGCCGACACTGCCCTGGTAAG GGCTGCCAGAAGCGCGCGACTACCCCTGCCTGCATGGCCCGACCTGACAGATGATA CACCGGATTGTGAAGTTCGCTGGCAGACATGGCTGCGTGATGTTTGGAGTTTAAGT GAAGCAGCCGACTCAATTGAACAAGCCTCACCCCTATTGGCACAGCAAGTGCAGGC GCTATGCTCTGTCGCGTCGCCTGAGACGCGTCAGCTACGCCGTGCGGTAGTCTCCGT AATGCGGTACCTGCTTCGCATGACTGGACGGGCGACTCCGAATGGGCTCTTTGCCG GCATCGCTCCCGCATCTTTTGGCGAACGACCCGGTTGGAGCTGGGGTGAATGGCAT CGGCCGGTTATACGAGCTGATGGTGATTGGATAGCCGATTTGGTCGCACGATTGGA GGCTGACCCTGAGTTACTGAGACACCTTCATGTAATGGCCAACAACACCATTCTCAT CAGGGGGGATCGGCTCATCATGCCATACCCACCGCGTTCCTGTAGAACTGATGGGT CCCCTGCAGCCGAAGTATCCGTGCGCTACACGAGCGCCGTGCGGATCGCAGTGGAT GCCGCTGCTTCACCCGTACCTGTCCATGCAGTAGTTGCGCTTATGCGCAGCGAGTTT CCAGACGTGCCCGCTGATCGCGTAGAGGGGCTAGTAAGCTCTCTGGTCGAGCGGG GTGTTCTGATCAGCTCTTTGCATGCACCGTCAGCGACATTGGACGCGCTTGATCACC TTGTAGAACAGGCTGAGGCTGCAGCAGCACCCAGCGCTACGGGGCTGGTGGCGGA

	TCTGCGAACGGTTCGGGATGCAATCGCACGTCATAATCAGGTTCTGGCACCTGCTGA<br>TGGTCGGCGTCTCCGCACCGCTCTCCGTCGCAAAATGACTGCGCTCTCGTCAGTTAA<br>GGCCCAACCGTTCACGCTGGATCTTCGAATGGACTGCAGTCTTACTCTGCCGCCGCA<br>AGTCGCGCGTGAAGCTGAGCAAGCAGCTACAGTACTTGCACGTTTGTCTGCATATCC<br>GTTTGGTACGCCGGCGTGGAAAGATTTTCATAACAGATTTTTCGAGCGCTATGGGAT<br>CAACTCACTTGTCCCTGTCCGGGATTTAGTTGATCCGGATGTGGGGCTGGGCTTCCC<br>AGTTGGGTATCGCGATGCACAACCTGAGCAAGGAGAAGCTCTGACCGCCCGCGAAC<br>AGCGCCTGCTGTCGCTCGCCCAGGCAGCAGTGTTAGACGGACGTGACGAGATCCAT<br>TTGGATGAAGACCTTATCCGTACGTTAACCGTGGGCGACCGCAATGCGCCGCACGT<br>TCCGGCCCATACCGAACTCCGCTTTCGTATTCGCGCCACGAGCCAAGAAGCTCTCGA<br>ATCGGGAGATTTTGTCCTTCATGGTATTGCACCAAGCCGTGGTATCGGCACGACTAT<br>TGGTCGTTCCTTAGGCTTACTGGCGCCTGATGAGCAGGCACGCGTGGCGGTTATGC<br>TTGAGGAGTTACCAGTCAATACTCCTGGTGCCGTACCCACCCAAGTCTCATTTACTCCGTTGG<br>ACCGTGGTGACACTAACGTCACTCGTGTTACTGAGCTGTTGCCAGCAGTAATCAGTCTGGCCGAACAT<br>CGCCCAGTGGACGCCCGCACAATTCCTTTAGACGATCTGGCGGTGGGCTGCGATCGTCGTCGCTTGTA<br>TTTGGTTAGCCTTTCACGTGCGTGCCTGTTGGACCCCATGACTTTGCATGCGCTTGATTTACGCGGACA<br>TACTCCTCCCTTGGGTCGTTTCTTAACAGAGATTTCACGCGCCCAGACGGCTGTGTTAACGGTCTTCCC<br>TTGGGCCAGTGCTACGGCTCTGCCGTATCTGCCCCGCGTTCGTTACGGACGCGCCATTTTGAGCCCCG<br>CGCGCTGGCGTTTAGATCGCTCTGAACTTCCGGATCGTCGCGCTTCCTGGGGAGAGTGGCATAAAGCC<br>GCTGACGAGTGGCGCGCGCCGCCGTGTGCCGGATGAAGTAGCAGTTGCAGAAGGTGACCAGTTA<br>TTACCCTTAGACCTGTCGGAGCGTGCACACTTGGCACTGCTTCGTGCCCACTTAGATACGCATGAGAGT<br>GTAATCCTGACTGAAGCGGAGTCTGAAGAAGGTTGGTTCGATGGACGCTCCCACGAGATCATCGCAC<br>CAATGACAGCTGTGCGCCCCCCCAATGGCCAGCCGTCCCGCCGGTCACGGCAGATCAGCTGGTTACT<br>CGCGACCACGGCTATCTGCCAGGTGCGGCGTCATGGTTATTGGTTAAGTTATACGGACACGTTGATCG<br>CCAACCAGAGATTTTAGCCGACCACTTACCGGCGCTTCTTGGTCAGTGGGACGAACCCCCTACGTGGT<br>GGTACATGCGCTACCGTGATCCACGTTGGCATTTGCGTTTGCGCATTGCCGTGCCAAGTGAGCAAGAT<br>TTCGCATTGACCGCGCGTCGTGTCTCTTCCTGGGCCAACGGCTTACGCCGTGCGGGATTATTGGCAGA<br>TATGCAATTTGCAACTTCTTACCCCGAAACTGGTCGCTGGGGCCCAGGCCCCTTAATGAACTTGGCGG<br>AAGATGTGTTCGCAGCAGATTCGCGCGCACTGGCCGTTCAATTTGCCCAATCTAGCCGCCCACACCAA<br>CAAGTATTAGCCGCGGCTAATTTCGTTAGTGTGGCCGCTGCATTCACCGGCAGTACAGCCGCAGGTAT<br>GAATTGGCTGATCGCACACGGCCGCATCACTGATCCTCGCCCGATGGATCGCACGGTCCGTGGGCAG<br>GCGCTGCGTCTGGCAGACCCAGCAGATGGTTGGGCTGCATTACGTGCCGCCCCCGGTGGCGACGCTA<br>TCGCGAGTGCGTGGTCGGAGCGTGATGCTGCCTTGGCTCGTTACCGTCAGAAACTTGACGCGTTCGGC<br>GGCATCGACTTAGATCTTGTCTTAGACTCATTATTGCACGCCCACCACATTCGCGCCGCCGGTATCGAC<br>AAGGATGATGAGCGTATGTGCGTACGTCTTGCTCATGCCGCCGCTATGGCTTGGACTCACCGCGGTGA<br>TCATCATGAGAGTGCCTAA
coiC	ATGAACCTGAGAGACTCCGCGCTTGCGACCGCAGATACAATAGCCGAGCGGCTGGC<br>CAGCCCGCAAGATATTCGCGAGCTGCATCATCGGCAGGGTTGGTGGCCCCAGTCTT<br>TAGCCCACGGCGCAGCCGGAGTCGCACTCCTACACATTGAGCGGGCCCGCACCGAT<br>CAGGGTCCCTGGCAGAGGGCGCACGACTGGTTGGCGTGCGCAGCGGCCGAACCGG<br>CAGTTGGAGGGCCGGACTCTCACCTGTATTACGGTGCGCCAGCCCTCGCTTTTGCAC<br>TACACGCCGCGGCCGATCGTCCTGGGAGGTATGCCCGAGCTCTAGATACCTTGGAT<br>CGATATGTCACAAAGGCTATTCGTGACCGCTTAGCGGGAGCCCACGCGCGTATGGA<br>CCGTGGTGAGATGCCGGCTTTAGCAGAGTTCGATGCTATTCGCGGACTGTCTGGGA<br>TGGGCGGACTTCTGCTGCATCGTGATGTTCATACGGATGTGCTCCGCGAAGTGCTG<br>ACGTATCTGGTGCGTCTGACCGAACCGGTTAAACACGATGGGGAAGTGTTGCCGGG

	CTGGTGGAGTCATTTAGCCCCAAGTGGCACGATCTCCCCAGAATACCCTGAAGGTCA<br>TGCTAATAACGGCGTTGCACACGGGATTGGCGGCCCACTGGCCGTCCTCAGCCTGG<br>CGGCTCGCCGCGGCATTACTGTGGATGGCCATCTTGATGCTATCACCGGCATTTTGA<br>CCTGGCTGGACCATTGGCAGCAGGACGGCCCGGCCGGCCCGTGGTGGCCCTACTG<br>GATCACTCGTGAACAGCTTCGCTCCGGCACGACTGGTCCTGGCCCGTCCCGGCCAA<br>GCTGGTGCTATGGCACCGCGGGTTTCGCGCGCGTACAGCAACTCGCCGCATTGGCT<br>TTAAAAGATCCTGCCCGTCAACACGCGGCAGAATTTGCCCTGCTGCGGGCCATGAC<br>GGATTCGGGCCAACTGGATGCGACTGTAGACATGTCACTATGCCACGGCTTCGCAG<br>GTCTGGCGCATATCACTCGCCTGGCCGCAGATGATGCGTTAACCCCTGGTCTCACCG<br>CATGTCTGCCGCGCTTGCTGGCACCGATAACGGATACTGCGCCCGGTACCCTGGCG<br>GACTCACTGATCTCGCCCCCCTCGGGAGGTGACATAGGTTTATTGGAAGGTGCAGC<br>AGGCACCGCGTTGGCGTTACATTCATTTCACACTGGTACACCGAGTGCGTCTGGTTG<br>GGAAACCTGTTTTCTTACGAATTAA
$coiS_A$	ATGACCGCCGATGAATGGCCTCAACGTTTGATTCGCTTTACCGATTGGTCACATGCG<br>GAGGCCACTGCCGTTGAACACCTTTTGCCGGTCCTCATCGCCCAAGAAAGTGAGCTC<br>GCACAATGGAGTTTTCTACGGAAGCATCCTTGGTGGCGACTACGCTATCGTCCGGCT<br>TGTCCCGATAGCGCGAAGGCACTCGACGCGGCTCTAGACGAATTAGTGGACGCGG<br>GTGTGTTGGCGAGTTGGACCCGTGGAATATATGAGCCGGAGGAAACGGCGTTTGG<br>GGGGCCGGCGGCCATGAAGATTGCGCATGCCTTGTTCCATTATGACTCGCGCCATCT<br>GTTGGACGAGGCCGCTCGACAGCAAACGGTTAGCGGTCCTCGTCTTGGACGCCGCG<br>AGTTAGCGGTACTCTTACTTTCGGTTGCAATGCGCGCGGCCGGGCTCGATTGGTACG<br>AGCAAGGAGACGTGTGGGCGAGGATAGCCGCAGAACGTTCGGGCGACGAGGTTC<br>GTTCACCACAGCGGCATCGTGCTGCAGTGCATCGCTTAATGACGGTTGATGTGTCTA<br>CGACATCACGGTCAGTCGCGCGCGGCCGTTTAGCACCGCTTGCCGAATGGATTGCG<br>ACGTTTGAGTGGTTTGGACAGCAGCTTGCGGATTTAAATCGACAGGGTCGTCTTGA<br>ACGGGGATTACGCGCGGTAATCGCGCATCATGGGATCTTCCACTTTAATCGCTTGGG<br>TCTGGCAGCGCAGGACCAGCATACCTTGTCGACGCTCGCTAAAGAAGTTGTCATGG<br>GCACAAGTGATAATGCGGCCTCTACGCAGGCAGAAGGGGCTCCAGGAGCAACGGT<br>AAACGGGGTTAATAGCGACACGATTGAAGCCCCCTCGGCGGATCGTCTTCGTGCGC<br>AATTAATTGATCACTTAGTCGAGACAGGTTGTGTACGCACCCCCCGCGTTGAGGAA<br>GCTATGCGTACTGTTCCACGCCACCTGTTCGTACCTAATGCACCCTTGGAGAAAGCA<br>TACGGCAATGCTCCCGTGGATACGAAATTTGATGGAAGTGGTCGCAGCATTAGCTG<br>CGCCAGCCAGCCGGATATTGTTGCAATGATGCTCGAACAATTGGATGTGCAGCCAG<br>GGCAAAAAATCCTGGAGCTGGGCGCCGGCACAGGGTTCAACGCCGGCCTGCTGGG<br>TTATCTGGTGGGCGAAACTGGTCACGTTACCACTATCGATGTGGACGAAGATATTGT<br>CGCGGGCGCACGTGGTGGCCTGGCGGCAGCCGGCATCCACAACGTTGAAGTAATTC<br>TGGGTGATGGTGCGGTGGGTCACACACCGAACGCGCCGTATGACCGTATTGAAGCT<br>ACGGTGGGCGCTCACGGTGTCCCTCACGCATGGCTGGACCAGCTGGCCCCTGGCGG<br>CCGCCTGCTGACACCTCTGCGTCTGCGCGGCTCTGTAAGCCGCTCTATCGCGTTTGA<br>AAATCAAGATGGTGCATGGCGCAGCGTCGGTTCCCAGATGAATACCTTTATGCCGCT<br>GCGCCGTGGCATTGCTGACGATCCAAGACTGTTTGTCCCGCTGGACCCAGATCATAC<br>CGTCACCCTGGTCACCAACGGCGATCAGAAAGTGGATGCAGATGCACTGGGCGATA<br>TTTTCCGGCAGCCAAGAACTGAAGCGTGGACCGATGTGACTTTTCGCGGTCCGGAA

	TCCGCCGAATACCTGGAACTGTGGCTGACCTGCGCCATGCCAAACGGTCTGTCCCGC ATGCCGGCGAAAAACGAAGCGATCGAAAAAGGCCTGGTGACCGCTCCGTATCCGA GTTCCACTGCGGTGTTTGAAGATGGCACCCTGACCTACCTGACCCGTCGTCCGTACG CCAAAAAAGCGCCGGACGGCGCGACCCTGTATGAATTTGGCGTGATCGGTCATGGC CCGGATGCTGAAGCCCTGGCAGGTGATGTGGCCGACCAGATTCGTGCGTGGGATCA GGACTTCCGCGCCCTTGATGTGGGTTTCGAAATTCAGCCGCTGGATGCCGCTCCGCT GGCCCCGAAACCGGGTCGTTTCGCTTTTGATAACCCGCTGAACAGAATCGTGATCGA ATGGCAGTAA

Table S5. Primers Used for Cloning of *coi* Genes

Primer Name	Primer Sequence (5’-3’)
CoiA1-F	ATCACCACAGCCAGGATCCGATGAATGCTAACACCATTAAAGC
CoiA1-R	AAGCATTATGCGGCCGCATTACGCCACATTTGTCGCGC
CoiB-F	AAGTATAAGAAGGAGATATACAATGGGGAGCCGAAAAGATTGC
CoiB-R	GGTGATCATCATGAGAGTGCCTAAGTCTGGTAAAGAAACCGC
CoiB-F2	AGTCAATACTCCTGGTGCCGTACCCACCCAAGTCT
CoiB-R2	GAGGAGTTACCAGTCAATACTCCTGGTGCCGTACCCAC
CoiC-F	GTTTAACTTTAAGAAGGAGATATACATGAACCTGAGAGACTCCGC
CoiC-R	AAGCATTATGCGGCCGCATTAATTCGTAAGAAAACAGG
CoiS$_A$-F	GTTAAGTATAAGAAGGAGATATACAATGACCGCCGATGAATGGCCT
CoiS$_A$-R	GAATCGTGATCGAATGGCAGTAAGTCTGGTAAAGAAACCGCTGCT
CoiS$_A$-F2	CGAAATTTGATGGAAGTGGTCGCAGCATTAGC
CoiS$_A$-R2	ATGCTCCCGTGGATACGAAATTTGATGGAAGTGG

Table S6. Primers Used for insertion of SUMO tag

Primer Name	Primer Sequence (5’-3’)
SUMO-F	TCATCACCACAGCCAGGATCCGGGTTCTTCTATGGCTAGCATGTCGG
SUMO-R	GCCTGACCTTTAATGGTGTTAGCATTCATTCCCTGGAAGTACAGGTTTTCCTC
Backbone-F	GAGGAAAACCTGTACTTCCAGGGAATGAATGCTAACACCATTAAAGGTCAGGC
Backbone-R	CCGACATGCTAGCCATAGAAGAACCCGGATCCTGGCTGTGGTGATGA

Table S7. Primers Used for Site-Directed Mutagenesis of CoiS$_{A(ED)}$.

Primer Name	Primer Sequence (5’-3’)
CoiS$_{A(ED)}$-F	TGTCATGGGCtaaAGTGATAATGC
CoiS$_{A(ED)}$-R	ACTTCTTTAGCGAGCGTC
CoiS$_{A(ED)}$ K46A-F	TTTTCTACGGgccCATCCTTGGTGGC
CoiS$_{A(ED)}$ K46A-R	CTCCATTGTGCGAGCTCA

mCoiA1 Heterologous Production and Analysis

Heterologous Production for the Coi System (general procedure)

Heterologous production of mCoiA1 was performed as described above for mSptA except the plasmids used were the following: pRSFDuet-1 His$_6$-SUMO-CoiA1_CoiB and pETDuet-1

CoiC_CoiS$_{A(ED)}$. His$_6$-SUMO-CoiA1 contains a cleavable TEV protease recognition sequence between SUMO and CoiA1 and the His$_6$-SUMO tag was removed using TEV protease (1:20, peptide: protease) in buffer (20 mM Tris, 500 mM KCl, pH 7.5) to generate mCoiA1. Trifluoroacetic acid was added to a final concentration of 2%, and the sample was centrifuged. The supernatant was collected and subjected to HPLC purification as described above for mSptA. mCoiA1 eluted at 40:60 solvent C:solvent D.

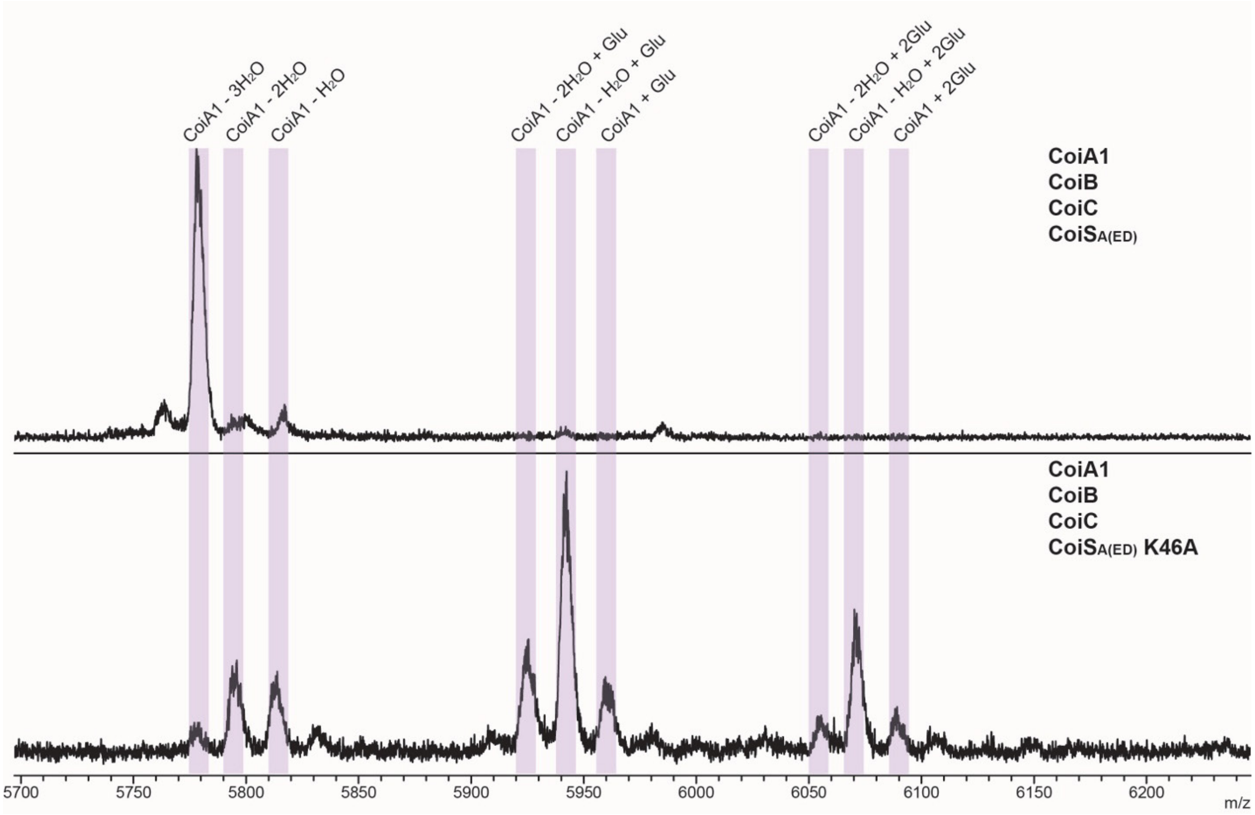

Figure S15. MALDI-TOF MS analysis of mCoiA1 after co-expression with CoiB, CoiC and CoiS$_{A(ED)}$, isolation using Ni-NTA, and treatment with TEV protease. [CoiA1 – 3H$_2$O + H]$^+$ calculated m/z = 5773.6, observed m/z = 5776.2. *E. coli* GluRS/tRNA^{Glu} was sufficient for dehydration activity. CoiA1 sequence after His$_6$-SUMO tag removal using TEV protease: GMNANTIKGQAHSPAATAGGDAFDLDISVLESDDGSATLINLTDDGCGSTCSSPCATNVA.

GC-MS and LC-MS analysis of mCoiA1

Hydrolysis and derivatization procedures to analyze MeLan stereochemistry for mCoiA1 were performed as described above for mSptA.

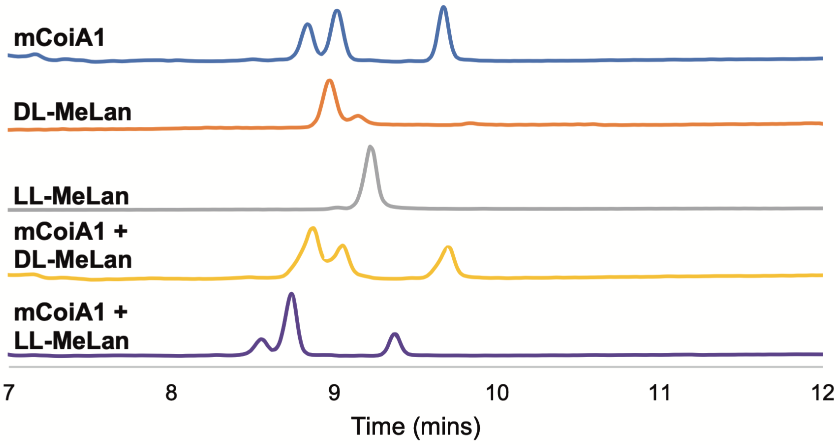

Figure S16. GC-MS analysis of hydrolyzed and derivatized mCoiA1 using a chiral stationary phase and comparison to and coinjections with authentic DL- and LL-MeLan derivatized in the same way. SIM set at m/z = 379. Small differences in retention times are typically observed in these experiments which is why coinjections are used for assignments.

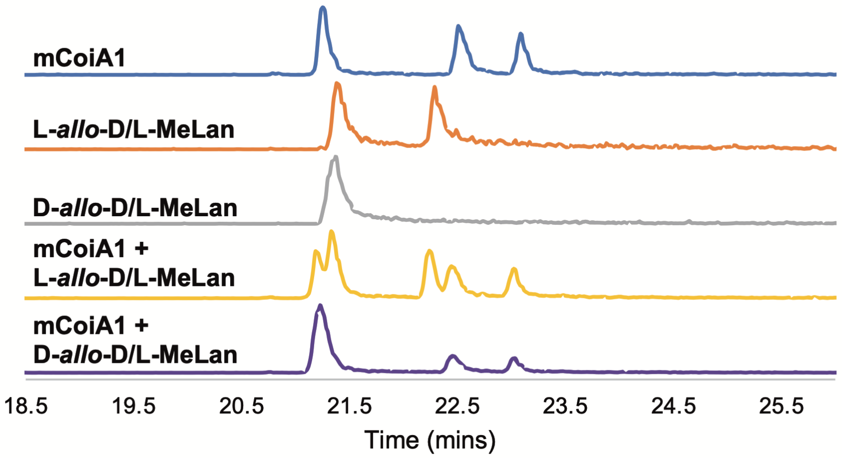

Figure S17. LC-MS analysis of mCoiA1 derivatized with Marfey's reagent and comparison to synthetic L-allo-D/L-MeLan and D-allo-D/L-MeLan derivatized in the same manner. EIC monitored at m/z = 727.1742.

Genome Mining Results

Table S8a. Homologs of the SapT precursor identified via genome mining.

accession number	strain	precursor sequence	leader sequence	core sequence
-	Streptomyces lavenduligriseus Tü901	MPAHEITELDTLISDLEERITDEDLPTASVYTQGCSGLCTIVICATVVICG	MPAHEITELDTLISDLEERITDEDLPTASV	YTQGCSGLCTIVICATVVICG
WP_185948384.1	Streptomyces mexicanus Marseille-Q0842	MPNNELTELDTLISDLEERITDEDLPSASAYTHGCSGVCTIIICNTVVIC	MPNNELTELDTLISDLEERITDEDLPSASA	YTHGCSGVCTIIICNTVVIC
WP_158727391.1	Streptomyces sp. NRRL S-31	MPGNELTELDTLIGDLEERITAEDLPTVSAYTQGCSGLCTILVCGTVVVC	MPGNELTELDTLIGDLEERITAEDLPTVSA	YTQGCSGLCTILVCGTVVVC
WP_189991176.1	Streptomyces achromogenes subsp. rubradiris JCM 4955	MPAHEITELDTLISDLEERITDEELPSADGYSEGCSGLCTIVICATVIICG	MPAHEITELDTLISDLEERITDEELPSADG	YSEGCSGLCTIVICATVIICG

WP_167345722.1	Streptomyces sp. DSM 40868	MPAHEITELDTLISDLEERITDEDLPTASVYTQGCSGLCTIVICATVVICG	MPAHEITELDTLISDLEERITDEDLPTASV	YTQGCSGLCTIVICATVVICG
WP_189798846.1	Streptomyces thermodiastaticus JCM 4840	MPDNELTELDTLISDLEQRITDEDLPSASAYTEGCSGLCTIIICNTVVIC	MPDNELTELDTLISDLEQRITDEDLPSASA	YTEGCSGLCTIIICNTVVIC
WP_189753201.1	Streptomyces eurythermus JCM 4206	MPAHEITELDTLISDLEERITDEELPTASVYTQGCSGLCTIVICATVVICG	MPAHEITELDTLISDLEERITDEELPTASV	YTQGCSGLCTIVICATVVICG
WP_167346007.1	Streptomyces achromogenes subsp. achromogenes NRRL B-2120	MPAHEINELDTLISDLEERITDEDLPTASVYTNGCSGLCTIVICATVVICG	MPAHEINELDTLISDLEERITDEDLPTASV	YTNGCSGLCTIVICATVVICG
WP_168132482.1	Streptomyces thermoviolaceus subsp. apingens JCM 4312	MPTNELTELDTLISDLEERITDEDLPSASAYSEGCSGLCTIIICNTVIVC	MPTNELTELDTLISDLEERITDEDLPSASA	YSEGCSGLCTIIICNTVIVC
WP_189302513.1	Streptomyces cinerochromogenes JCM 3385	MPANELTELDTLISDLEERITSEDLPSASAYTEGCSGLCTILVCATVVIC	MPANELTELDTLISDLEERITSEDLPSASA	YTEGCSGLCTILVCATVVIC
WP_167357625.1	Streptomyces sp. MC1	MPDKELTELDTLISDLEERITSEDLPSVSAYTEGCSGLCTIVVCNTVVIC	MPDKELTELDTLISDLEERITSEDLPSVSA	YTEGCSGLCTIVVCNTVVIC
WP_199835893.1	Streptomyces sp. NRRL S-1896	MPDKERTELDTLISDLEERITSEDLPSVSAYTEGCSGLCTIVVCNTVVIC	MPDKERTELDTLISDLEERITSEDLPSVSA	YTEGCSGLCTIVVCNTVVIC
WP_189397488.1	Streptomyces sp. FBKL.4005	MNHNELTELDTLISDLEERITHDDLPATAAYTEGCSGLCTIVVCGTVVIC	MNHNELTELDTLISDLEERITHDDLPATAA	YTEGCSGLCTIVVCGTVVIC
WP_158771544.1	Streptomyces sp. NRRL S-340	MPNTELTELDALISDLDERISDSDLPSASASTAGCSGLCTIIVCATVVICA	MPNTELTELDALISDLDERISDSDLPSASA	STAGCSGLCTIIVCATVVICA
WP_018569785.1	Streptomyces sp. PsTaAH-124	MPHTELTELDALISDLDERISDSDLPSAAASTSGCSGLCTIIVCATVVICA	MPHTELTELDALISDLDERISDSDLPSAAA	STSGCSGLCTIIVCATVVICA
WP_173860935.1	Streptomyces sp. SAT1	MPHTELTELDALISDLDERISDSDLPSAAASTVGCSGLCTIIVCATVVICA	MPHTELTELDALISDLDERISDSDLPSAAA	STVGCSGLCTIIVCATVVICA
WP_159048675.1	Streptomyces sp. NRRL F-4489	MPTIELTELDTLIDDLDARITESELPSAEAYTEACSGVCTVIICNTAVVCL	MPTIELTELDTLIDDLDARITESELPSAEA	YTEACSGVCTVIICNTAVVCL
WP_189396911.1	Streptomyces sp. FBKL.4005	MNHNEATELDTLINDLEERITHDDLPATAAYTEGCSGLCTIVICGTVVIC	MNHNEATELDTLINDLEERITHDDLPATAA	YTEGCSGLCTIVICGTVVIC
WP_020930403.1	Streptomyces albulus CCRC 11814	MPTTIELTDLDNLIGDLDARITESELPSAEAYTGGCSGVCTVIVCDTVLFCSGVVC	MPTTIELTDLDNLIGDLDARITESELPSAEA	YTGGCSGVCTVIVCDTVLFCSGVVC
WP_180333155.1	Streptomyces sp. NEAU-sy36	MPVSTEFTDLDALIGDLDERITESDLPTAQAHTMECSGVCTVIVCATVVIC	MPVSTEFTDLDALIGDLDERITESDLPTAQA	HTMECSGVCTVIVCATVVIC
WP_199272971.1	Streptomyces broussonetiae T44	MPEIMQLSDLDALIGDLETRITESTLPSAEADTGGCSGVCTVIVCDTVVVCSGVVC	MPEIMQLSDLDALIGDLETRITESTLPSAEA	DTGGCSGVCTVIVCDTVVVCSGVVC
WP_199829507.1	Streptomyces sp. MMG1121	MPETMQLSDLDTLIGDLEARITESTLPTPEADSSGCSGVCTVIVCDTAVVCSGVIC	MPETMQLSDLDTLIGDLEARITESTLPTPEA	DSSGCSGVCTVIVCDTAVVCSGVIC
WP_187438531.1	Streptomyces sp. sk2.1	MPTMELTDLDNLITELDERITGAELPTMDGGLGDTGGCSGLCTVLVCSVIGVC	MPTMELTDLDNLITELDERITGAELPTMDGGLG	DTGGCSGLCTVLVCSVIGVC
WP_198944109.1	Streptomyces sp. CB03234	MPGTMELTDLDTLIDELDERITDSELPAEQAYTAGCSGVCTIVICTAIVC	MPGTMELTDLDTLIDELDERITDSELPAEQA	YTAGCSGVCTIVICTAIVC
WP_189992690.1	Streptomyces achromogenes subsp. rubradiris JCM 4955	MPETMQLTDLDALIGDLETRITEDTLLPSGAYTEGCSGVCTVIVCDTVVICSGIGC	MPETMQLTDLDALIGDLETRITEDTLLPSGA	YTEGCSGVCTVIVCDTVVICSGIGC
WP_167745873.1	Streptomyces sp. MJM8645	MPDITALTDLDALIGDLDARITETTLPTPEASSGGCSGVCTVLICDTALLCSGLFC	MPDITALTDLDALIGDLDARITETTLPTPEA	SSGGCSGVCTVLICDTALLCSGLFC
WP_016468357.1	Streptomyces albus NBRC 13014	MPNGKTEFELDALIDDLDARVTESELAVSENTGAYSQVCSIVVCNTVVICG	MPNGKTEFELDALIDDLDARVTESELAVSENTGA	YSQVCSIVVCNTVVICG
WP_168220613.1	Streptomyces sp. RFCAC02	MPNNDITELDTLISELDERITESELPEANGGGSVLCTIIVCNSVLCA	MPNNDITELDTLISELDERITESELPEANGG	GSVLCTIIVCNSVLCA
WP_202516319.1	Streptomyces sp. SID486	MPDIMELDDLDALIGDLDARISESTLPTAEADTAHCSGVCTILVCTVIVC	MPDIMELDDLDALIGDLDARISESTLPTAEA	DTAHCSGVCTILVCTVIVC
WP_167538468.1	Streptomyces albofaciens JCM 4342	MSDTMERTELTDLDELIGDLETRITETDLPETEADSGHCSGVCTVLVCTIAVC	MSDTMERTELTDLDELIGDLETRITETDLPETEA	DSGHCSGVCTVLVCTIAVC
WP_016907886.1	Streptomyces xiaopingdaonensis DUT 180	MPPTLGTAELDALVEELDARITETDLPEGSADTYECSGACTVLICTVVVC	MPPTLGTAELDALVEELDARITETDLPEGSA	DTYECSGACTVLICTVVVC
WP_169794622.1	Streptomyces albus NRRL B-2465	MPNGKTEFELDALIDDLDARVTESELAVSENTGAYSQVCSIVICNTVVICS	MPNGKTEFELDALIDDLDARVTESELAVSENTGA	YSQVCSIVICNTVVICS
WP_168440871.1	Streptomyces somaliensis DSM 40738	MPDTLELSDLDALIDGLDERITETELPEAQATSETCSLLCTIIVCASAVCV	MPDTLELSDLDALIDGLDERITETELPEAQA	TSETCSLLCTIIVCASAVCV
WP_177235224.1	Streptomyces sp. MUSC 14	MPPTVELTDLDALIGDLDARITETAVPSAQADTGGCSGVCTVVVCDTVVVCSGFIC	MPPTVELTDLDALIGDLDARITETAVPSAQA	DTGGCSGVCTVVVCDTVVVCSGFIC
WP_019546720.1	Streptomyces sulphureus DSM 40104	MPPTIGTAELDALIEELDARITETDLTEGSADTYECSGACTVMVCTVIVC	MPPTIGTAELDALIEELDARITETDLTEGSA	DTYECSGACTVMVCTVIVC
WP_199819870.1	Streptomyces sp. NRRL S-118	MPKIELADLDSLIDDLDERITDSDLPSASPSTAGCSGLCTIIVCATVVIC	MPKIELADLDSLIDDLDERITDSDLPSASP	STAGCSGLCTIIVCATVVIC
WP_156695815.1	Streptomyces ficellus NRRL 8067	MPGTIELTDLDALIDALDERITDTELPATQTMSQVCTVIVCGTVVICA	MPGTIELTDLDALIDALDERITDTELPATQT	MSQVCTVIVCGTVVICA
WP_164495200.1	Streptomyces sp. ADI95-17	MPIIELTELDTLIDELDERITGSDLPTVEASDSLACTIIICNSTLCGSALNC	MPIIELTELDTLIDELDERITGSDLPTVEAS	DSLACTIIICNSTLCGSALNC
WP_023589889.1	Streptomyces thermolilacinus SPC6	MPQTLELTDLDSLIDDLDTRITETELPDAQAYTAGCSGLCTIIVCATVVICA	MPQTLELTDLDSLIDDLDTRITETELPDAQA	YTAGCSGLCTIIVCATVVICA

WP_176729330.1	Streptomyces sp. DvalAA-43	MSVMEITDLDTLIDELDERITSSDLPAVEASDSLLCATVLICGGSTICGSAINC	MSVMEITDLDTLIDELDERITSSDLPAVEAS	DSLLCATVLICGGSTICGSAINC
WP_199787167.1	Streptomyces sp. CNT360	MPATIEAPEVDLDSLIDELDTRISSTTELPDARMDTGGCSGLCTVLVCTVIVC	MPATIEAPEVDLDSLIDELDTRISSTTELPDARM	DTGGCSGLCTVLVCTVIVC
WP_162887729.1	Streptomyces fradiaeATCC 10745	MPQTLELTDLDSLIDDLDTRITETELPDAQAATAACSGLCTIIICATVIICA	MPQTLELTDLDSLIDDLDTRITETELPDAQA	ATAACSGLCTIIICATVIICA

Table S8b. Homologs of the SapB precursor identified via genome mining.

accession number	strain	precursor sequence	leader sequence	core sequence
-	Streptomyces coelicolor	MNLFDLQSMETPKEEAMGDVETGSRASLLLCGDSSLSITTCN	MNLFDLQSMETPKEEAMGDVE	TGSRASLLLCGDSSLSITTCN
GGQ92051.1	Streptomyces flaveolus	MNKHPLSVRRDEHMNLFDLQSMETPKEEAMGDVETGSRASLLLCGDSSLSITTCN	MNKHPLSVRRDEHMNLFDLQSMETPKEEAMGDVE	TGSRASLLLCGDSSLSITTCN
WP_156699659.1	Streptomyces sp. Z38	MNLFDLQSMETPKEEAVGDVETGSRASLLLCGDSSLSVTTCN	MNLFDLQSMETPKEEAVGDVE	TGSRASLLLCGDSSLSVTTCN
WP_189705834.1	Streptomyces anandii	MNLFDLQSMETPKEEAMGDVEHGSRASLLLCGDSSLSVTTCN	MNLFDLQSMETPKEEAMGDVE	HGSRASLLLCGDSSLSVTTCN
MQL65438.1	Streptomyces vinaceus	MNLFDLQSMETPKEEAVGDVETGSRASLLLCGDSSLSITTCN	MNLFDLQSMETPKEEAVGDVE	TGSRASLLLCGDSSLSITTCN
WP_006130296.1	Streptomyces rubiginosus JCM 4416	MNLFDLQSMETPKEEAMGDVETGSRASLLLCGDSSLSVTTCN	MNLFDLQSMETPKEEAMGDVE	TGSRASLLLCGDSSLSVTTCN
WP_053556909.1	Streptomyces zhihengii	MTLLDLQSMETPKEEAIGDVETGSRASLLLCGDSSLSVTTCN	MTLLDLQSMETPKEEAIGDVE	TGSRASLLLCGDSSLSVTTCN
WP_015037662.1	Streptomyces parvulus	MNLFDLQSLETPKDEAIGDVETGSRASLLLCGDSSLSVTTCN	MNLFDLQSLETPKDEAIGDVE	TGSRASLLLCGDSSLSVTTCN
WP_190179166.1	Streptomyces naganishii	MNLFDLQSMETPKEEAMGDVENGSRASLLLCGDSSLSVTTCH	MNLFDLQSMETPKEEAMGDVE	NGSRASLLLCGDSSLSVTTCH
WP_030855946.1	Streptomyces sp. NRRL S-37	MNLLDLQSMETPKEEAMGDVETGSRASLLLCGDSSLSVTTCN	MNLLDLQSMETPKEEAMGDVE	TGSRASLLLCGDSSLSVTTCN
WP_030325150.1	Streptomyces flavochromogenes	MNLFDLQSLETPKDEAIGDVETGSRASLLLCGDSSLSITTCN	MNLFDLQSLETPKDEAIGDVE	TGSRASLLLCGDSSLSITTCN
WP_046498341.1	Streptomyces odonnellii	MNLLDLQSLEIPKEEAAGDVATGSRASLLLCGDSSLSVVTCN	MNLLDLQSLEIPKEEAAGDVA	TGSRASLLLCGDSSLSVVTCN
WP_023536623.1	Streptomyces niveus	MNLLDLQSMETPKEEATGDVATGSRASLLLCGDSSLSITTCN	MNLLDLQSMETPKEEATGDVA	TGSRASLLLCGDSSLSITTCN
WP_189530606.1	Streptomyces roseolilacinus	MTLLDLQSMETPKEEAAGDVATGSRASLLLCGDSSLSVTTCN	MTLLDLQSMETPKEEAAGDVAN	TGSRASLLLCGDSSLSVTTC
WP_149551904.1	Streptomyces pilosus	MNLFDLQSMETPKEEAMGDVESGSRASLLLCGDSSLSVTTCN	MNLFDLQSMETPKEEAMGDVE	SGSRASLLLCGDSSLSVTTCN
EFE83147.1	Streptomyces albidoflavus	MAVVRPLDTKEYVMALLDLQAMDTPQEEAVGDLATGSQISLLICEYSSLSVTLCTP	MAVVRPLDTKEYVMALLDLQAMDTPQEEAVGDLA	TGSQISLLICEYSSLSVTLCTP
WP_073755677.1	Streptomyces sp. CB03234	MTLLDLQSMETPKEEATGDIATGSRASLLLCGDSSLSVTTCN	MTLLDLQSMETPKEEATGDIA	TGSRASLLLCGDSSLSVTTCN
WP_016827684.1	Streptomyces olivaceus	MNLFDLQSLETPKEEAMGDVETGSRASLLLCGDSSLSVTTCN	MNLFDLQSLETPKEEAMGDVE	TGSRASLLLCGDSSLSVTTCN
WP_058940762.1	Streptomyces kanasensis	MTLLDLQSMETPKEEATGDVATGSRASLLLCGDSSLSVTTCN	MTLLDLQSMETPKEEATGDVA	TGSRASLLLCGDSSLSVTTCN
WP_040905584.1	Streptomyces griseoflavus	MNLFDLQSMETPKEEAMGDVESGSRASLLLCGDSSLSITTCN	MNLFDLQSMETPKEEAMGDVE	SGSRASLLLCGDSSLSITTCN
ESP98942.1	Streptomyces sp. GBA 94-10	MAVARPLDTKEYVMALLDLQAMDTPQEEAVGDLATGSQISLLICEYSSLSVTLCTP	MAVARPLDTKEYVMALLDLQAMDTPQEEAVGDLA	TGSQISLLICEYSSLSVTLCTP
WP_105870550.1	Streptomyces sp. ST5x	MTLLDLQSMETPKDEAMGDVATGSRASLLLCGDSSLSVTTCN	MTLLDLQSMETPKDEAMGDVA	TGSRASLLLCGDSSLSVTTCN
WP_121750866.1	Streptomyces galilaeus	MNLFELQSMETPKEEAMGDVETGSRASLLLCGDSSLSVTTCN	MNLFELQSMETPKEEAMGDVE	TGSRASLLLCGDSSLSVTTCN
MBC7268563.1	Streptomyces sp.	MNLLDLQSMETPKEEAMGDVESGSRASLLLCGDSSLSVTTCN	MNLLDLQSMETPKEEAMGDVE	SGSRASLLLCGDSSLSVTTCN
WP_185029709.1	Streptomyces candidus	MSILDLQSMETPKEEAIGDVESGSRASLLLCGDSSLSITTCN	MSILDLQSMETPKEEAIGDVE	SGSRASLLLCGDSSLSITTCN
WP_156695566.1	Streptomyces ficellus	MSLLDLQSMETPKEEATGDVATGSRASLLLCGDSSLSVTTCN	MSLLDLQSMETPKEEATGDVA	TGSRASLLLCGDSSLSVTTCN
WP_114622539.1	Streptomyces corynorhini	MNLLDLQSLETPKEEAIGDVETGSRASLLLCGNSSLSITTCN	MNLLDLQSLETPKEEAIGDVE	TGSRASLLLCGNSSLSITTCN
WP_030740614.1	Streptomyces sp. NRRL F-5135	MNLLELQSMETPKEEATGDVATGSRASLLLCGDSSLSVTTCN	MNLLELQSMETPKEEATGDVA	TGSRASLLLCGDSSLSVTTCN
WP_041993935.1	Streptomyces sp. AcH 505	MALLDLQAMETPAEEAFGDVATGSQVSLLICEFSSLSVVLCTP	MALLDLQAMETPAEEAFGDVA	TGSQVSLLICEFSSLSVVLCTP
WP_055420103.1	Streptomyces pactum	MNLFEMQSMETPKEEAMGDVETGSRASLLLCGDSSLSVTTCN	MNLFEMQSMETPKEEAMGDVE	TGSRASLLLCGDSSLSVTTCN
WP_161230710.1	Streptomyces sp. SID4917	MTLLDLQSMETPKEEAIGDVETGSRASLLLCGNSSLSLVTCN	MTLLDLQSMETPKEEAIGDVE	TGSRASLLLCGNSSLSLVTCN
WP_028428337.1	Streptomyces sp. TAA040	MTLLDLQSMETPKEETTGDVATGSRASLLLCGDSSLSVTTCN	MTLLDLQSMETPKEETTGDVA	TGSRASLLLCGDSSLSVTTCN
WP_031071299.1	Streptomyces sp. NRRL S-118	MTLLDLQSMETPKEEVTGDVASGSRASLLLCGDSSLSVTTCN	MTLLDLQSMETPKEEVTGDVA	SGSRASLLLCGDSSLSVTTCN
WP_018849360.1	Streptomyces sp. DHE17-7	MNLFELQSLETPKEEAMGDVETGSRASLLLCGDSSLSVTTCN	MNLFELQSLETPKEEAMGDVE	TGSRASLLLCGDSSLSVTTCN
WP_121515790.1	Streptomyces sp. Z26	MNLLDLQTLETPKDELNGEVATGSRASLLLCGDSSLSVTTCN	MNLLDLQTLETPKDELNGEVA	TGSRASLLLCGDSSLSVTTCN
WP_019761740.1	Streptomyces sp. SID4944	MALLDLQAMETPAEENFGELATGSQVSLLVCEYSSLSVVLCTP	MALLDLQAMETPAEENFGELA	TGSQVSLLVCEYSSLSVVLCTP

WP_161328993.1	Streptomyces sp. SID5789	MNLFDLQSMETPKEEAMGDVETGGSRASLLLCGDSSLSVTTCN	MNLFDLQSMETPKEEAMGDVET	GGSRASLLLCGDSSLSVTTCN
WP_189422941.1	Streptomyces niveoruber	MNLFELQSLETPKDEAMGDVETGSRASLLLCGDSSLSITTCN	MNLFELQSLETPKDEAMGDVE	TGSRASLLLCGDSSLSITTCN
WP_066952675.1	Streptomyces lushanensis	MSLLDLQSMETPKEEAIGDVETGSQASLLLCGDSSLSLFTCN	MSLLDLQSMETPKEEAIGDVE	TGSQASLLLCGDSSLSLFTCN
WP_093692443.1	Streptomyces sp. SJ1-7	MALLDLQAMETPAEENFGELATGSQVSLLVCEYSSLSVTLCTP	MALLDLQAMETPAEENFGELA	TGSQVSLLVCEYSSLSVTLCTP
WP_122620605.1	Streptomyces sp. WAC 04229	MNLFDLQSMETPKDEAMGDVETGGSRASLLLCGDSSLSVTTCN	MNLFDLQSMETPKDEAMGDVET	GGSRASLLLCGDSSLSVTTCN
WP_018510165.1	Streptomyces sp. SID5594	MALLDLQAMETPAEEAFGELATGSQVSLLVCEFSSLSVVLCTP	MALLDLQAMETPAEEAFGELA	TGSQVSLLVCEFSSLSVVLCTP
WP_030561171.1	Streptomyces cyaneofuscatus	MALLDLQAMETPAEENFGELATGSQVSLLVCEFSSLSVVLCTP	MALLDLQAMETPAEENFGELA	TGSQVSLLVCEFSSLSVVLCTP
WP_010272359.1	Streptomyces sp. CLI2509	MALLDLQVMETPEEEGFQDIATGSQVSLLICEYSSLSVTLCTP	MALLDLQVMETPEEEGFQDIA	TGSQVSLLICEYSSLSVTLCTP
WP_190126772.1	Streptomyces inusitatus	MALLDLQALETPAEDAFGDVEHGSQVSLLVCEHSSLSVVLCTP	MALLDLQALETPAEDAFGDVE	HGSQVSLLVCEHSSLSVVLCTP
WP_136738041.1	Streptomyces piniterrae	MSLLDLQTMETPKPEATEELHTGSRASLLLCGDSSLSVTTCN	MSLLDLQTMETPKPEATEELH	TGSRASLLLCGDSSLSVTTCN
WP_184821471.1	Streptomyces griseostramineus	MNLFELQSLETPKDEAMGDVESGSRASLLLCGDSSLSVTTCN	MNLFELQSLETPKDEAMGDVE	SGSRASLLLCGDSSLSVTTCN
WP_189936564.1	Streptomyces sulfonofaciens	MNLLDLQSLETPKDEAAGDVATGSQASLLLCGDSSLSVVTC	MNLLDLQSLETPKDEAAGDVA	TGSQASLLLCGDSSLSVVTC
WP_046929993.1	Streptomyces lydicus	MTLLDLQTMETPKPEATDELCHGSRASLLLCGDSSLSVTTCN	MTLLDLQTMETPKPEATDELC	HGSRASLLLCGDSSLSVTTCN
WP_077967437.1	Streptomyces tsukubensis	MALLDLQSMDTPQEEAVGDIATGSQVSLLICEYSSLSVVLCTP	MALLDLQSMDTPQEEAVGDIA	TGSQVSLLICEYSSLSVVLCTP
WP_128507973.1	Streptomyces inhibens	MTLLDLQTMETPKAEATEELHTGSRASLLLCGDSSLSITTCN	MTLLDLQTMETPKAEATEELH	TGSRASLLLCGDSSLSITTCN
WP_168535975.1	Streptomyces physcomitrii	MSLLDLQTMDTPKEEAFGDLATGSQVSLLVCEYSSLSVVLCTP	MSLLDLQTMDTPKEEAFGDLA	TGSQVSLLVCEYSSLSVVLCTP
WP_033265003.1	Streptomyces lydicus	MSLLDLQTMETPKPEGTEELHTGSRASLLLCGDSSLSVTTCN	MSLLDLQTMETPKPEGTEELH	TGSRASLLLCGDSSLSVTTCN
WP_015611153.1	Streptomyces fulvissimus DSM 40593	MALLDLQAMETPAEESFGELATGSQVSLLVCEYSSLSVVLCTP	MALLDLQAMETPAEESFGELA	TGSQVSLLVCEYSSLSVVLCTP
WP_093638117.1	Streptomyces sp. NEAU-S7GS2	MSLLDLQTMETPKAEATEELHTGSRASLLLCGDSSLSVTTCN	MSLLDLQTMETPKAEATEELH	TGSRASLLLCGDSSLSVTTCN
WP_203215277.1	Streptomyces koyangensis	MALLDLQAMDTPQEEAVGDLATGSQVSLLICEYSSLSVTLCTP	MALLDLQAMDTPQEEAVGDLA	TGSQVSLLICEYSSLSVTLCTP
WP_030068093.1	Streptomyces natalensis	MTLLDLQTMETPKAEATEELHTGSRASLLLCGDSSLSITTCH	MTLLDLQTMETPKAEATEELH	TGSRASLLLCGDSSLSITTCH
WP_053925361.1	Streptomyces chattanoogensis	MTLLDLQTMETPKAEATDELCHGSRASLLLCGDSSLSVTTCN	MTLLDLQTMETPKAEATDELC	HGSRASLLLCGDSSLSVTTCN
WP_052869432.1	Streptomyces niger	MALLDLQAMDTPTEEAAGDLAHGSQVSLLICEYSSLSVVACTP	MALLDLQAMDTPTEEAAGDLA	HGSQVSLLICEYSSLSVVACTP
WP_028440635.1	Streptomyces sp. SM18	MALLDLQALDTPAEEAFDEVATGSQVSLLVCEYSSLSVVLCTP	MALLDLQALDTPAEEAFDEVA	TGSQVSLLVCEYSSLSVVLCTP
WP_073722753.1	Streptomyces sp. TSRI0281	MALLDLQAMETPADEAFGELATGSQVSLLICEHSSLSLTLCTP	MALLDLQAMETPADEAFGELA	TGSQVSLLICEHSSLSLTLCTP
WP_124269936.1	Streptomyces sp. ADI96-02	MALLDLQAMETPADETFGELATGSQVSLLICEFSSLSVTLCTP	MALLDLQAMETPADETFGELA	TGSQVSLLICEFSSLSVTLCTP
WP_008415578.1	Streptomyces sp. IgraMP-1	MALLDLQAMDTPQEEAVGDLATGSQISLLICEYSSLSVTLCTP	MALLDLQAMDTPQEEAVGDLA	TGSQISLLICEYSSLSVTLCTP
WP_031123116.1	Streptomyces sp. MBT57	MALLDLQAMETPAEESFGELATGSQVSLLVCEFSSLSVVLCTP	MALLDLQAMETPAEESFGELA	TGSQVSLLVCEFSSLSVVLCTP
WP_018956281.1	Streptomyces sp. TRM68085	MALLDLQAMETPAEDSFGELATGSQVSLLVCEYSSLSVVLCTP	MALLDLQAMETPAEDSFGELA	TGSQVSLLVCEYSSLSVVLCTP
WP_030624260.1	Streptomyces sp. AmelKG-A3	MALLDLQALDTPAEEAFDEVATGSQISLLVCEYSSLSVVLCTP	MALLDLQALDTPAEEAFDEVA	TGSQISLLVCEYSSLSVVLCTP
WP_096626963.1	Streptomyces sp. WZ.A104	MALLDLQAMETPAEDSFGELATGSQVSLLVCEYSSLSVTLCTP	MALLDLQAMETPAEDSFGELA	TGSQVSLLVCEYSSLSVTLCTP
WP_103835860.1	Streptomyces sp. Ru73	MALLDLQAMETPAEDSFGELATGSQVSLLVCEYSSLSVTLCTP	MALLDLQAMETPAEDSFGELA	TGSQVSLLVCEYSSLSVTLCTP
WP_086779712.1	Streptomyces fimicarius	MALLDLQAMDTPAEDTFGELATGSQVSLLVCEYSSLSVVLCTP	MALLDLQAMDTPAEDTFGELA	TGSQVSLLVCEYSSLSVVLCTP
WP_042152795.1	Streptomyces platensis	MSLLDLQTLETPKAEATEELHTGSRASLLLCGDSSLSVTTCN	MSLLDLQTLETPKAEATEELH	TGSRASLLLCGDSSLSVTTCN
WP_109779820.1	Streptomyces sp. CG 926	MTLLDLQSMETPKEETTEVAHGGGGGSRASLLLCGDSSLSITTCN	MTLLDLQSMETPKEETTEVAHGGG	GGSRASLLLCGDSSLSITTCN
WP_103540018.1	Streptomyces sp. SM9	MALLDLQAMDTPQEEAVGDLATGSQISLLICEYSSLSVLLLRGVHRLEVEK	MALLDLQAMDTPQEEAVGDLA	TGSQISLLICEYSSLSVLLLRGVHRL EVEK
WP_040253074.1	Streptomyces albus	MSLLDLQTMDTPQEEAFGDLATGSQVSLLVCEYSSLSVVLCSP	MSLLDLQTMDTPQEEAFGDLA	TGSQVSLLVCEYSSLSVVLCSP
QIK10444.1	Streptomyces sp. ID38640	MSLLDLQTMETPKAEATEELHTGSRASLLLCGDSSLSVSTCN	MSLLDLQTMETPKAEATEELH	TGSRASLLLCGDSSLSVSTCN
WP_030723876.1	Streptomyces sp. Mg1	MTLLDLQSMETPKEETTEAAHGGGGGSRASLLLCGDSSLSITTCN	MTLLDLQSMETPKEETTEAAHGGG	GGSRASLLLCGDSSLSITTCN
WP_003966507.1	Streptomyces sp. SID4931	MALLDLQAMDTPAEDSFGELATGSQVSLLVCEYSSLSVVLCTP	MALLDLQAMDTPAEDSFGELA	TGSQVSLLVCEYSSLSVVLCTP
Q07642.1	Streptomyces griseus	MALLDLQAMDTPAEDSFGELRTGSQVSLLVCEYSSLSVVLCTP	MALLDLQAMDTPAEDSFGELR	TGSQVSLLVCEYSSLSVVLCTP
KPI02674.1	Actinobacteria bacterium OV450	MTLLDLQSMETPKEETTEAAMTGSRASLLLCGDSSLSVTTCN	MTLLDLQSMETPKEETTEAAM	TGSRASLLLCGDSSLSVTTCN

WP_073215017.1	Streptomyces sp. NBRC 110465	MALLDLQAMDTPAEDSFGELATGSQVSLLVCEYSSLSVTLCTP	MALLDLQAMDTPAEDSFGELA	TGSQVSLLVCEYSSLSVTLCTP
WP_079431206.1	Streptomyces sp. Ag109_G2-1	MTLLDLQSMETPKEENTEAAHLHGGGSRASLLLCGDSSLSVTTCN	MTLLDLQSMETPKEENTEAAHLHG	GGSRASLLLCGDSSLSVTTCN
WP_030112254.1	Streptomyces globisporus	MALLDLQAMDTPAEDSFGELATGSQVSVLVCEYSSLSVVLCTP	MALLDLQAMDTPAEDSFGELA	TGSQVSVLVCEYSSLSVVLCTP
MBL0780078.1	Streptomyces albidoflavus	MALLDLQAMGTPQEEAVGDLATGSQISLLICEYSSLSVTLCTP	MALLDLQAMGTPQEEAVGDLA	TGSQISLLICEYSSLSVTLCTP
WP_030389872.1	Streptomyces sp. Sge12	MTLLDLQSMDTPKEETTEAAHGGGGGSRASLLLCGDSSLSITTCN	MTLLDLQSMDTPKEETTEAAHGGG	GGSRASLLLCGDSSLSITTCN
WP_023530017.1	Streptomyces sp. RKND-216	MSLLDLQTMETPKDEAIGDLETGGGSRASLLLCGDSSLSLTTCN	MSLLDLQTMETPKDEAIGDLETG	GGSRASLLLCGDSSLSLTTCN
WP_100111498.1	Streptomyces xantholiticus	MALLDLQTMETPKSEATGELATGGSRASLLLCGDSSLSVTTCN	MALLDLQTMETPKSEATGELAT	GGSRASLLLCGDSSLSVTTCN
WP_030576627.1	Streptomyces sp. JS01	MALLDLQAMDTPAEDSFGELATGSQVSLLVCEFSSLSVVLCTP	MALLDLQAMDTPAEDSFGELA	TGSQVSLLVCEFSSLSVVLCTP
WP_052851312.1	Streptomyces avicenniae	MALLDLQAMDTPKDEALGDLATGSQVSLLICEFSSLSVALCTP	MALLDLQAMDTPKDEALGDLA	TGSQVSLLICEFSSLSVALCTP
WP_084749084.1	Kitasatospora albolonga	MALLDLQAMETQAEESFGELATGSQVSLLVCEYSSLSVVLCTP	MALLDLQAMETQAEESFGELA	TGSQVSLLVCEYSSLSVVLCTP
WP_030299708.1	Streptomyces katrae	MTLLDLQSMETPKEEVTEGALYTGGGGSRASLLLCGDSSLSITTCN	MTLLDLQSMETPKEEVTEGALYTGG	GGSRASLLLCGDSSLSITTCN
WP_133899219.1	Streptomyces sp. KS 21	MTLLDLQSMETPKEEVTEGALHGSGGGSRASLLLCGDSSLSITTCN	MTLLDLQSMETPKEEVTEGALHGSG	GGSRASLLLCGDSSLSITTCN
WP_019706186.1	Streptomyces sp. SID5475	MTLLDLQTMETPAEEITGELATGGGSRASLLLCGDSSLSVTTCN	MTLLDLQTMETPAEEITGELATG	GGSRASLLLCGDSSLSVTTCN
WP_053787310.1	Streptomyces sp. NRRL S-444	MTLLDLQSMETPKEEVTEGALHGGSGGSRASLLLCGDSSLSITTCN	MTLLDLQSMETPKEEVTEGALHGGS	GGSRASLLLCGDSSLSITTCN
WP_103501241.1	Streptomyces sp.	MALLDLQMMETPAAEEEAFGELATGSQVSLLICEHSSLSVTLCTP	MALLDLQMMETPAAEEEAFGELA	TGSQVSLLICEHSSLSVTLCTP
WP_190141253.1	Streptomyces libani subsp. rufus	MSLLDLQTLETPKAEATEELHTGSRASLLLCGDSSLSVSTCN	MSLLDLQTLETPKAEATEELH	TGSRASLLLCGDSSLSVSTCN
WP_202198001.1	Streptomyces spororaveus	MTLLDLQSMETPKEETTEAAHGGGSRASLLLCGDSSLSITTCN	MTLLDLQSMETPKEETTEAAHG	GGSRASLLLCGDSSLSITTCN
WP_016570339.1	Streptomyces albulus	MSLLDLQTLQTPEPEGNDDLHTGSRASLLLCGDSSLSVTTCK	MSLLDLQTLQTPEPEGNDDLH	TGSRASLLLCGDSSLSVTTCK
WP_048478819.1	Streptomyces sp. SID7760	MTLLDLQSMETPKEEATEVAMTGGGSRASLLLCGDSSLSVTTCN	MTLLDLQSMETPKEEATEVAMTG	GGSRASLLLCGDSSLSVTTCN
RNL73817.1	Streptomyces sp. I6	MSLLDLQTMETPKDELTDEIATGGSRASLLLCGDSSLSVTTCN	MSLLDLQTMETPKDELTDEIAT	GGSRASLLLCGDSSLSVTTCN
WP_167934310.1	Streptomyces sp. ventii	MALLDLQTMETPAVEEENFGELSKGSQVSLLICEHSSLSVTLCTP	MALLDLQTMETPAVEEENFGELS	KGSQVSLLICEHSSLSVTLCTP
WP_168100230.1	Streptomyces sp. PLAI1-29	MSLLDLQTMETPADENTGELGTTGSRASLLLCGDSSLSVTTCN	MSLLDLQTMETPADENTGELGT	TGSRASLLLCGDSSLSVTTCN
WP_100603006.1	Streptomyces sp. CB02959	MSLLDLQTLQTPEPETTDELHTGSRASLLLCGDSSLSVTTCN	MSLLDLQTLQTPEPETTDELH	TGSRASLLLCGDSSLSVTTCN
WP_109292635.1	Streptomyces spongiicola	MSLLDLQTMETPKDELTDEFGTGGSRASLLLCGDSSLSITTCN	MSLLDLQTMETPKDELTDEFGT	GGSRASLLLCGDSSLSITTCN
WP_093652864.1	Streptomyces wuyuanensis	MSLLDLQSMETPKDELTDEIATGGGSRASLLLCGDSSLSVTTCN	MSLLDLQSMETPKDELTDEIATG	GGSRASLLLCGDSSLSVTTCN
WP_125601303.1	Streptomyces sp. W1SF4	MTLLDLQSMETPKEETTEAAQLGGGGGSRASLLLCGDSSLSITTCN	MTLLDLQSMETPKEETTEAAQLGGG	GGSRASLLLCGDSSLSITTCN
WP_017946992.1	Streptomyces sp. PKU-MA01144	MSLLDLQSMETPKDELTDEIATGGSRASLLLCGDSSLSVTTCN	MSLLDLQSMETPKDELTDEIAT	GGSRASLLLCGDSSLSVTTCN
WP_053702377.1	Streptomyces sp. WM6368	MTLLDLQSMDTPKEETTEAAHGGGSRASLLLCGDSSLSITTCN	MTLLDLQSMDTPKEETTEAAHG	GGSRASLLLCGDSSLSITTCN
WP_053678097.1	Streptomyces sp. WM4235	MTLLDLQSMETPKEEITEAAMTGGGSRASLLLCGDSSLSITTCN	MTLLDLQSMETPKEEITEAAMTG	GGSRASLLLCGDSSLSITTCN
WP_030545200.1	Streptomyces albus	MTLLDLQTLETPAEEITGELATGGGSRASLLLCGDSSLSVTTCN	MTLLDLQTLETPAEEITGELATG	GGSRASLLLCGDSSLSVTTCN
WP_027749076.1	Streptomyces sp. CNH287	MTLLDLQTMETPKTEVSGELTGGGGSRASLLLCGDSSLSITTCN	MTLLDLQTMETPKTEVSGELTGG	GGSRASLLLCGDSSLSITTCN
WP_030708858.1	Streptomyces sp. NRRL F-2580	MTLLDLQSMETPKEETAEVAMTGGGSRASLLLCGDSSLSVTTCN	MTLLDLQSMETPKEETAEVAMTG	GGSRASLLLCGDSSLSVTTCN
WP_189040935.1	Verrucosispora sonchi	MALLDLQGMELPPTENYGGGSRASLLLCGDSSLSVTTCK	MALLDLQGMELPPTENYG	GGSRASLLLCGDSSLSVTTCK
WP_189040935.1	Micromonospora qiuiae	MALLDLQGMELPPTENYGGGSRASLLLCGDSSLSVTTCK	MALLDLQGMELPPTENYG	GGSRASLLLCGDSSLSVTTCK
WP_073918065.1	Streptomyces sp. CB00455	MTLLDLQSMDTPKEETTEAGHLTGGGSRASLLLCGDSSLSVTTCN	MTLLDLQSMDTPKEETTEAGHLTG	GGSRASLLLCGDSSLSVTTCN
WP_030962357.1	Streptomyces sp. NRRL S-378	MTLLDLQSMETPKEETAEVAMTGGGSRASLLLCGDSSLSITTCN	MTLLDLQSMETPKEETAEVAMTG	GGSRASLLLCGDSSLSITTCN
WP_046569477.1	Micromonospora sp. HK10	MALLDLQGMELPVAERHGGGSQASLLLCGDSSLSITTCN	MALLDLQGMELPVAERHG	GGSQASLLLCGDSSLSITTCN
WP_030655136.1	Streptomyces sp. NRRL S-104	MTLLDLQSMETPKEETTEVAMTGGGSRASLLLCGDSSLSITTCN	MTLLDLQSMETPKEETTEVAMTG	GGSRASLLLCGDSSLSITTCN
WP_088647629.1	Micromonospora aurantiaca	MALLDLQGMELPAAERTGGGSRASLLLCGDSSLSVTTCN	MALLDLQGMELPAAERTG	GGSRASLLLCGDSSLSVTTCN
WP_150520815.1	Streptomyces subrutilus	MTLLDLQSMETPKAEITEAGQLTGGGSRASLLLCGDSSLSITTCN	MTLLDLQSMETPKAEITEAGQLTG	GGSRASLLLCGDSSLSITTCN
WP_046569449.1	Micromonospora sp. HK10	MALLDLQGMELPAIERNGGGSQASLLLCGDSSLSITTCN	MALLDLQGMELPAIERNG	GGSQASLLLCGDSSLSITTCN
WP_069918637.1	Streptomyces subrutilus	MTLLDLQSMETPKEEATEVAMTGGGSRASLLLCGDSSLSLTTCN	MTLLDLQSMETPKEEATEVAMTG	GGSRASLLLCGDSSLSLTTCN
WP_202425639.1	Streptomyces sp. HUCO-GS316	METPKEEAMGDVETGSRASLLLCGDSSLSVTTCN	METPKEEAMGDVE	TGSRASLLLCGDSSLSVTTCN

WP_156205029.1	Streptomyces sp. TRM66187	MTLLDLQTLETPQEENAEMTGGGGSRASLLLCGDSSLSVTTCN	MTLLDLQTLETPQEENAEMTGG	GGSRASLLLCGDSSLSVTTCN
WP_107155630.1	Micromonospora sp. RP3T	MALLDLQGMELPATERTGGGSRASLLLCGDSSLSVTTCN	MALLDLQGMELPATERTG	GGSRASLLLCGDSSLSVTTCN
WP_185302290.1	Streptomyces finlayi	MALLDLQAMETSADESFGELATGSQVSLLVCEHSSLSLTLCTP	MALLDLQAMETSADESFGELA	TGSQVSLLVCEHSSLSLTLCTP
WP_121153817.1	Micromonospora pisi	MALLDLQGLELTAAELKGGDPGGSRASLLLCGDSSLSLVTCN	MALLDLQGLELTAAELKGGDP	GGSRASLLLCGDSSLSLVTCN
WP_199511144.1	Pseudosporangium sp. NEAU-24	MALLDLQGMELTTEQRNGGGGGSRASLLLCGDSSLSVTTCN	MALLDLQGMELTTEQRNGGG	GGSRASLLLCGDSSLSVTTCN
WP_030760152.1	Streptomyces sp. NRRL F-2664	MTLLDLQSMETPKEETSEVAMTGGGSRASLLLCGDSSLSITTCN	MTLLDLQSMETPKEETSEVAMTG	GGSRASLLLCGDSSLSITTCN
WP_098751688.1	Streptomyces sp. Ru87	MTLLDLQTLETPQEENTEMTGGGGSRASLLLCGDSSLSVTTCN	MTLLDLQTLETPQEENTEMTGG	GGSRASLLLCGDSSLSVTTCN
WP_189825575.1	Streptomyces spiroverticillatus	MSILDLQSMETPKEEATGDVELGGGGGGSRASLLLCGDSSLSVTTCH	MSILDLQSMETPKEEATGDVELGGGG	GGSRASLLLCGDSSLSVTTCH
WP_091258469.1	Micromonospora terminaliae	MALLDLQAMELPTTENHGGGSQASLLLCGDSSLSVTTCN	MALLDLQAMELPTTENHG	GGSQASLLLCGDSSLSVTTCN
WP_132266095.1	Micromonospora sp. KC721	MALLDLQGMELPAAERTGGGSRASLLLCGDSSLSVTTCK	MALLDLQGMELPAAERTG	GGSRASLLLCGDSSLSVTTCK
WP_100205048.1	Streptomyces carminius	MTLLDLQTMETPAEDEFTGGGSRASLLLCGDSSLSVTTCD	MTLLDLQTMETPAEDEFTG	GGSRASLLLCGDSSLSVTTCD
WP_150244045.1	Nocardiopsis quinghaiensis	MTLLDLQSLETAKNEAHGEVAGTSTASLLLCGDSSLSVTTC	MTLLDLQSLETAKNEAHGEVA	GTSTASLLLCGDSSLSVTTC
WP_138358110.1	Streptomyces sp. So13.3	MTLLDLQTLETPAAEDMGELATGGSRASLLLCGNSSLSVTTCN	MTLLDLQTLETPAAEDMGELAT	GGSRASLLLCGNSSLSVTTCN
WP_109816733.1	Micromonospora sp. 5R2A7	MALLDLQGMELPAAERTGGGSQASLLLCGDSSLSITTCN	MALLDLQGMELPAAERTG	GGSQASLLLCGDSSLSITTCN
WP_107079502.1	Micromonospora sp. MH33	MALLDLQAMELPTTENHGGGSQASLLLCGDSSLSITTCN	MALLDLQAMELPTTENHG	GGSQASLLLCGDSSLSITTCN
WP_130877530.1	Streptomyces netropsis	MTLLDLQTMETPKAEVTNEFGGGGGGSRASLLLCGDSSLSVVTCN	MTLLDLQTMETPKAEVTNEFGGGG	GGSRASLLLCGDSSLSVVTCN
WP_067312693.1	Micromonospora rifamycinica	MALLDLQAMELPATERTGGGSQASLLLCGDSSLSVTTCN	MALLDLQAMELPATERTG	GGSQASLLLCGDSSLSVTTCN
WP_091302793.1	Micromonospora sp. AMSO31t	MALLDLQGMELPAAERTGGGSQASLLLCGDSSLSVTTCH	MALLDLQGMELPAAERTG	GGSQASLLLCGDSSLSVTTCH
WP_204037917.1	Micromonospora qiuiae	MALLDLQGMELPTTENYGGGSQASLLLCGNSSLSVTTCN	MALLDLQGMELPTTENYG	GGSQASLLLCGNSSLSVTTCN
WP_031508406.1	Streptomyces megasporus	MTLLDLQTMETPQDDEFTGGGSQASLLLCGDSSLSLVTCN	MTLLDLQTMETPQDDEFTG	GGSQASLLLCGDSSLSLVTCN
WP_132237491.1	Micromonospora sp. AMSO12t	MALLDLQGMEMAPADRTGGGSRASLLLCGDSSLSVTTCN	MALLDLQGMEMAPADRTG	GGSRASLLLCGDSSLSVTTCN
WP_184364099.1	Nocardiopsis metallicus	MTLLDLQSMETAKNEAHGGSGGTSTASLLLCGDSSLSVTTC	MTLLDLQSMETAKNEAHGGSG	GTSTASLLLCGDSSLSVTTC
WP_088961893.1	Micromonospora purpureochromogenes	MALLDLQGMELPAAERTGGGSQASLLLCGDSSLSITTCH	MALLDLQGMELPAAERTG	GGSQASLLLCGDSSLSITTCH
WP_088974650.1	Micromonospora coxensis	MALLDLQGMELPAAERTGGGSQASLLLCGDSSLSVTTCR	MALLDLQGMELPAAERTG	GGSQASLLLCGDSSLSVTTCR
WP_189040934.1	Verrucosispora sonchi	MALLDLQGMELPTTENYGGGSQASLLLCGDSSLSVTTCK	MALLDLQGMELPTTENYG	GGSQASLLLCGDSSLSVTTCK
WP_040271139.1	Streptomonospora alba	MTLLDLQSLETVNEARGEIAHGSEASLLLCGDSSLSVTTCN	MTLLDLQSLETVNEARGEIA	HGSEASLLLCGDSSLSVTTCN
WP_088974649.1	Micromonospora aurantiaca	MALLDLQGMELPAAERTGGGSQASLLLCGDSSLSVTTCK	MALLDLQGMELPAAERTG	GGSQASLLLCGDSSLSVTTCK
WP_067312695.1	Micromonospora rifamycinica	MALLDLQGMELPAAGRTGGGSQASLLLCGDSSLSVTTCN	MALLDLQGMELPAAGRTG	GGSQASLLLCGDSSLSVTTCN
WP_109816734.1	Micromonospora sp. 5R2A7	MALLDLQGMELPTAERTGGGSQASLLLCGDSSLSITTCN	MALLDLQGMELPTAERTG	GGSQASLLLCGDSSLSITTCN
OLT29379.1	Nocardiopsis sp. CNR-923	MTLLDLQSLETVQNEAAGEVAGGSVASLLLCGDSSLSLTTC	MTLLDLQSLETVQNEAAGEVA	GGSVASLLLCGDSSLSLTTC
WP_116246873.1	Nocardiopsis sp. FIRDI 009	MTLLDLQSLETVKDEVHGEVAGTSTASLTLCGDSSLSVTTC	MTLLDLQSLETVKDEVHGEVA	GTSTASLTLCGDSSLSVTTC
WP_088961894.1	Micromonospora terminaliae	MALLDLQGMELPAAERTGGGSQASLLLCGDSSLSITTCK	MALLDLQGMELPAAERTG	GGSQASLLLCGDSSLSITTCK
WP_017580486.1	Nocardiopsis valliformis	MTLLDLQSLETAKNEAHGEAGGTSTASLLLCGDSSLSITTC	MTLLDLQSLETAKNEAHGEAG	GTSTASLLLCGDSSLSITTC
WP_031143925.1	Streptomyces xanthophaeus	MTLLDLQSMDTVKEETTEAAHLTGGGSRASLLLCGDSSLSITTCN	MTLLDLQSMDTVKEETTEAAHLTG	GGSRASLLLCGDSSLSITTCN
WP_114657750.1	Streptomyces paludis	MNLLDLQSMEIPQEEAIGETATASYASLLLCFGSALSLLTC	MNLLDLQSMEIPQEEAIGETA	TASYASLLLCFGSALSLLTC
WP_159942321.1	Nocardiopsis sp. FR4	MSLLDLQSLETVKDEAHGEAASPSAASLLLCGDSSLSLTTCR	MSLLDLQSLETVKDEAHGEAA	SPSAASLLLCGDSSLSLTTCR
WP_164446950.1	Verrucosispora sonchi	MALLDLQGMEAAPADRTGGGSRASLLLCGDSSLSVTTCN	MALLDLQGMEAAPADRTG	GGSRASLLLCGDSSLSVTTCN
WP_089003486.1	Micromonospora sp. M71_S20	MALLDLQGMEMAPADRTGGGSRASLLLCGDSSLSVTTCN	MALLDLQGMEMAPADRTG	GGSRASLLLCGDSSLSVTTCN
WP_125807435.1	Actinoplanes sp. ATCC 53533	MALLDLQGMELSAEQRGGGDGSRASLLLCGDSSLSVTTCN	MALLDLQGMELSAEQRGGG	DGSRASLLLCGDSSLSVTTCN
WP_150205781.1	Streptomyces venezuelae	MTLLDLQSMETIKEETTEAGLTGGGGSRASLLLCGDSSLSITTCN	MTLLDLQSMETIKEETTEAGLTGG	GGSRASLLLCGDSSLSITTCN
WP_028649330.1	Nocardiopsis sp. CNT312	MTLFDLQSLETAKDEAHGEATATSTASLLLCGDSSLSVTTC	MTLFDLQSLETAKDEAHGEAT	ATSTASLLLCGDSSLSVTTC
WP_030162116.1	Streptomyces sp. WM6372	MTLLDLQSMETPKEEVTEGALHGGGGGGSRASLLLCGDSSLSITTCN	MTLLDLQSMETPKEEVTEGALHGGGG	GGSRASLLLCGDSSLSITTCN

WP_091302791.1	Micromonospora halophytica	MALLDLQGMQLPAAERTGGGSQASLLLCGDSSLSVTTCK	MALLDLQGMQLPAAERTG	GGSQASLLLCGDSSLSVTTCK
WP_191268118.1	Nocardiopsis terrae	MTLLDLQSLETATNEAHGEVAGTSTASLLLCGDSSLSVTTC	MTLLDLQSLETATNEAHGEVA	GTSTASLLLCGDSSLSVTTC
WP_091306273.1	Micromonospora chersina	MALLDLQAMELPTTENYGGGSQASLLLCGDSSLSLTTCH	MALLDLQAMELPTTENYG	GGSQASLLLCGDSSLSLTTCH
WP_199511143.1	Pseudosporangium sp. NEAU-24	MALMDLQGMELSAERNGGGGSRASLLLCGDSSLSVTTCN	MALMDLQGMELSAERNGG	GGSRASLLLCGDSSLSVTTCN
WP_071807433.1	Couchioplanes caeruleus	MALLDLQGMELTSEAAYGGGSRASLLLCGDSSLSVTTCN	MALLDLQGMELTSEAAYG	GGSRASLLLCGDSSLSVTTCN
WP_091089080.1	Micromonospora nigra	MALLDLQAMEAAPADRTGGGSQASLLLCGDSSLSVTTCN	MALLDLQAMEAAPADRTG	GGSQASLLLCGDSSLSVTTCN
WP_107155631.1	Micromonospora sp. RP3T	MALLDLQAMELPATERTGGGSGASLLLCGDSSLSVTTCN	MALLDLQAMELPATERTG	GGSGASLLLCGDSSLSVTTCN
WP_091439122.1	Micromonospora yangpuensis	MALLDLQGMEMAPADRTGDGSKASLLLCGDSSLSVTTCN	MALLDLQGMEMAPADRTG	DGSKASLLLCGDSSLSVTTCN
WP_091258467.1	Micromonospora sp. AP08	MALLDLQGMELPAAERTGGGSQASLLLCGDSSLSLTTCK	MALLDLQGMELPAAERTG	GGSQASLLLCGDSSLSLTTCK
PZM96828.1	Actinobacteria bacterium	MALLDLQGMELPSEGYAEYVGGSRASLLLCGNSSLSLVTCL	MALLDLQGMELPSEGYAEYV	GGSRASLLLCGNSSLSLVTCL
WP_017597754.1	Nocardiopsis lucentensis	MTLLDLQSLETVKDEVHGEVAGTSTASLTLCGDSSLSLTTC	MTLLDLQSLETVKDEVHGEVA	GTSTASLTLCGDSSLSLTTC
WP_189200955.1	Couchioplanes caeruleus	MALMDLQGMELTPEAAYGGGSRASLLLCGDSSLSVTTCD	MALMDLQGMELTPEAAYG	GGSRASLLLCGDSSLSVTTCD
WP_091439120.1	Micromonospora yangpuensis	MALLDLQGLEMAPADRTGDGSRASLLLCGDSSLSVTTCN	MALLDLQGLEMAPADRTG	DGSRASLLLCGDSSLSVTTCN
WP_093405935.1	Verrucosispora sp. CNZ293	MALLDLQGLEAAPADRTGGGSRASLLLCGDSSLSVTTCN	MALLDLQGLEAAPADRTG	GGSRASLLLCGDSSLSVTTCN
WP_093405935.1	Micromonospora andamanensis	MALLDLQGLEAAPADRTGGGSRASLLLCGDSSLSVTTCN	MALLDLQGLEAAPADRTG	GGSRASLLLCGDSSLSVTTCN
WP_182853782.1	Streptomyces durbertensis	MSLLDLQTMETPAVEESLDELATGSQVSLLVCEHSSLSVTLCTP	MSLLDLQTMETPAVEESLDELA	TGSQVSLLVCEHSSLSVTLCTP
WP_125817414.1	Streptomyces sp. WAC07149	MTLLDLQSMETPKEENTEAAQLGGGGGGSRASLLLCGDSSLSITTCN	MTLLDLQSMETPKEENTEAAQLGGGG	GGSRASLLLCGDSSLSITTCN
WP_203780788.1	Actinoplanes rishiriensis	MTLLDLQGMQVSATERGGGGGSQASLLLCGDSSLSVTTCN	MTLLDLQGMQVSATERGGG	GGSQASLLLCGDSSLSVTTCN
WP_158241974.1	Micromonospora sp. CNZ309	MALLDLQGMEMAPADRTGGGSNASLLLCGDSSLSVTTCN	MALLDLQGMEMAPADRTG	GGSNASLLLCGDSSLSVTTCN
WP_013734295.1	Micromonospora sp. ATA51	MALLDLQGLEMAPADRTGGGSRASLLLCGDSSLSVTTCN	MALLDLQGLEMAPADRTG	GGSRASLLLCGDSSLSVTTCN
WP_073786186.1	Streptomyces sp. SID4919	MALLDLQALDTPTEEFGDTATGSQVSLIVCEYSSLSALLCTP	MALLDLQALDTPTEEFGDTA	TGSQVSLIVCEYSSLSALLCTP
WP_136215832.1	Streptomyces cirratus	MTLLDLQSMETVKEETTEAALHGGGSQASLLLCGDSSLSLTTCH	MTLLDLQSMETVKEETTEAALHG	GGSQASLLLCGDSSLSLTTCH
WP_017621962.1	Nocardiopsis chromatogenes	MSLLDLQTLEPAADEALTGGGGGSEASLLLCGDSSLSLTTCH	MSLLDLQTLEPAADEALTGGG	GGSEASLLLCGDSSLSLTTCH
WP_120569348.1	Micromonospora tulbaghiae	MALLDLQGLEMAPADRTGGGSRASLLLCGDSSLSITTCN	MALLDLQGLEMAPADRTG	GGSRASLLLCGDSSLSITTCN
WP_112449185.1	Streptomyces sp. ICC4	MTLLDLQSMETPKEETTELHGGGGGGSRASLLLCGDSSLSITTCN	MTLLDLQSMETPKEETTELHGGGG	GGSRASLLLCGDSSLSITTCN
WP_030867508.1	Streptomyces sp. NRRL F-2747	MTLLDLQSMETPKEEVTEGALLGGGGGGSRASLLLCGDSSLSITTCN	MTLLDLQSMETPKEEVTEGALLGGGG	GGSRASLLLCGDSSLSITTCN
WP_132396327.1	Micromonospora sp. KC207	MALLDLQGLEMAPADRNGGGSNASLLLCGDSGLSVTTCN	MALLDLQGLEMAPADRNG	GGSNASLLLCGDSGLSVTTCN
WP_033340571.1	Catenuloplanes japonicus	MTLLDLQGMHIEDTDRNGGGGGGSQASLLLCGDSSLSVTTCN	MTLLDLQGMHIEDTDRNGGGG	GGSQASLLLCGDSSLSVTTCN
WP_189112533.1	Pilimelia terevasa	MALLDLQAMDLSGYGDGGYNSRASLLLCGDSSLSLTTCN	MALLDLQAMDLSGYGDGG	YNSRASLLLCGDSSLSLTTCN
WP_009997859.1	Streptomyces clavuligerus	MALLDLQTLEIPAEENLGELETGSSQSLLVCGPHSSQSLLLCN	MALLDLQTLEIPAEENLGELE	TGSSQSLLVCGPHSSQSLLLCN
WP_016700261.1	Actinoalloteichus spitiensis	MESVLDLQAMEASEDAAEFASPGASQASLLLCGDSSLSVVTCR	MESVLDLQAMEASEDAAEFASP	GASQASLLLCGDSSLSVVTCR
WP_184730207.1	Streptomyces netropsis	MTLLDLQTMETPKTEVTGELGHGGGGGSRASLLLCGDSSLSVVTCN	MTLLDLQTMETPKTEVTGELGHGGG	GGSRASLLLCGDSSLSVVTCN
QDY75293.1	Streptomyces qinzhouensis	MALLDLQALEITEDEAFGDVETGSSLSLLSCGHHSNLSLLAC	MALLDLQALEITEDEAFGDVE	TGSSLSLLSCGHHSNLSLLAC
WP_026418839.1	Actinoalloteichus cyanogriseus	MESVLDLQAMEATEDAAEFASPGGSQASLLLCGDSGLSVLTCR	MESVLDLQAMEATEDAAEFASP	GGSQASLLLCGDSGLSVLTCR
WP_020551011.1	Streptomyces sp. SID5474	MALLDLQALETDEAPEFGGGGGGSRASLLLCGNSSLSLVTCN	MALLDLQALETDEAPEFGGGG	GGSRASLLLCGNSSLSLVTCN
WP_145776375.1	Micromonospora olivasterospora	MALLDLQGLEMAPADRTGGGSNASLLLCGDSSLSVTTCR	MALLDLQGLEMAPADRTG	GGSNASLLLCGDSSLSVTTCR
WP_203776271.1	Actinoplanes nipponensis	MALLDLQGMELSTEAKNSGGGGGSRASLLLCGDSSLSVTTCN	MALLDLQGMELSTEAKNSGGG	GGSRASLLLCGDSSLSVTTCN
WP_165300811.1	Streptomyces boncukensis	MALLDLQTMEPEETATHGGGGSVASLLLCGDSALSLVTCHH	MALLDLQTMEPEETATHGG	GGSVASLLLCGDSALSLVTCHH
WP_101366223.1	Plantactinospora sp. BC1	MALLDLQGMELADRTGGGGGGGGSRASLLLCGDSSLSVTTCN	MALLDLQGMELADRTGGGGGG	GGSRASLLLCGDSSLSVTTCN
WP_161362793.1	Streptomyces sp. SID3343	MTLLDLQTMATPAIEDGGELANGGGGGGGSRASLLLCGNSSLSLVTCN	MTLLDLQTMATPAIEDGGELANGGGGG	GGSRASLLLCGNSSLSLVTCN
WP_189059358.1	Longimycelium tulufanense	MNLVLDLQAMETGEELGHGGGYDAGSNASLLLCGDSSLSVTTCD	MNLVLDLQAMETGEELGHGGGYD	AGSNASLLLCGDSSLSVTTCD
WP_206346249.1	Streptomyces verrucosisporus	MTLLDLQTLETSEDKGTVAGGGSEASLLLCGDSSLSVTTCN	MTLLDLQTLETSEDKGTVAG	GGSEASLLLCGDSSLSVTTCN
WP_117669281.1	Micromonospora sp. NEAU-HG-1	MALLDLQGLEMAPADRTGGGSSASLLLCGDSSLSITTCN	MALLDLQGLEMAPADRTG	GGSSASLLLCGDSSLSITTCN

WP_043963346.1	Micromonospora haikouensis	MALLDLQGLEMAPADRTGGGSSASLLLCGDSGLSVTTCN	MALLDLQGLEMAPADRTG	GGSSASLLLCGDSGLSVTTCN
WP_131099445.1	Streptomonospora sp. M2	MSLLDLQSLETTTEAHGEVSAGSEASLLLCGDSSLSLTTCN	MSLLDLQSLETTTEAHGEVS	AGSEASLLLCGDSSLSLTTCN
WP_093654844.1	Streptomyces radiopugnans	MTLLDLQTLETSEDKGTVAGGGSEASLLLCGDSSLSVTTCR	MTLLDLQTLETSEDKGTVAG	GGSEASLLLCGDSSLSVTTCR
WP_199443385.1	Umezawaea sp. REN6	MENILDLQVLDTPEGLELTGGGSRASLLLCGDSSLSLTTCN	MENILDLQVLDTPEGLELTG	GGSRASLLLCGDSSLSLTTCN
WP_074476206.1	Micromonospora carbonacea	MTLLDLQGLELAPADRTGGGSSASLLLCGDSGLSVTTCN	MTLLDLQGLELAPADRTG	GGSSASLLLCGDSGLSVTTCN
WP_203865853.1	Plantactinospora endophytica	MALLDLQGMELADRTGGGGGGGGSRASLLLCGDSSLSVTTCD	MALLDLQGMELADRTGGGGGG	GGSRASLLLCGDSSLSVTTCD
WP_043963347.1	Micromonospora haikouensis	MALLDLQGLEMAPADRTSGDSRASLLLCGDSGLSVTTCN	MALLDLQGLEMAPADRTS	GDSRASLLLCGDSGLSVTTCN
WP_186767899.1	Streptomyces qinzhouensis	MALLDLQALEITEDEAFGDVETGSSLSLLSCGHHSNLSLLAC	MALLDLQALEITEDEAFGDVE	TGSSLSLLSCGHHSNLSLLAC
WP_040912782.1	Streptomyces sp. SID5473	MALLDLQALEITEDEAFGDVETGSNLSLTSCGHNSHLSLLAC	MALLDLQALEITEDEAFGDVE	TGSNLSLTSCGHNSHLSLLAC
WP_074476205.1	Micromonospora carbonacea	MALLDLQGLELAPADRTSGDSRASLLLCGDSGLSVTTCN	MALLDLQGLELAPADRTS	GDSRASLLLCGDSGLSVTTCN
WP_130463203.1	Plantactinospora sp. CNZ321	MALLDLQGMELADRTGGGDGGGSRASLLLCGDSSLSVTTCN	MALLDLQGMELADRTGGGDG	GGSRASLLLCGDSSLSVTTCN
WP_192769447.1	Plantactinospora soyae	MALLDLQGMELADRTGGGGGGGSRASLLLCGDSSLSVTTCN	MALLDLQGMELADRTGGGGG	GGSRASLLLCGDSSLSVTTCN
WP_123560779.1	Micromonospora sp. HM5-17	MALLDLQGMELTPADRGGGDGGCGCGGSRASLLLCGDSSLSVTTCN	MALLDLQGMELTPADRGGGDGGCGC	GGSRASLLLCGDSSLSVTTCN
WP_075740515.1	Actinoalloteichus fjordicus	MEFVLDLQATETPDAHLEVAGGGAGGSRASLLLCGDSGLSVVTCN	MEFVLDLQATETPDAHLEVAGGGA	GGSRASLLLCGDSGLSVVTCN
WP_015621708.1	Actinoplanes sp. N902-109	MTLLDLQGMSVSTKNGGHGGGGGSQASLLLCGDSSLSVTTCN	MTLLDLQGMSVSTKNGGHGGG	GGSQASLLLCGDSSLSVTTCN
WP_109594144.1	Actinoplanes xinjiangensis	MTLLDLQGMHVSASERTGGGGGGGSQASLLLCGDSSLSVTTCN	MTLLDLQGMHVSASERTGGGGG	GGSQASLLLCGDSSLSVTTCN
WP_132396325.1	Micromonospora sp. KC207	MALLDLQGLEMAPADRTSGSSASLLLCGDSGLSVTTCN	MALLDLQGLEMAPADRT	SGSSASLLLCGDSGLSVTTCN
WP_182666608.1	Streptomyces calidiresistens	MALLDLQMMDAAPETGETGLQSTASLLLCGDSSLSVVTC	MALLDLQMMDAAPETGETG	LQSTASLLLCGDSSLSVVTC
WP_143622836.1	Streptomyces alkaliphilus	MALLDLQMMDATPETTEFGDTSTASLLLCGDSSLSVVTC	MALLDLQMMDATPETTEFG	DTSTASLLLCGDSSLSVVTC
WP_091556107.1	Micromonospora pattaloongensis	MALLDLQGMELKGGGNGGGSRASLLLCGDSSLSVTTCN	MALLDLQGMELKGGGNG	GGSRASLLLCGDSSLSVTTCN
WP_189171742.1	Pilimelia anulata	MALLDLQAMELSGYGDGDGHGGWGHGGFNSRASLLLCGDSSLSVTTCN	MALLDLQAMELSGYGDGDGHGGWGHGG	FNSRASLLLCGDSSLSVTTCN
WP_153524591.1	Streptomyces jumonjinensis	MALLDLQALETPAEEAMDVETNSNLSLLLCQGSSLSLTC	MALLDLQALETPAEEAMDVE	TNSNLSLLLCQGSSLSLTC
WP_153481488.1	Streptomyces katsurahamanus	MALLDLQALETPAEEAMDVETVSNLSLLLCQGSSLSLTC	MALLDLQALETPAEEAMDVE	TVSNLSLLLCQGSSLSLTC
TML25266.1	Actinobacteria bacterium	MALLDLQAMELAADATRGGGRGSNLSVTGCGGKSSLSVIDCF	MALLDLQAMELAADATRGGG	RGSNLSVTGCGGKSSLSVIDCF
WP_160504790.1	Streptomyces sp. BA2	MALLDLQSLEADEMTGGGGGGGVSYASLLLCGDSGLSVITC	MALLDLQSLEADEMTGGGGGG	GVSYASLLLCGDSGLSVITC
WP_156726775.1	Streptomyces sp. TRM66233	MALLDLQNMETELTGGGDVSYASLLLCGDSGLSVLTC	MALLDLQNMETELTGGG	DVSYASLLLCGDSGLSVLTC
WP_103844057.1	Streptomyces sp. Ru72	MSLLDLQTMETPEPQEDELHGGGSEISLTLCNSAASVTLCL	MSLLDLQTMETPEPQEDELHG	GGSEISLTLCNSAASVTLCL
MBN3872516.1	Nostoc sp. JL33	MINIKNHDITVAGTDFFADSESFLNELTDSETVQLNEANGGIASADSGASLLLCGDS ALSIVTCN	MINIKNHDITVAGTDFFADSESFLNELTDSETVQL NEANGGIAS	ADSGASLLLCGDSALSIVTCN
EDX20848.1	Streptomyces sp. Mg1	MAILDLQTLELPETEAHPIDDTLASTSSLSVLNCGTSTVSTLVCL	MAILDLQTLELPETEAHPIDDTLA	STSSLSVLNCGTSTVSTLVCL
WP_088983868.1	Micromonospora echinospora	MTLLDLQGMEADRTHGGGDTSGLSLLLCEGSSLSVTSCD	MTLLDLQGMEADRTHGGG	DTSGLSLLLCEGSSLSVTSCD
WP_131896067.1	Jiangella asiatica	MALLDLQGMEAPGRKGHGGGSTLTVLTCGSVKPSNLSVALCH	MALLDLQGMEAPGRKGHG	GGSTLTVLTCGSVKPSNLSVALCH
WP_157247321.1	Nonomuraea typhae	MTHVLDLQRLTNPDADAERAVAGNSSISFTLCGDSSTLSVLLCHGCHCPH	MTHVLDLQRLTNPDADAERAVA	GNSSISFTLCGDSSTLSVLLCHGCHC PH
WP_091461528.1	Micromonospora inyonensis	MTLLDLQGMEADRTGGHGDTSGLSLLMCDGSSLSVTSCD	MTLLDLQGMEADRTGGHG	DTSGLSLLMCDGSSLSVTSCD
WP_197002359.1	Longispora fulva	MTLLDLQGLTTAPEHGHGGSNLSVALCGESGLSVLLCD	MTLLDLQGLTTAPEHGH	GGSNLSVALCGESGLSVLLCD
WP_017539204.1	Nocardiopsis halophila	MSLLDLQTLEPAKDEAAGGGGGSEASLLLCGDSSLSLTTCN	MSLLDLQTLEPAKDEAAGGG	GGSEASLLLCGDSSLSLTTCN

NMR spectroscopy data

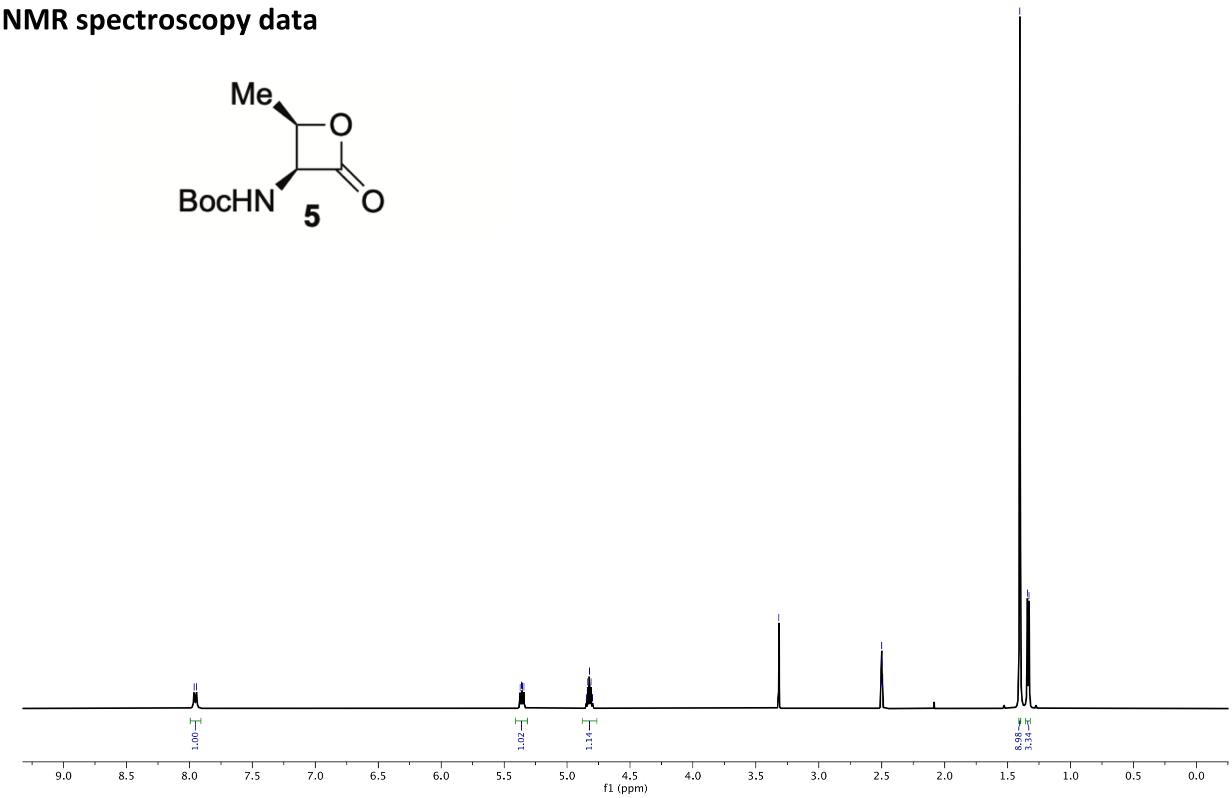

Figure S18. ^{1}H NMR spectrum of **5** in DMSO-d_6.

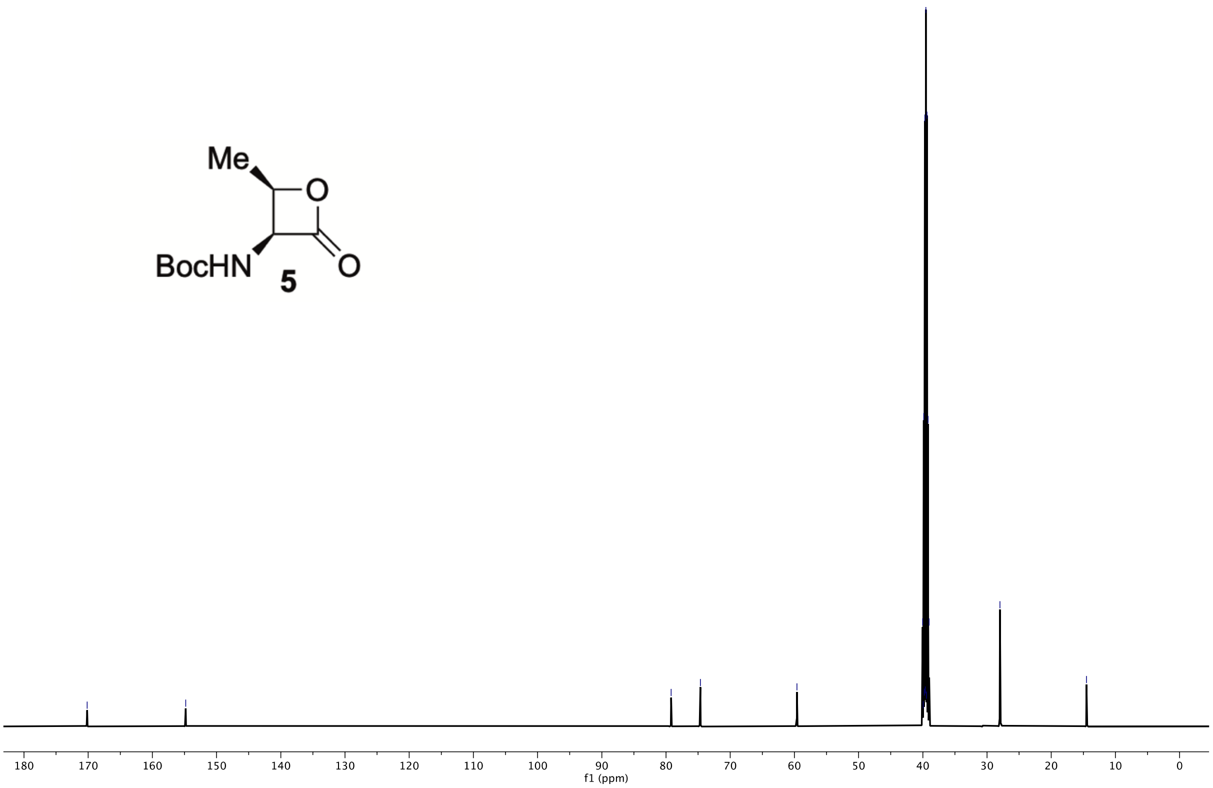

Figure S19. ^{13}C NMR spectrum of **5** in DMSO-d$_6$.

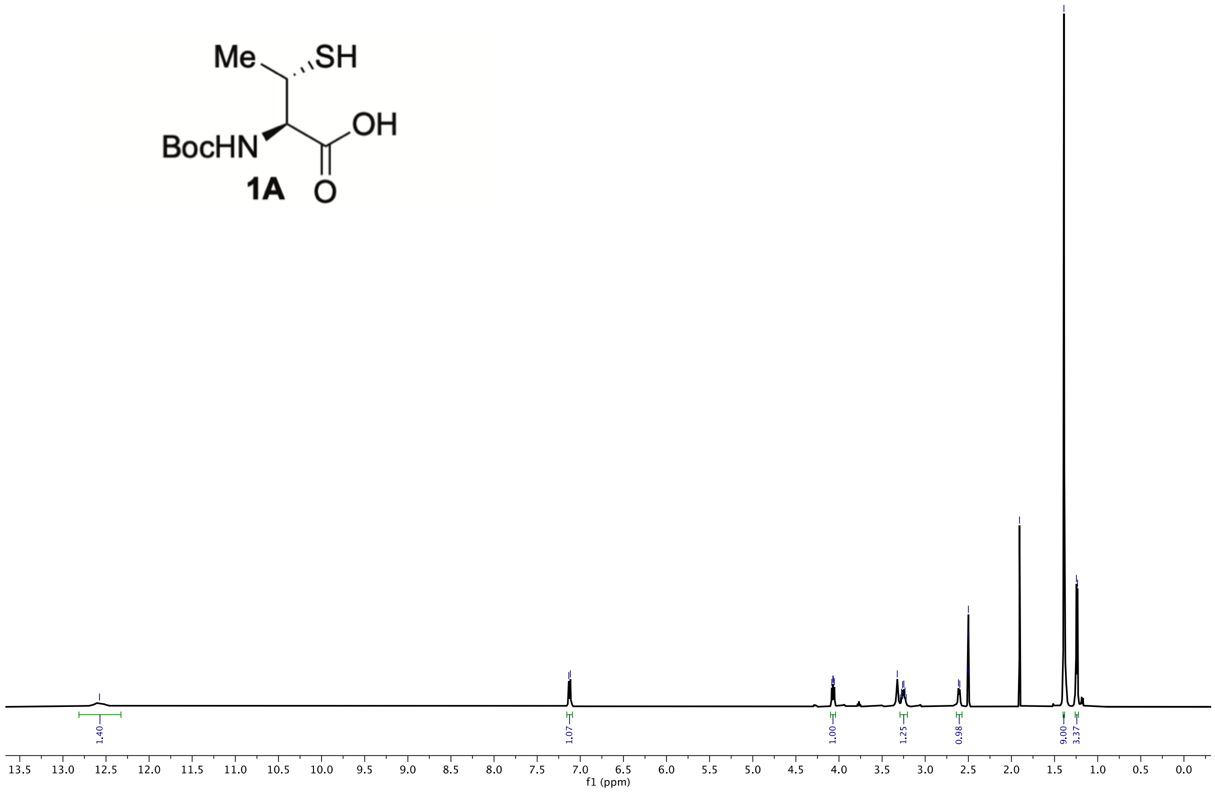

Figure S20. ^{1}H NMR spectrum of **1A** in DMSO-d_6.

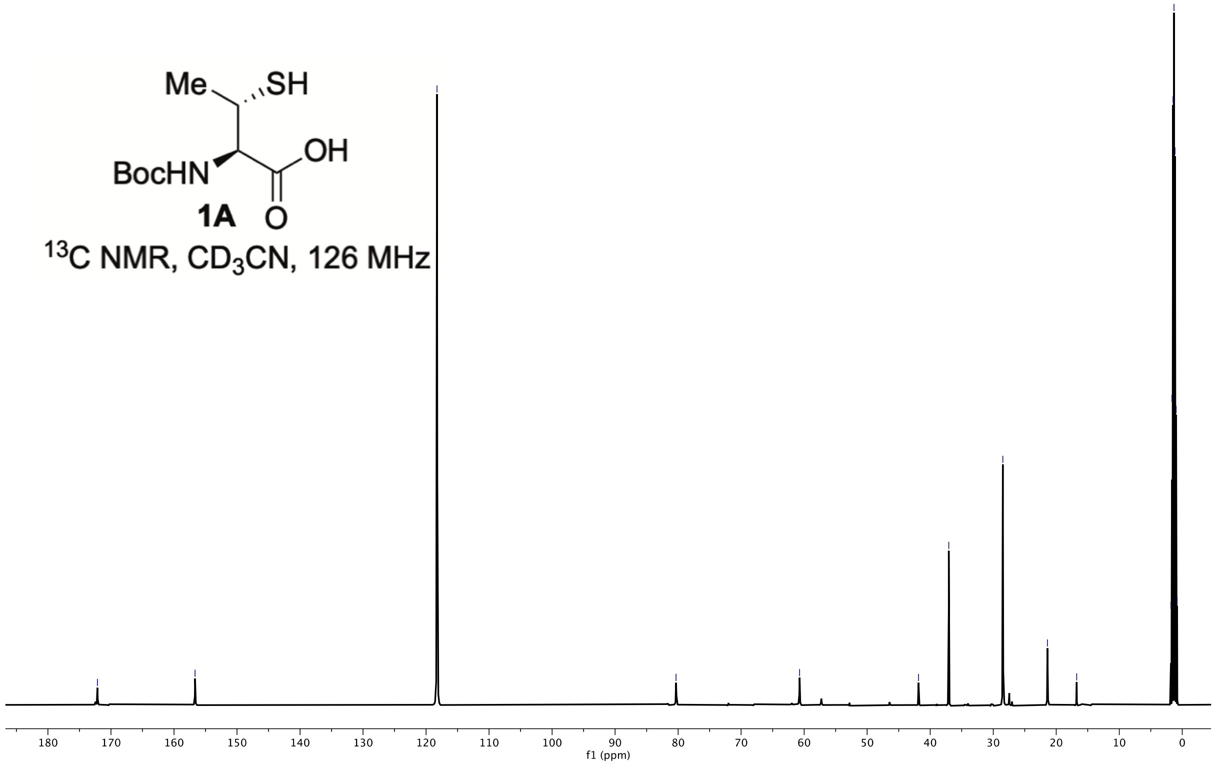

Figure S21. ^{13}C NMR spectrum of **1A** in CD_3CN.

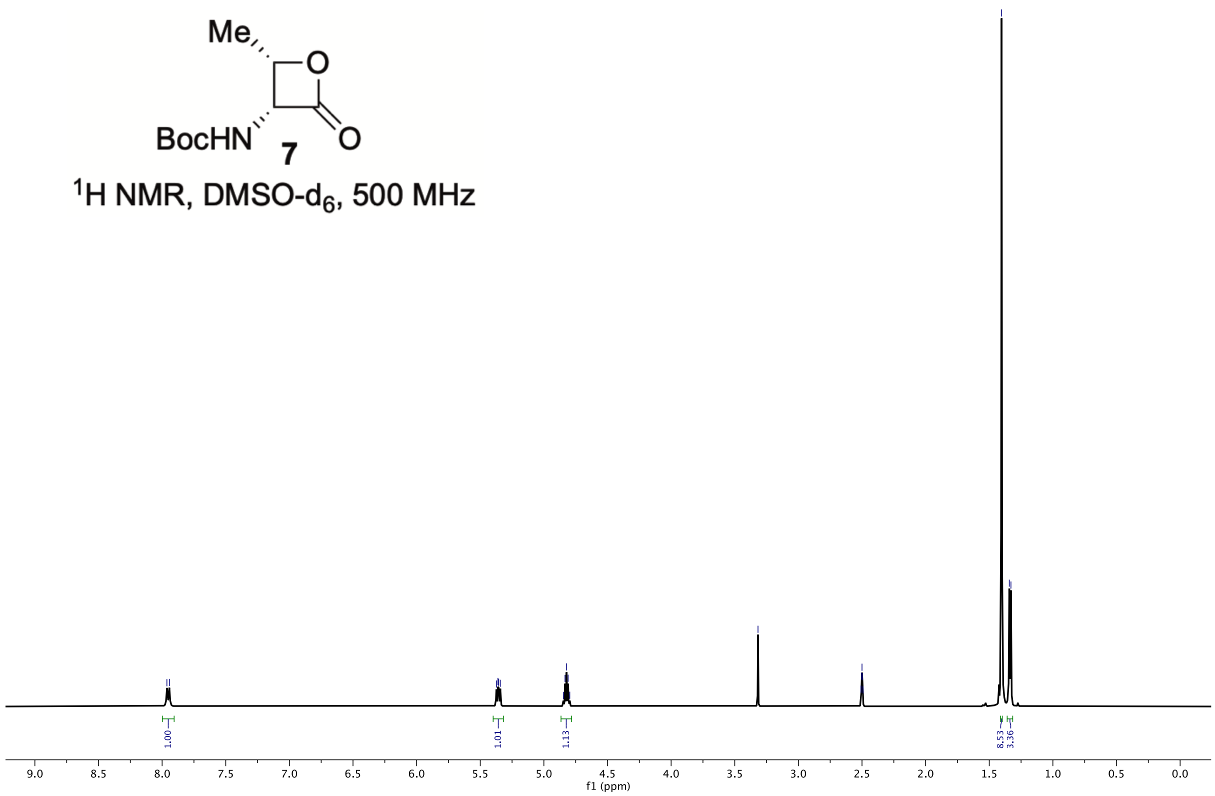

Figure S22. ^{1}H NMR spectrum of **7** in DMSO-d$_6$.

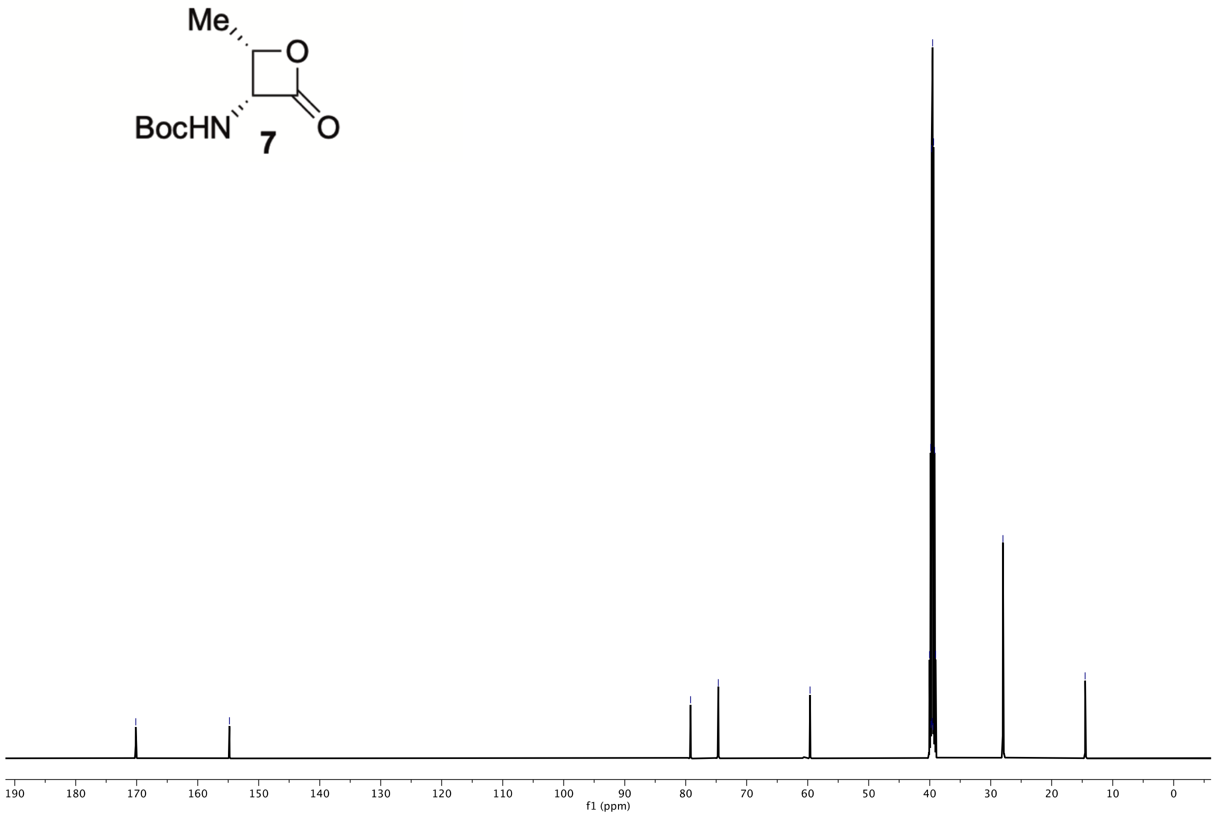

Figure S23. ^{13}C NMR spectrum of **7** in DMSO-d_6.

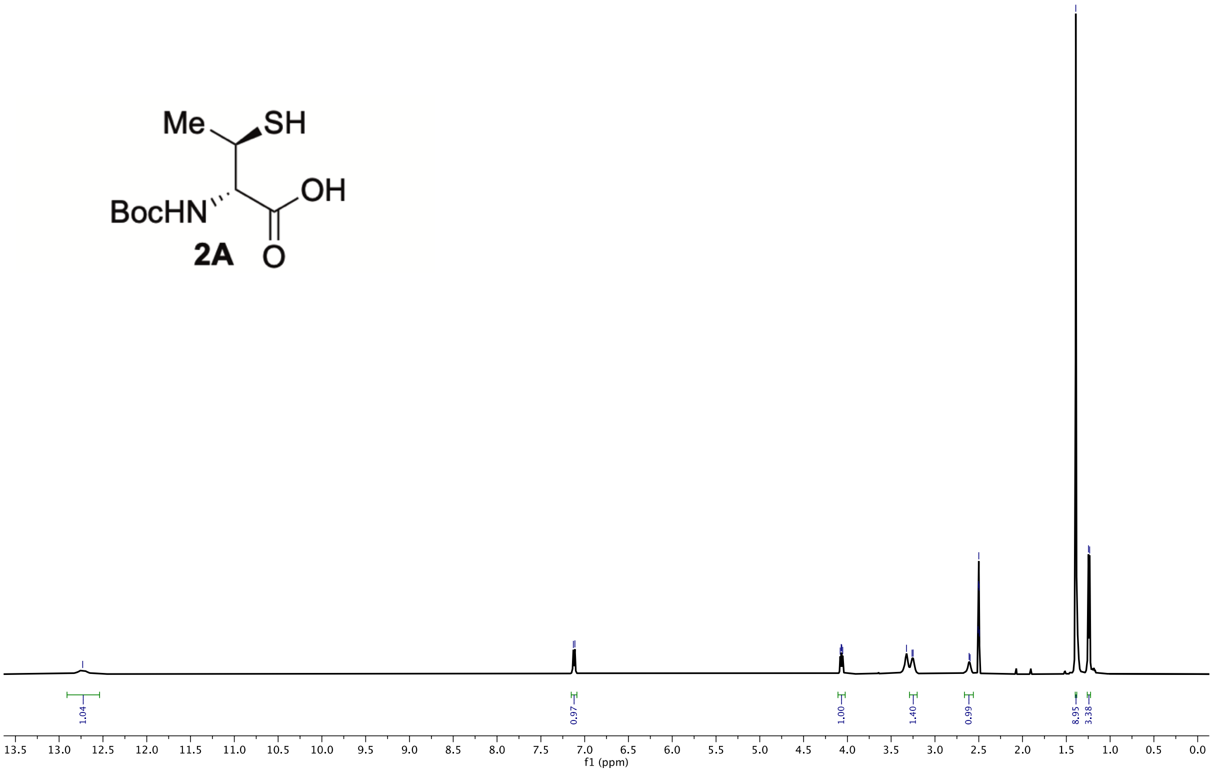

Figure S24. ^{1}H NMR spectrum of **2A** in DMSO-d_6.

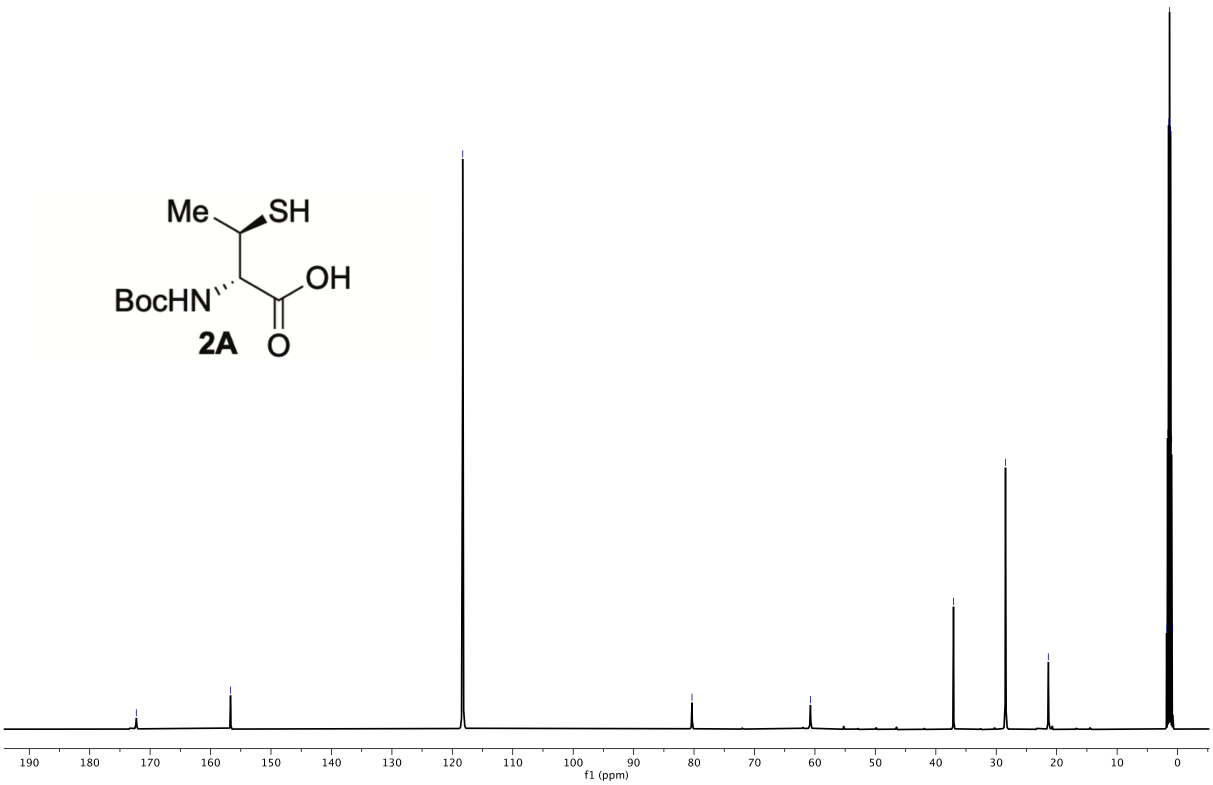

Figure S25. ^{13}C NMR spectrum of **2A** in CD_3CN.

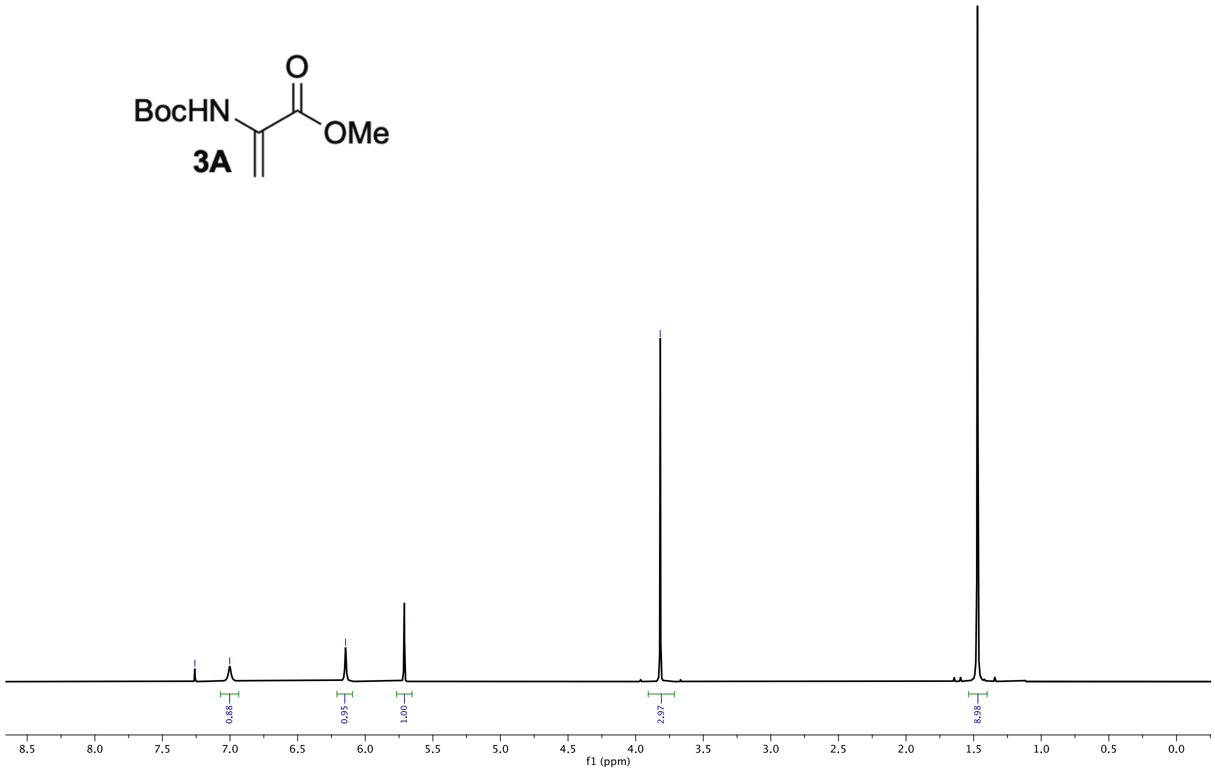

Figure S26. ^{1}H NMR spectrum of **3A** in $CDCl_3$.

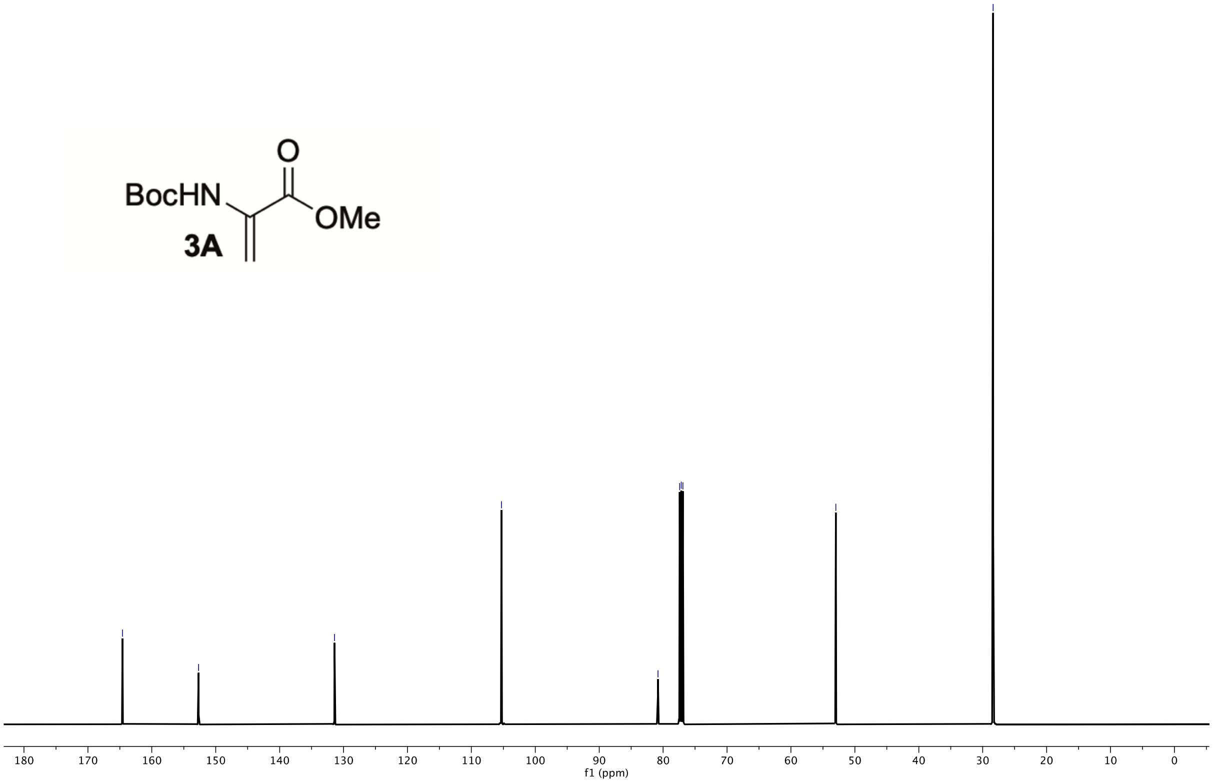

Figure S27. ^{13}C NMR spectrum of **3A** in $CDCl_3$.

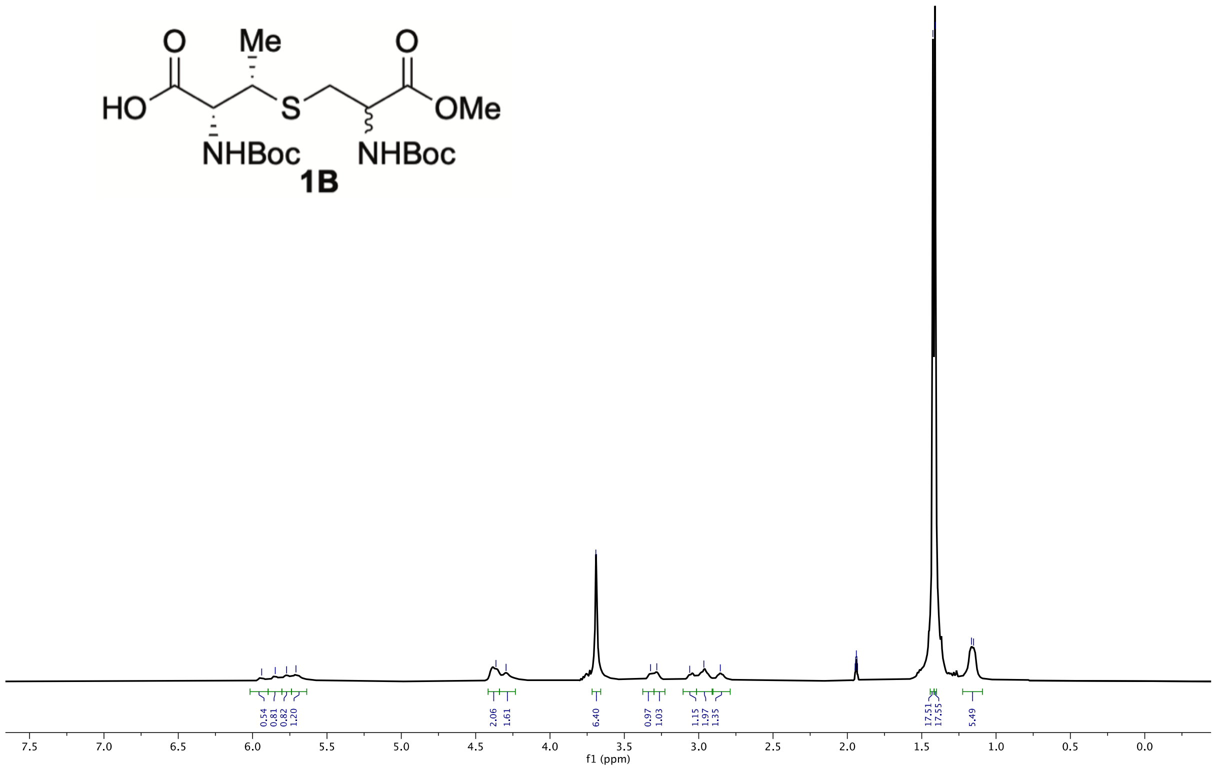

Figure S28. ^{1}H NMR spectrum of **1B** in CD_3CN.

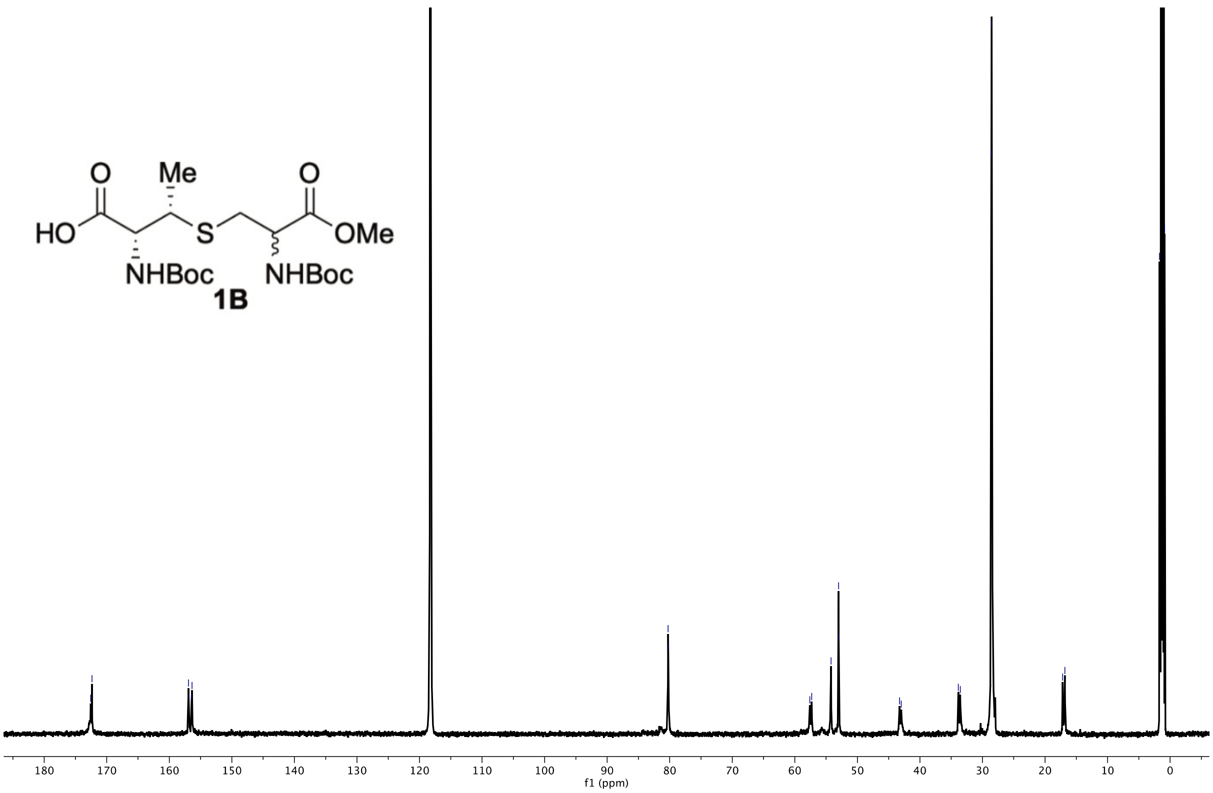

Figure S29. ^{13}C NMR spectrum of **1B** in CD_3CN.

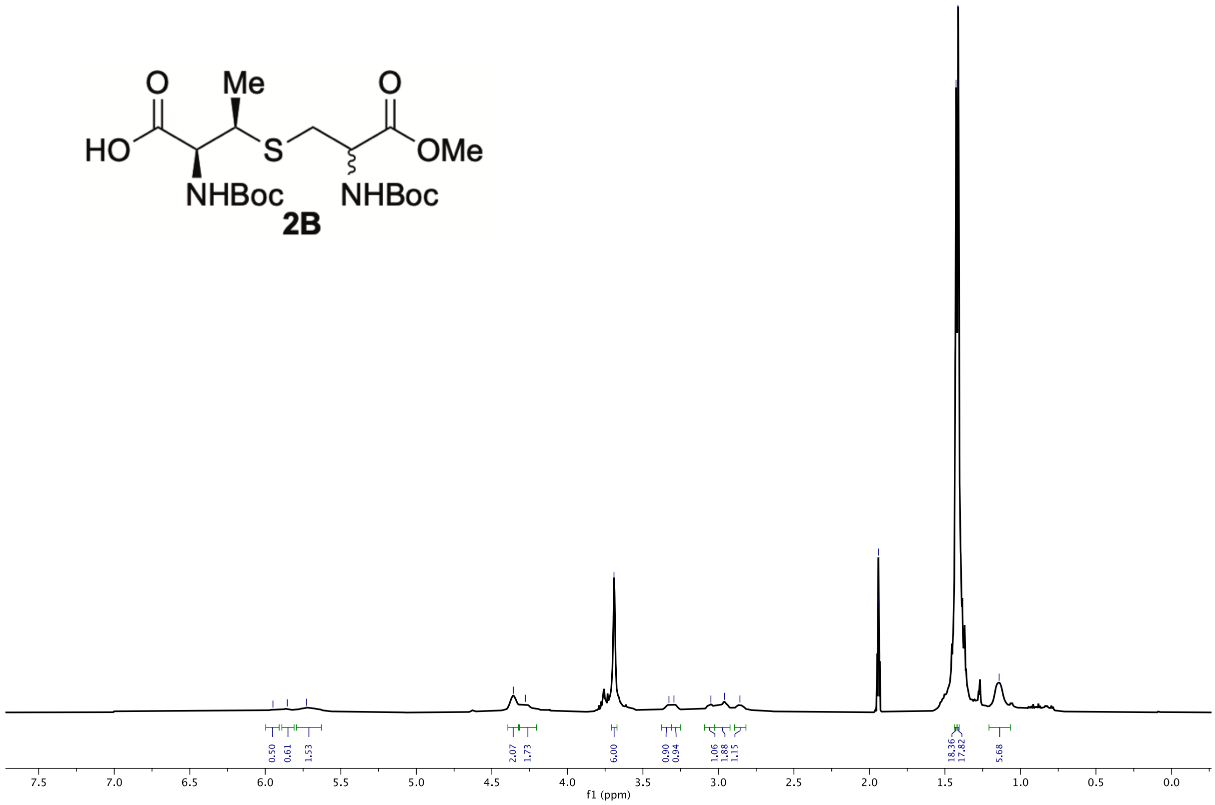

Figure S30. ^{1}H NMR spectrum of **2B** in CD_3CN.

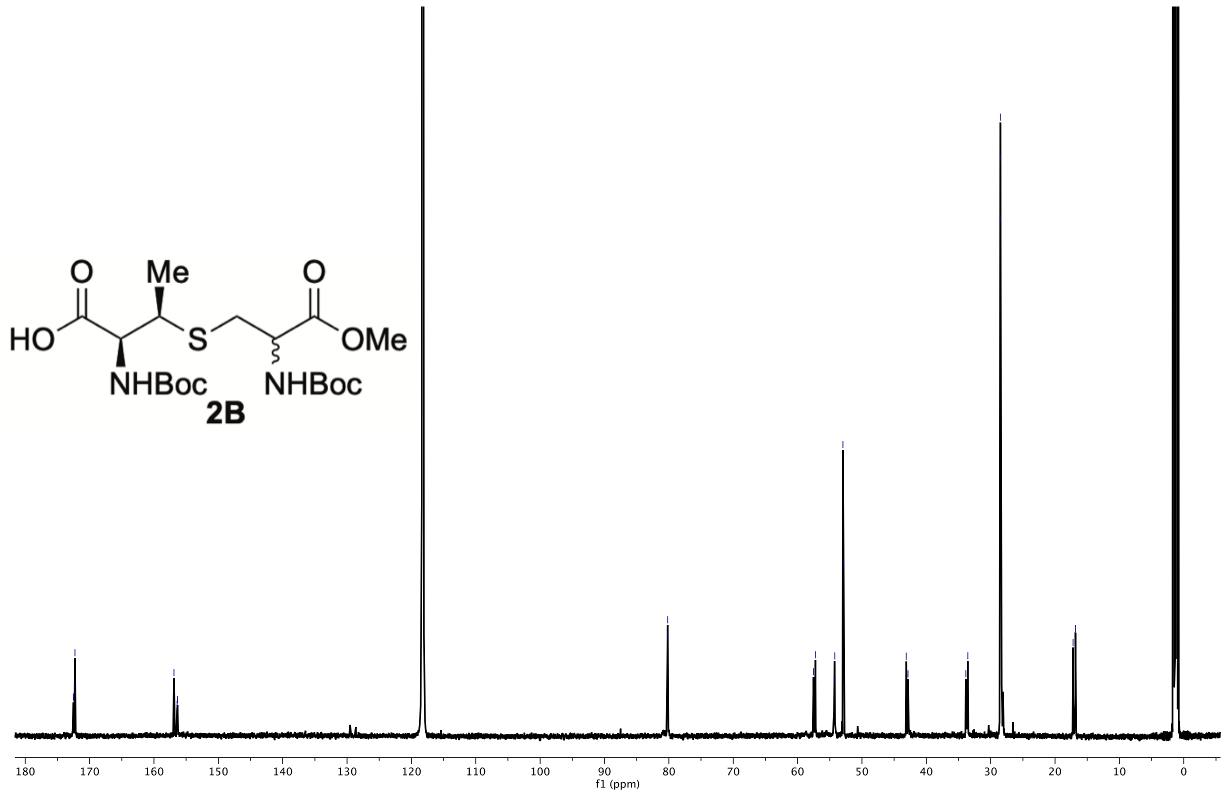

Figure S31. ^{13}C NMR spectrum of **2B** in CD_3CN.

References

1. Tropea, J. E.; Cherry, S.; Waugh, D. S., Expression and purification of soluble His(6)-tagged TEV protease. *Methods Mol. Biol.* **2009,** *498*, 297-307.

2. Ciufo, S.; Kannan, S.; Sharma, S.; Badretdin, A.; Clark, K.; Turner, S.; Brover, S.; Schoch, C. L.; Kimchi, A.; DiCuccio, M., Using average nucleotide identity to improve taxonomic assignments in prokaryotic genomes at the NCBI. *Int. J. Syst. Evol. Microbiol.* **2018,** *68* (7), 2386-2392.

3. Aziz, R. K.; Bartels, D.; Best, A. A.; DeJongh, M.; Disz, T.; Edwards, R. A.; Formsma, K.; Gerdes, S.; Glass, E. M.; Kubal, M.; Meyer, F.; Olsen, G. J.; Olson, R.; Osterman, A. L.; Overbeek, R. A.; McNeil, L. K.; Paarmann, D.; Paczian, T.; Parrello, B.; Pusch, G. D.; Reich, C.; Stevens, R.; Vassieva, O.; Vonstein, V.; Wilke, A.; Zagnitko, O., The RAST Server: rapid annotations using subsystems technology. *BMC Genomics* **2008,** *9*, 75.

4. Overbeek, R.; Olson, R.; Pusch, G. D.; Olsen, G. J.; Davis, J. J.; Disz, T.; Edwards, R. A.; Gerdes, S.; Parrello, B.; Shukla, M.; Vonstein, V.; Wattam, A. R.; Xia, F.; Stevens, R., The SEED and the Rapid Annotation of microbial genomes using Subsystems Technology (RAST). *Nucleic Acids Res.* **2014,** *42* (Database issue), D206-14.

5. Brettin, T.; Davis, J. J.; Disz, T.; Edwards, R. A.; Gerdes, S.; Olsen, G. J.; Olson, R.; Overbeek, R.; Parrello, B.; Pusch, G. D.; Shukla, M.; Thomason, J. A., 3rd; Stevens, R.; Vonstein, V.; Wattam, A. R.; Xia, F., RASTtk: a modular and extensible implementation of the RAST

algorithm for building custom annotation pipelines and annotating batches of genomes. *Sci. Rep.* **2015,** *5*, 8365.
6. Blin, K.; Shaw, S.; Kloosterman, A. M.; Charlop-Powers, Z.; van Wezel, G. P.; Medema, M. H.; Weber, T., antiSMASH 6.0: improving cluster detection and comparison capabilities. *Nucleic Acids Res.* **2021,** *49* (W1), W29-w35.
7. Iftime, D.; Jasyk, M.; Kulik, A.; Imhoff, J. F.; Stegmann, E.; Wohlleben, W.; Süssmuth, R. D.; Weber, T., Streptocollin, a type IV lanthipeptide produced by *Streptomyces collinus* Tü 365. *ChemBioChem* **2015,** *16* (18), 2615-23.
8. Hegemann, J. D.; van der Donk, W. A., Investigation of substrate recognition and biosynthesis in class IV lanthipeptide systems. *J. Am. Chem. Soc.* **2018,** *140* (17), 5743-5754.
9. Hegemann, J. D.; Shi, L.; Gross, M. L.; van der Donk, W. A., Mechanistic studies of the kinase domains of Class IV lanthipeptide synthetases. *ACS Chem. Biol.* **2019,** *14* (7), 1583-1592.
10. Hegemann, J. D.; Süssmuth, R. D., Identification of the catalytic residues in the cyclase domain of the class IV lanthipeptide synthetase SgbL. *ChemBioChem* **2021,** *22* (22), 3169-3172.
11. Ortega, M. A.; Hao, Y.; Walker, M. C.; Donadio, S.; Sosio, M.; Nair, S. K.; van der Donk, W. A., Structure and tRNA specificity of MibB, a lantibiotic dehydratase from Actinobacteria involved in NAI-107 biosynthesis. *Cell Chem. Biol.* **2016,** *23* (3), 370-380.
12. Küsters, E.; Allgaier, H.; Jung, G.; Bayer, E., Resolution of sulphur-containing amino acids by chiral phase gas chromatography. *Chromatographia* **1984,** *18* (6), 287-293.
13. Acedo, J. Z.; Bothwell, I. R.; An, L.; Trouth, A.; Frazier, C.; van der Donk, W. A., *O*-methyltransferase-mediated incorporation of a β-amino acid in lanthipeptides. *J. Am. Chem. Soc.* **2019,** *141* (42), 16790-16801.
14. Tang, W.; Jiménez-Osés, G.; Houk, K. N.; van der Donk, W. A., Substrate control in stereoselective lanthionine biosynthesis. *Nat. Chem.* **2015,** *7* (1), 57-64.
15. Hudson, G. A.; Zhang, Z.; Tietz, J. I.; Mitchell, D. A.; van der Donk, W. A., In vitro biosynthesis of the core scaffold of the thiopeptide thiomuracin. *J. Am. Chem. Soc.* **2015,** *137* (51), 16012-16015.
16. Garg, N.; Tang, W.; Goto, Y.; Nair, S. K.; van der Donk, W. A., Lantibiotics from *Geobacillus thermodenitrificans*. *Proc. Natl. Acad. Sci. U.S.A.* **2012,** *109* (14), 5241-5246.
17. Boratyn, G. M.; Camacho, C.; Cooper, P. S.; Coulouris, G.; Fong, A.; Ma, N.; Madden, T. L.; Matten, W. T.; McGinnis, S. D.; Merezhuk, Y.; Raytselis, Y.; Sayers, E. W.; Tao, T.; Ye, J.; Zaretskaya, I., BLAST: a more efficient report with usability improvements. *Nucleic Acids Res.* **2013,** *41* (Web Server issue), W29-33.
18. Altschul, S. F.; Madden, T. L.; Schaffer, A. A.; Zhang, J.; Zhang, Z.; Miller, W.; Lipman, D. J., Gapped BLAST and PSI-BLAST: a new generation of protein database search programs. *Nucleic Acids Res.* **1997,** *25* (17), 3389-402.
19. Gerlt, J. A.; Bouvier, J. T.; Davidson, D. B.; Imker, H. J.; Sadkhin, B.; Slater, D. R.; Whalen, K. L., Enzyme Function Initiative-Enzyme Similarity Tool (EFI-EST): A web tool for generating protein sequence similarity networks. *Biochim. Biophys. Acta* **2015,** *1854* (8), 1019-37.
20. Bailey, T. L.; Boden, M.; Buske, F. A.; Frith, M.; Grant, C. E.; Clementi, L.; Ren, J.; Li, W. W.; Noble, W. S., MEME SUITE: tools for motif discovery and searching. *Nucleic Acids Res.* **2009,** *37* (Web Server issue), W202-8.

21. Max, J. B.; Pergushov, D. V.; Sigolaeva, L. V.; Schacher, F. H., Polyampholytic graft copolymers based on polydehydroalanine (PDha) – synthesis, solution behavior and application as dispersants for carbon nanotubes. *Polym. Chem.* **2019,** *10*, 3006–3019.
22. Mohr, K. I.; Volz, C.; Jansen, R.; Wray, V.; Hoffmann, J.; Bernecker, S.; Wink, J.; Gerth, K.; Stadler, M.; Müller, R., Pinensins: the first antifungal lantibiotics. *Angew. Chem. Int. Ed.* **2015,** *54* (38), 11254-11258.
23. Altschul, S. F.; Gish, W.; Miller, W.; Myers, E. W.; Lipman, D. J., Basic local alignment search tool. *J. Mol. Biol.* **1990,** *215* (3), 403-10.
24. Tietz, J. I.; Schwalen, C. J.; Patel, P. S.; Maxson, T.; Blair, P. M.; Tai, H. C.; Zakai, U. I.; Mitchell, D. A., A new genome-mining tool redefines the lasso peptide biosynthetic landscape. *Nat. Chem. Biol.* **2017,** *13* (5), 470-478.
25. Ortega, M. A.; Hao, Y.; Zhang, Q.; Walker, M. C.; van der Donk, W. A.; Nair, S. K., Structure and mechanism of the tRNA-dependent lantibiotic dehydratase NisB. *Nature* **2015,** *517* (7535), 509-512.
26. Li, B.; van der Donk, W. A., Identification of essential catalytic residues of the cyclase NisC involved in the biosynthesis of nisin. *J. Biol. Chem.* **2007,** *282*, 21169-75.
27. Yang, X.; van der Donk, W. A., Michael-type cyclizations in lantibiotic biosynthesis are reversible. *ACS Chem. Biol.* **2015,** *10* (5), 1234-1238.
